# 4,5-Dihydro-5-Oxo-Pyrazolo[1,5-a]Thieno[2,3-c]Pyrimidine: A Novel Scaffold Containing Thiophene Ring. Chemical Reactivity and In Silico Studies to Predict the Profile to GABA_A_ Receptor Subtype

**DOI:** 10.3390/molecules28073054

**Published:** 2023-03-29

**Authors:** Letizia Crocetti, Gabriella Guerrini, Fabrizio Melani, Claudia Vergelli, Maria Paola Giovannoni

**Affiliations:** Neurofarba, Pharmaceutical and Nutraceutical Section, University of Florence, 50019 Sesto Fiorentino, Italy; letizia.crocetti@unifi.it (L.C.); fabrizio.melani@unifi.it (F.M.); claudia.vergelli@unifi.it (C.V.); mariapaola.giovannoni@unifi.it (M.P.G.)

**Keywords:** 4,5-dihydro-5-oxo-pyrazolo[1,5-a]thieno[2,3-c]pyrimidines, novel scaffold, thiophene, molecular dynamic studies, proximity frequencies (PF), GABA_A_ receptor subtype, GABA_A_ receptor ligands

## Abstract

The isosteric replacement of the benzene with thiophene ring is a chemical modification widely applied in medicinal chemistry. Several drugs containing the thiophene ring are marketed for treating various pathologies (osteoporosis, peripheral artery disorder, psychosis, anxiety and convulsion). Taking into account this evidence and as a continuation of our study in the GABA_A_ receptor modulators field, we designed and synthesized new compounds containing the thiophene ring with 4,5-dihydro-5-oxo-pyrazolo[1,5-a]thieno[2,3-c]pyrimidine and pyrazolo[1,5-a]thieno[2,3-c] pyrimidine scaffold. Moreover, these cores, never reported in the literature, are isosteres of pyrazolo[1,5-a]quinazolines (PQ), previously published by us as GABA_A_R subtype ligands. We introduced in the new scaffold those functions and groups (esters, ketones, alpha/beta-thiophene) that in our PQ derivatives were responsible for the activity, and at the same time, we have extensively investigated the reactivity of the new nucleus regarding the alkylation, reduction, halogenation and hydrolyses. On the six final designed compounds (**12c**–**f**, **22a,b**) molecular docking and dynamic simulation studies have been performed. The analysis of dynamic simulation, applying our reported model ‘Proximity Frequencies’, collocates with high probability **12c**, **22b**, in the agonist class towards α1β2γ2-GABA_A_R.

## 1. Introduction

**Figure 1:**
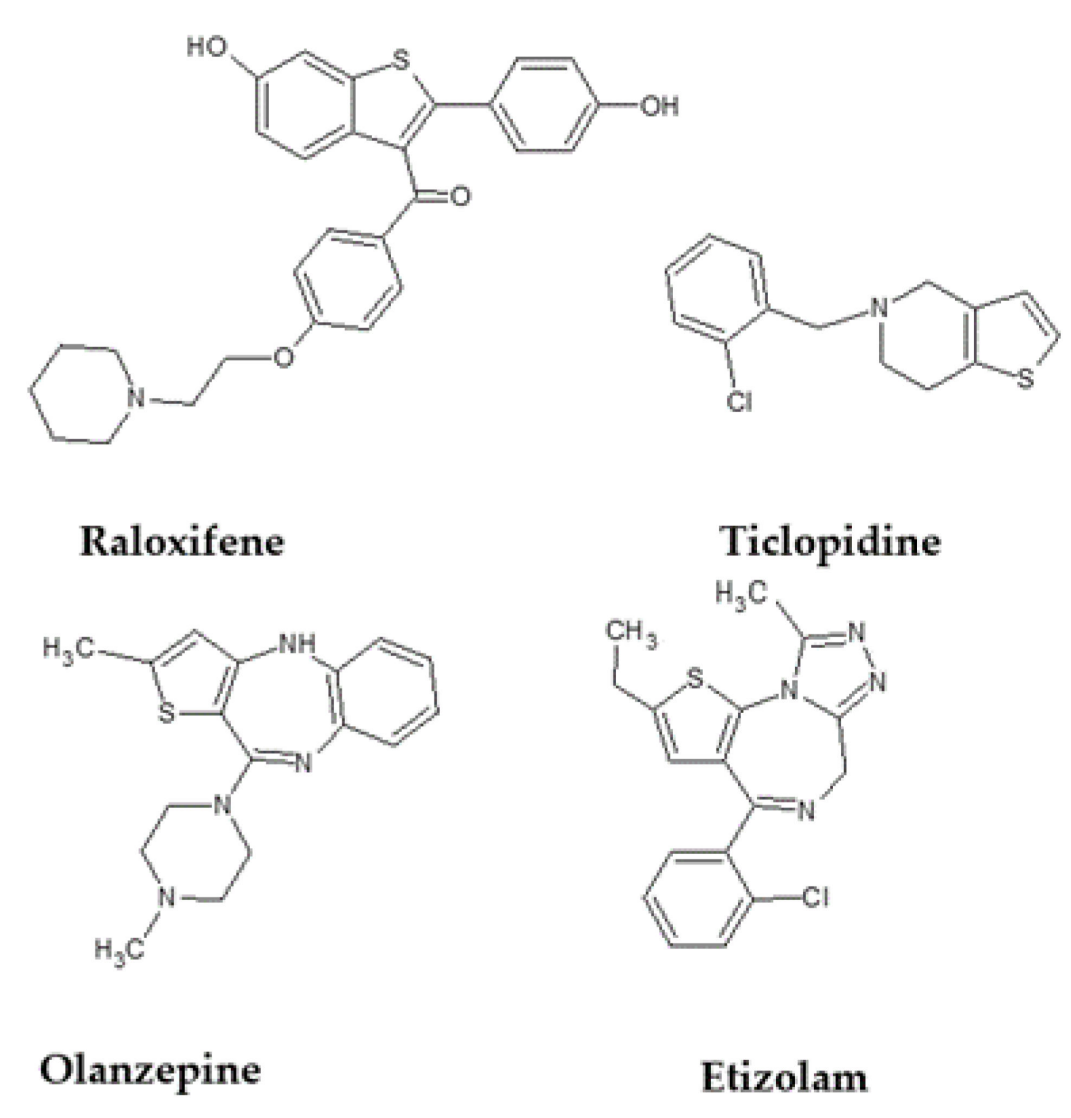
Thiophene-heterocycles-fused compounds known in literature.

Thiophene is known to be an isostere of the benzene ring, thus representing a good tool in the field of medicinal chemistry to develop new drugs. The concept of isosterism applied to the thiophene ring was established in 1932 by Erlenmeyer, which proposed the equivalence between -CH = CH- and -S- (in benzene and thiophene, respectively) in terms of size, mass and capacity to provide an aromatic lone pair. Over time, compounds containing thiophene have been extensively investigated in medicinal chemistry, since they show a variety of pharmacological activities, such as anti-inflammatory, antioxidant, antimicrobial, antitumor, and antidepressant action [1,2,3,4]. Moreover, several thiophene-heterocycles-fused compounds have been approved by FDA as therapeutic agents for the treatment of osteoporosis (Raloxifene), peripheral artery disorder (Ticlopidine), psychosis (Olanzapine), anxiety and convulsion (Etizolam); see Figure 1.

**Figure 2:**
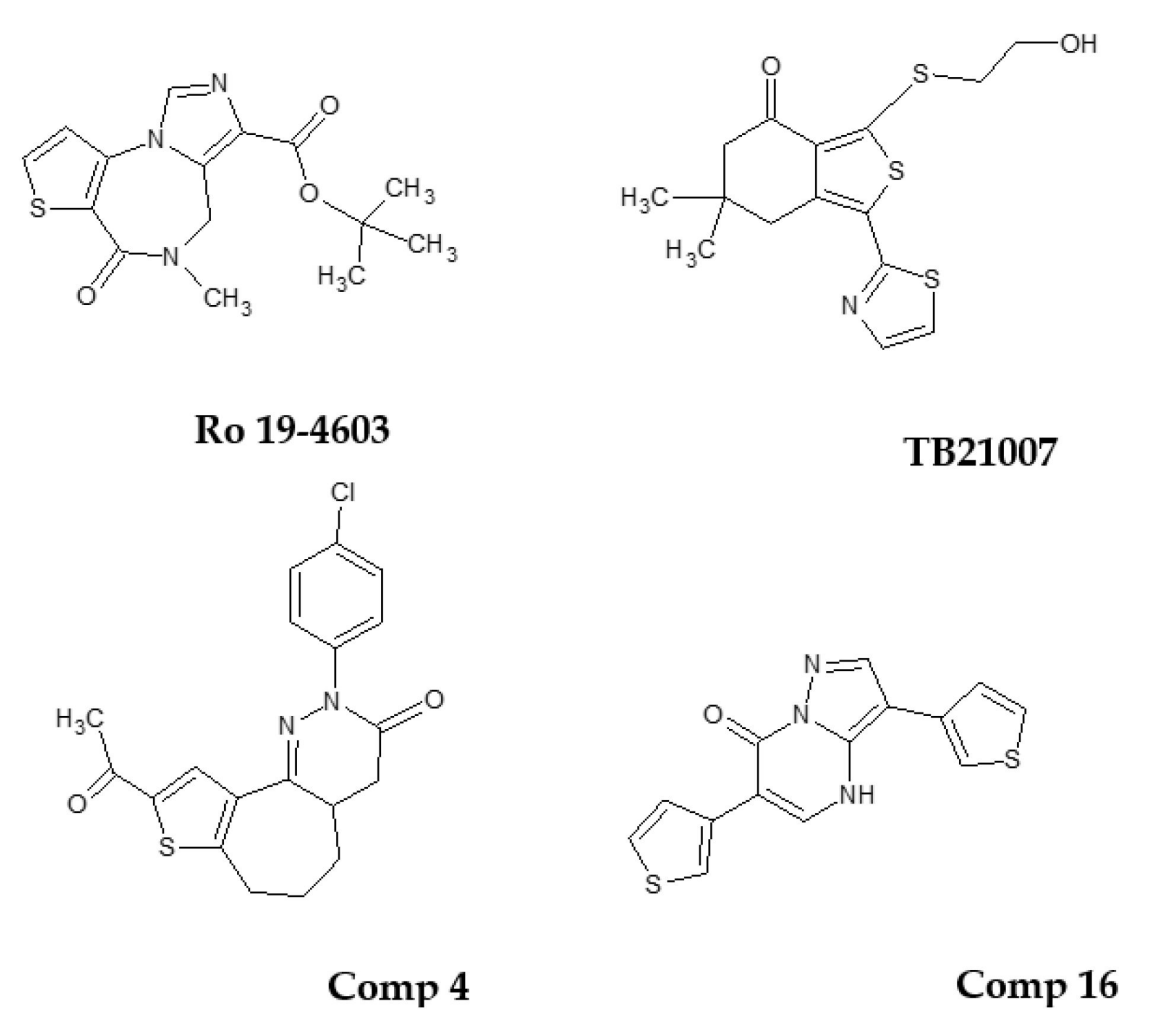
GABA_A_R ligands with a thiophene ring reported in the literature (numbering is that of the original manuscript).

**Figure 3:**
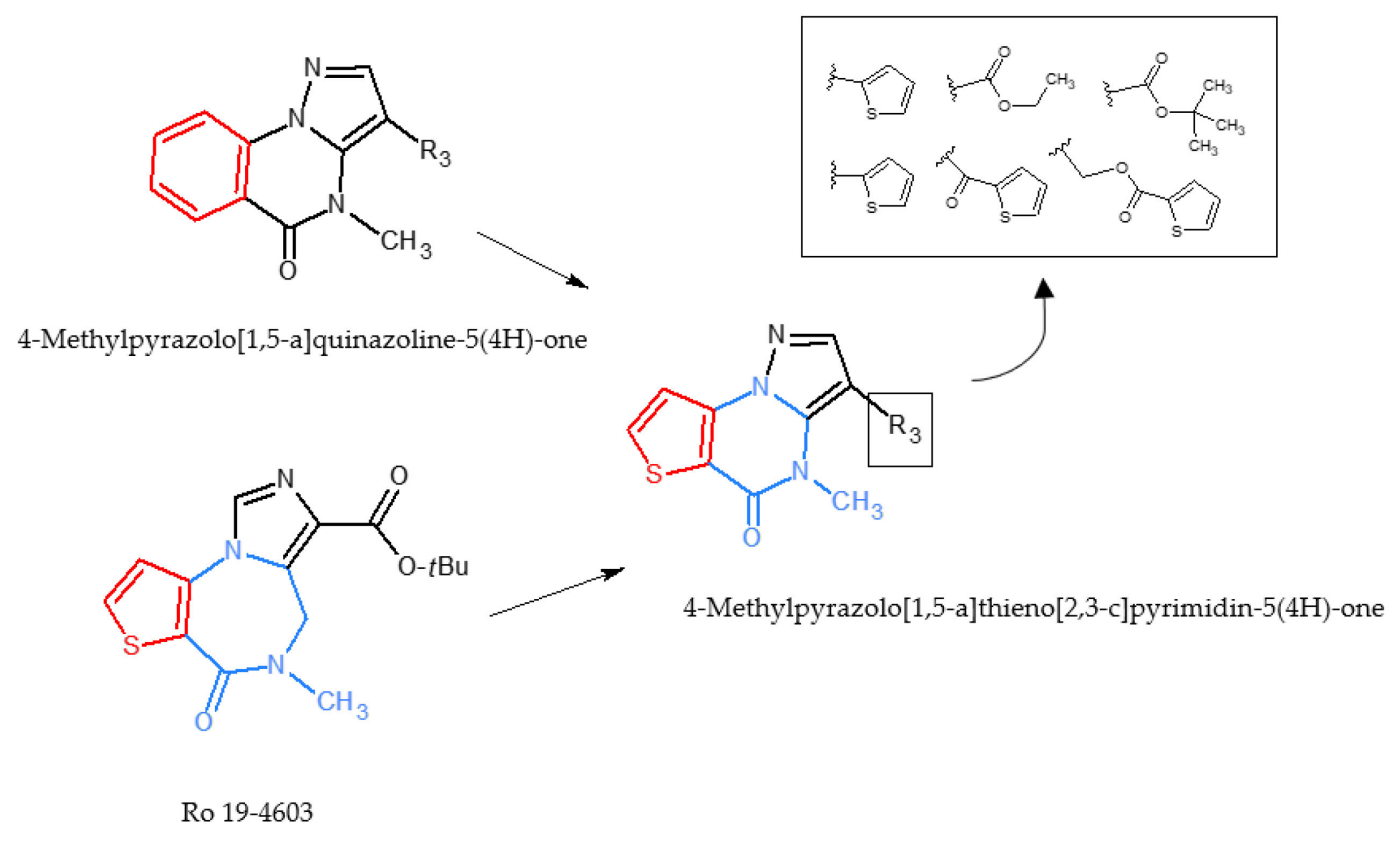
Aim of the work.

Starting from these evidences, we addressed our research towards the synthesis of heterocyclic compounds containing the thiophene ring. We applied this strategy to the synthesis of potential GABA_A_R subtypes ligands, which represent a field of research extensively investigated by us for many years, by obtaining some interesting derivatives with pyrazolobenzotriazine, pyrazolopyrimidine and pirazoloquinazoline scaffold [5,6,7,8]. This choice is also supported by the fact that in the literature are present some GABA_A_R ligands (Ro 19-4603, TB21007, Comp. 4, Comp. 16), in which the thiophene ring is fused or bonded to other cycles (Figure 2) [9,10,11]. In particular, we report here the synthesis of new compounds with 4,5-dihydro-5-oxo-pyrazolo[1,5-a]thieno[2,3-c]pyrimidine and pyrazolo[1,5-a]thieno[2,3-c]pyrimidine scaffold as result of the isosteric replacement of the benzene with thiophene ring in our pyrazolo[1,5-a]quinazolines previously published as GABA_A_R subtype ligands [8,12] (Figure 3). Moreover, these new compounds can be considered as analogues of the abovementioned GABA_A_R ligand Ro 19-4603, in which we formally operated a contraction of the central diazepine ring. As a first approach, in this new scaffold, we tried to introduce at position 3 those functions and groups (esters, ketons, alpha/beta-thiophene) responsible for activity in our pyrazolo[1,5-a]quinazolines and in compounds of the literature (i.e., Ro 19-4603 and Comp 16) (Figure 2).

The pyrazolo[1,5-a]thieno[2,3-c]pyrimidine core is not found in the literature (SciFinder, Reaxys), with the exception of the ethyl 4,5-dihydro-5-oxo-pyrazolo [1,5-a]thieno[2,3-c]pyrimidine-3-carboxylate (RN 942034-93-7), of which neither the synthesis nor the characterization is reported, however. In addition, this single compound is not mentioned in any published work. Thus, it was very intriguing to investigate the feasibility and the reactivity of this nucleus toward the most common reactions, such as alkylation, reduction, halogenation, and hydrolysis. Finally, molecular docking studies and evaluation of the ‘Proximity Frequencies’ (exploiting our reported model) [8,13] were performed on all the final compounds to predict their profile on the α1β2γ2-GABAAR subtype.

## 2. Results

### 2.1. Chemistry

**Scheme 1:**
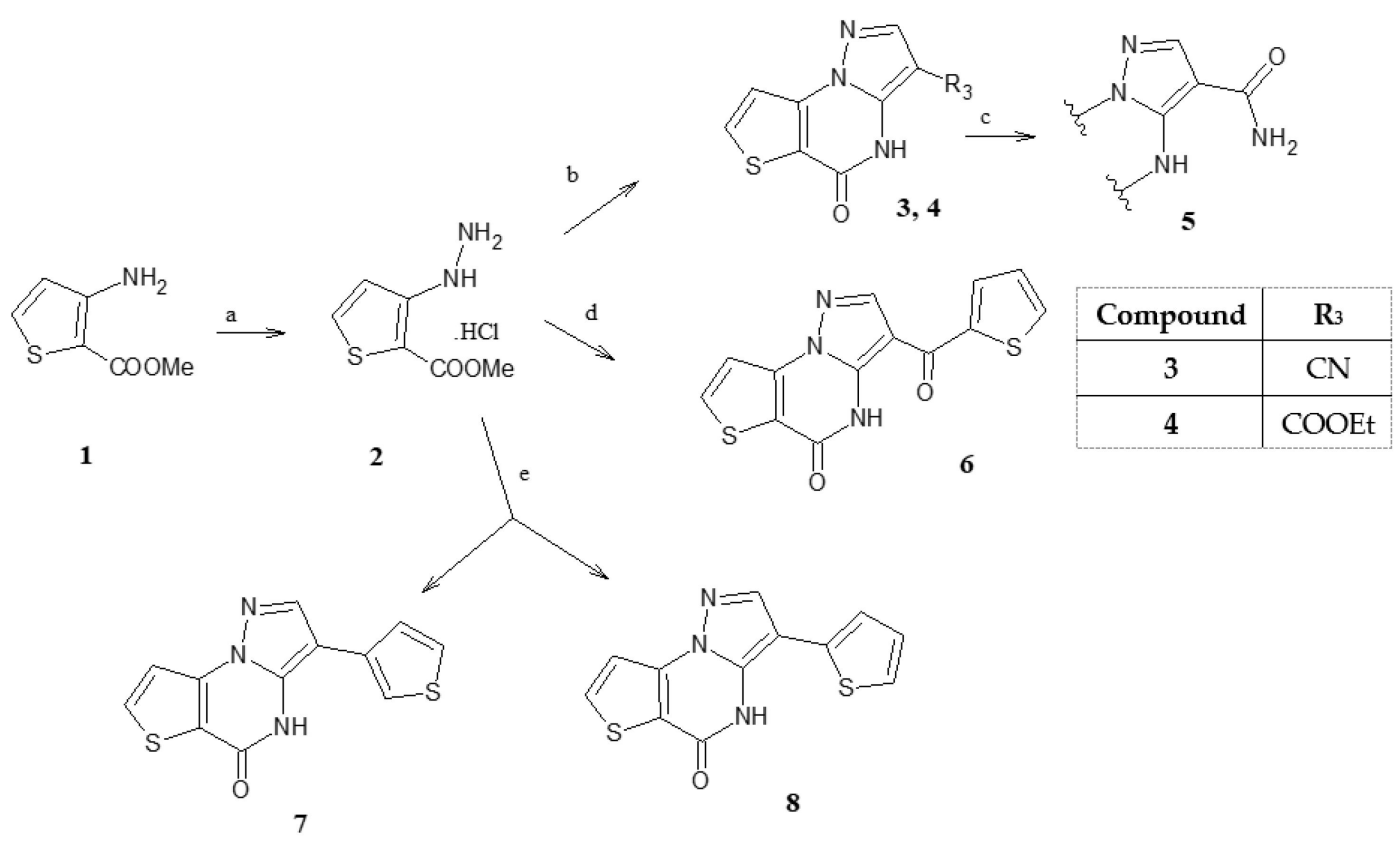
Reagent and conditions: (a) NaNO_2_/H_2_O, HCl, SnCl_2_, 0 °C; (b) ethoxymethylenmalononitrile for **3** and ethyl 2-cyanoethoxyacrylate for **5**, DMF/AcONa, refluxing temperature; (c) H_2_SO_4_, 80 °C; (d) 3-(dimethylamino)-2-(thien-2-ylcarbonyl)acrylonitrile, DMF/AcONa, refluxing temperature; (e) 3-oxo-2-(thien-3-yl)propionitrile for **7** and 3-oxo-2-(thien-2-yl)propionitrile for **8**, AcOH, refluxing temperature.

**Scheme 2:**
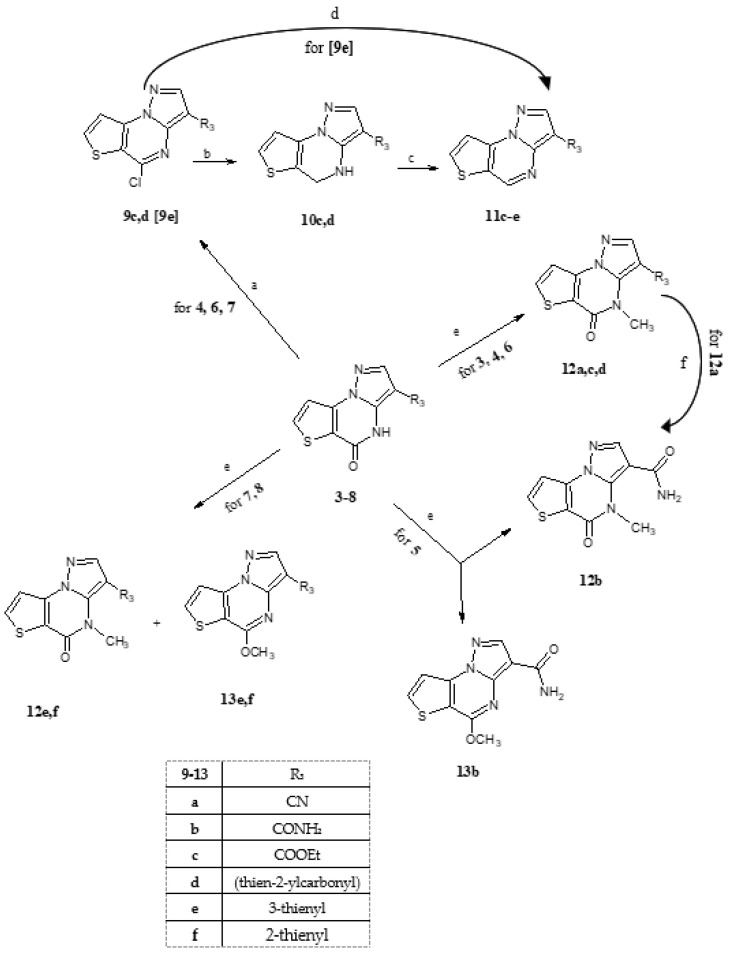
Reagent and conditions: (a) POCl_3_/PCl_5_ refluxing temperature; (b) NaBH_4_/EtOH/CH_2_Cl_2_, RT°, 40 min; (c) Toluene, Pd/C, refluxing temperature for **10c**,**d**; (d) HCOONH_4_/EtOH, Pd/C, refluxing temperature for **9e**; (e) DMF/K_2_CO_3_/CH_3_I, 80 °C for **12a**,**c**,**d**–**f** and **13b**,**e**,**f**; at reflux temperature for **12b**; (f) H_2_SO_4_ conc.

**Scheme 3:**
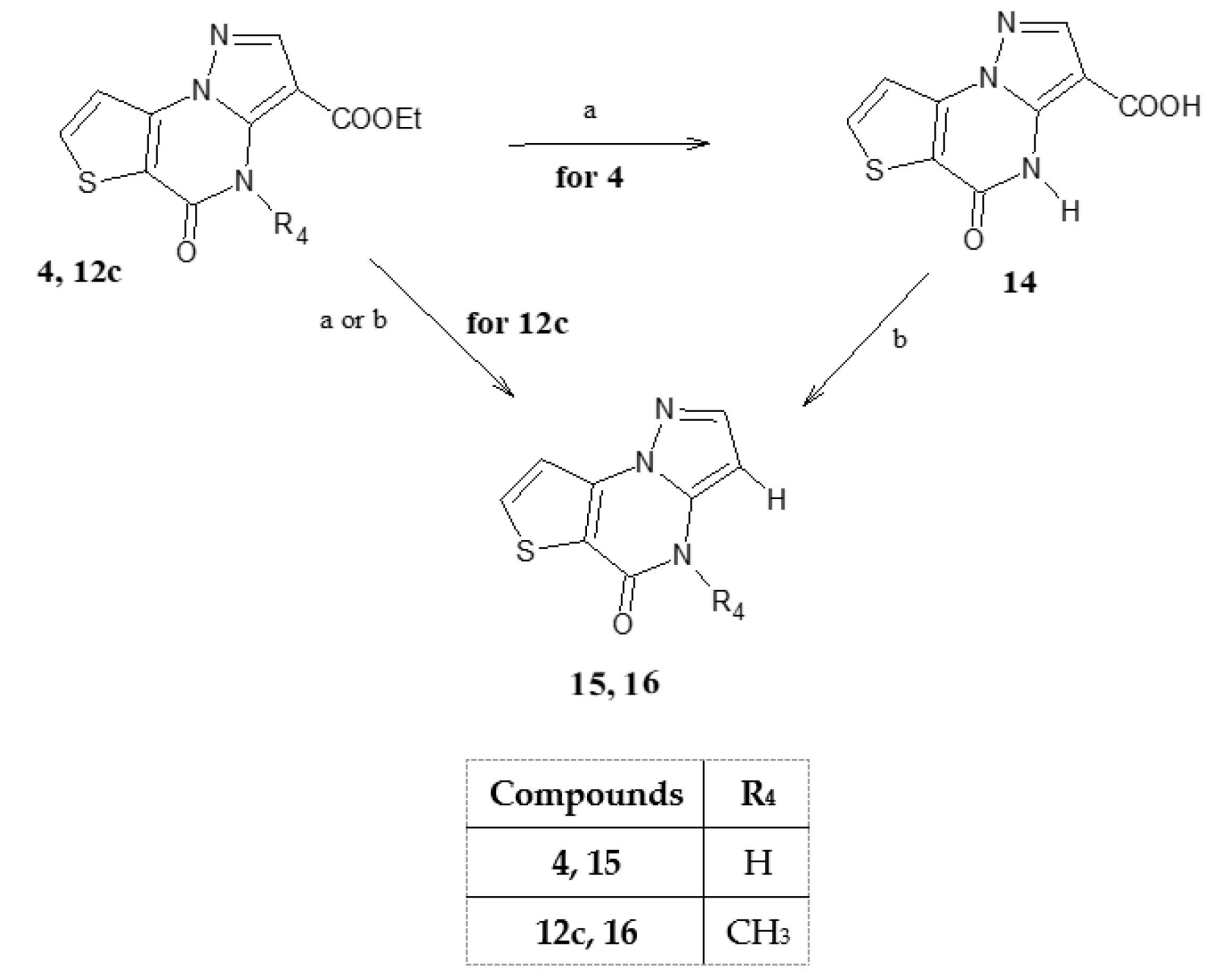
Reagent and conditions: (a) for **4** NaOH 10% solution or HCl conc.; for **12c** NaOH 10% solution or LiOH or HCl conc.; (b) HCl conc. reflux temperature.

**Scheme 4:**
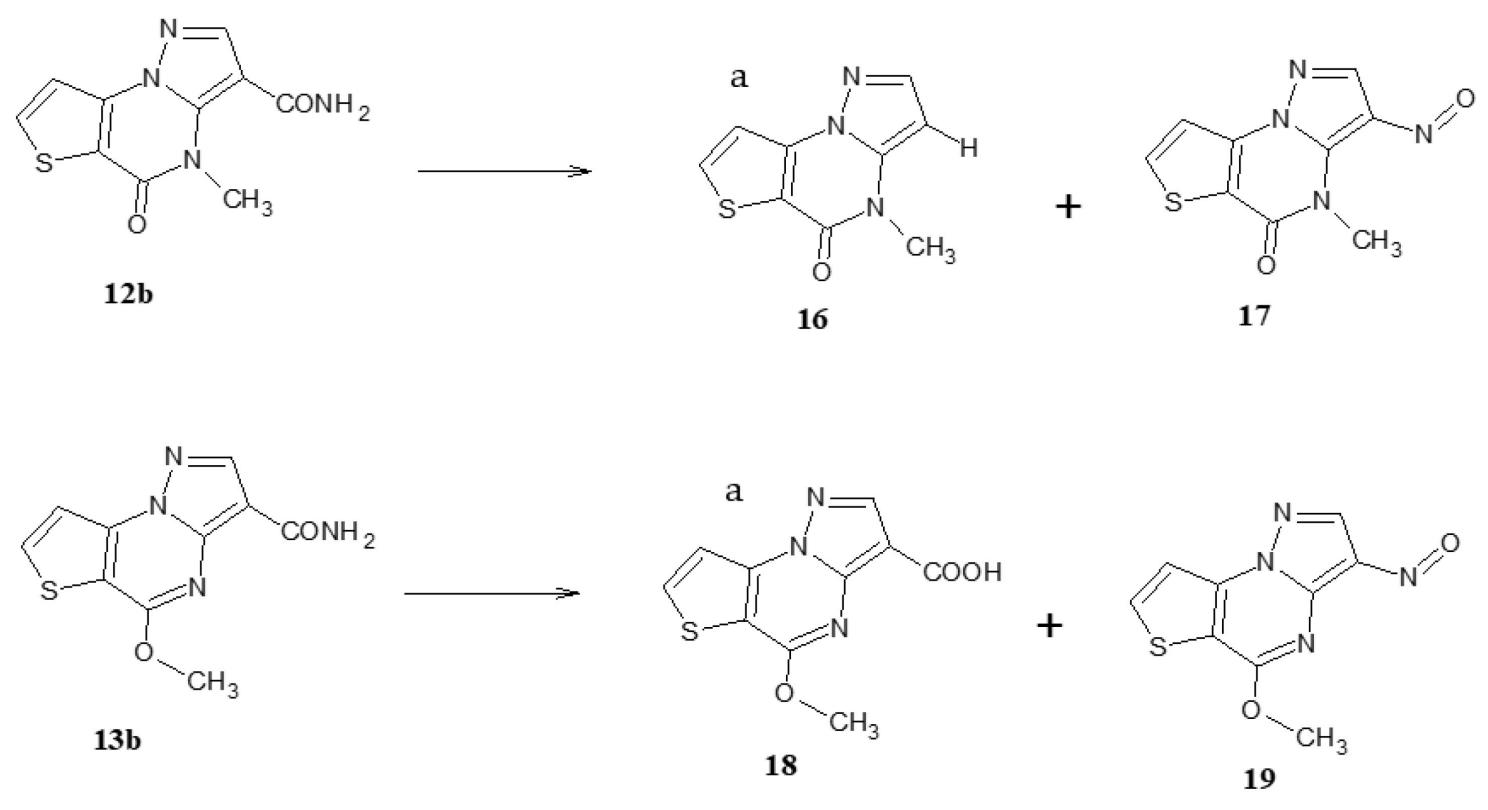
Reagent and conditions: (a) H_2_SO_4_ conc., NaNO_2_/H_2_O, RT for 12 h.

**Scheme 5:**
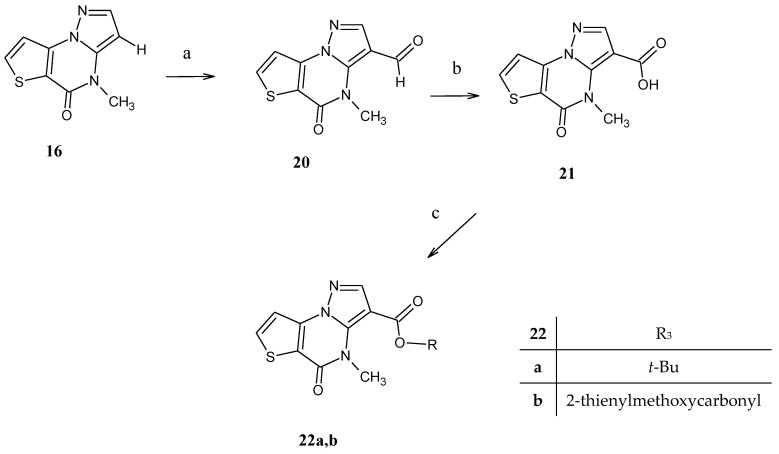
Reagent and conditions: (a) AcOH/HMTA, 80 °C; (b) aceton/water, KMnO_4_, NaOH 10%, 80 °C; (**c**) SOCl_2_, CH_2_Cl_2_, t-BuOH for **22a** and 2-thiophenemethanol for **22b**, 50 °C.

**Scheme 6:**
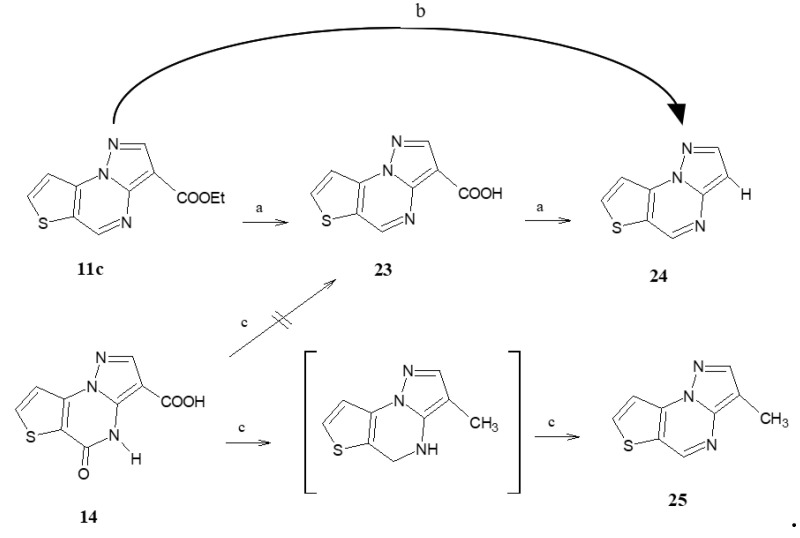
Reagent and conditions: (a) NaOH 10% solution, or LiOH; (b) HCl or AcOH; (c) LiAlH4, THF abs., then: toluene, Pd/C, refluxing temperature.

The synthetic pathways for obtaining derivatives with pyrazolo[1,5-a]thieno[2,3-e]pyrimidine scaffold are depicted in Schemes 1–6. In this synthetic section, we report not only the procedures for obtaining the final designed products mentioned in Figure 3, but also some reactivity studies on this new scaffold which, furthermore, have produced interesting results. NMR spectra, elemental analysis and other structural information are reported in Supplementary Materials, Table S1.

The first step in building the pyrazolo[1,5-a]thieno[2,3-c]pyrimidine core is a diazotization reaction in the usual manner followed by reduction with SnCl_2_, on the commercial material methyl 3-aminothiophene-2-carboxylate **1**, obtaining the corresponding 3-hydrazino hydrochloride derivative **2** [14]. This latter was then reacted with ethoxymethylenmalononitrile and ethyl 2-cyano-3-ethoxyacrylate, affording the pyrazolo[1,5-a]thieno[2,3-e]pyrimidin-5(4H)-ones 3-carbonitrile and 3-ethoxycarbonyl derivative **3** and **4**, respectively; further hydrolysis of **3** with H_2_SO_4_ conc. gave the 3-carboxamide derivative **5**. Compounds **6**–**8** were instead obtained by treatment of the same 3-hydrazino hydrochloride derivative **2** with 3-(dimethylamino)-2- (thien-2-carbonyl)acrylonitrile (compound **6**), 3-oxo-2-(thien-3-yl)propionitrile (compound **7**), and 3-oxo-2-(thien-2-yl)propionitrile (compound **8**), respectively, following the procedure reported in our references [15,16] (Scheme 1).

All the **3***–***8** pyrazolo[1,5-a]thieno[2,3-e]pyrimidin-5(4H)-ones intermediates synthesized in Scheme 1 were then subjected to halogenation, reduction and alkylation reactions (Scheme 2) and the results differed depending on the function/group bound at position 3 (**3**, R_3_ = CN; **4**, R_3_ = COOEt; **5**, R_3_ = CONH_2_; **6**, R_3_ = thien-2-yl carbonyl; **7**, R_3_ = 3-thienyl; **8**, R_3_ = 2-thienyl). Starting from the halogenation reaction, the treatment with POCl_3_/PCl_5_ only for compounds **5** and **6** gave the corresponding 5-chloro derivatives **9c** and **9d**, which were effortlessly isolated and purified. From compound **7**, the intermediate 5-chloro derivative results as one spot in TLC but was not isolated (**9e**) and used as such for the subsequent reduction reaction. Differently, for compounds bearing a cyano group (**3**) or a thienyl ring at the 3-position (**7** and **8**), the 5-chloro derivatives were not obtained or were not easily isolable. In particular, from compounds **3** and **8** dark tares resulted in TLC many spots of complex purification. For the next step, involving the C-Cl bond cleavage and the resulting reduction to the 4,5-dihydro derivative **10c**,**d**, we choose NaBH_4_ in the EtOH/CH_2_Cl_2_ mixture. The ethyl 5-chloropyrazolo[1,5-a]thieno [2,3-e]pyrimidine-3-carboxylate **9c** and the 3-(thien-2-ylcarbonyl)-5-chloropyrazolo [1,5-a]thieno[2,3-e]pyrimidine **9d** were rapidly transformed into the final compounds **10c**,**d**, which were easily recovered and purified; the (hetero) aromatization was then realized by treating **10c** and **10d** in toluene with Pd/C at refluxing temperature, obtaining the final products **11c** and **11d**.

A particular reactivity was evidenced for not isolated intermediate **9e**, the 3-(thien-3-yl)-5-chloropyrazolo[1,5-a]thieno[2,3-e]pyrimidine. In fact, when the reduction is performed with NaBH_4_, starting and final products mixture was anyway recovered, also largely changing the reaction conditions. On the other hand, performing a catalytic transfer hydrogenation (CTH) with HCOONH_4_ and Pd/C in EtOH [17], it was possible to highlight in TLC a spot that could be the 4,5-dihydro derivate, which rapidly and spontaneously converts into the 3-(thien-3-yl)pyrazolo[1,5-a]thieno[2,3-e]pyrimidine **11e**. Moving to the alkylation reactions, which afforded the final desired compounds **12c**–**f**, the lactam derivatives (**3***–***8**) were all treated following the classical method (DMF/K_2_CO_3_/CH_3_I), but depending on the substituent at the 3-position, different reactivity was evidenced, specifically:

- Compounds **3**, **4** and **6** gave in good yield the 4-methyl derivatives **12a,c,d** (4-methyl-5-oxo-4,5-dihydropyrazolo[1,5-a]thieno[2,3-e]pyrimidine-3-carbonitrile **12a**, 3-ethyl carboxylate **12c** and 3-(thien-2-ylcarbonyl) **12d**).
- The 3-carboxamide derivative **5** gave different reaction products depending on the reaction conditions, in particular on the temperature. In fact, at 80 °C (standard condition), only the O-methyl derivative **13b** (5-methoxypyrazolo[1,5-a]thieno[2,3-e] pyrimidine-3-carboxamide) was obtained, while when maintaining the reaction at reflux temperature, the N-methyl isomer **12b** was obtained as a single product. The 3-carboxyamide derivative **12b** was also obtained by treatment of 4-methyl-5-oxo-4,5-dihydropyrazolo[1,5-a]thieno[2,3-e]pyrimidine-3-carbonitrile **12a** with conc. H_2_SO_4_, and thus, confirming the N-alkylation.
- Finally, the alkylation of **7** and **8** in the usual manner afforded a mixture of the N-methyl and O-methyl derivatives (**12e** and **13e**; **12f** and **13f**), which were separated by flash chromatography. The assignment of the exact structure was carried out with the use of ^1^H-NMR technique, whose results are in agreement with our previous data [8]: when methyl is bound to N-4, the peak falls between 3.33 and 4.00 ppm, depending on the substituent at 3-position, while the O-methyl is unchanged for all compounds at 4.20 ppm.

**Figure 4:**
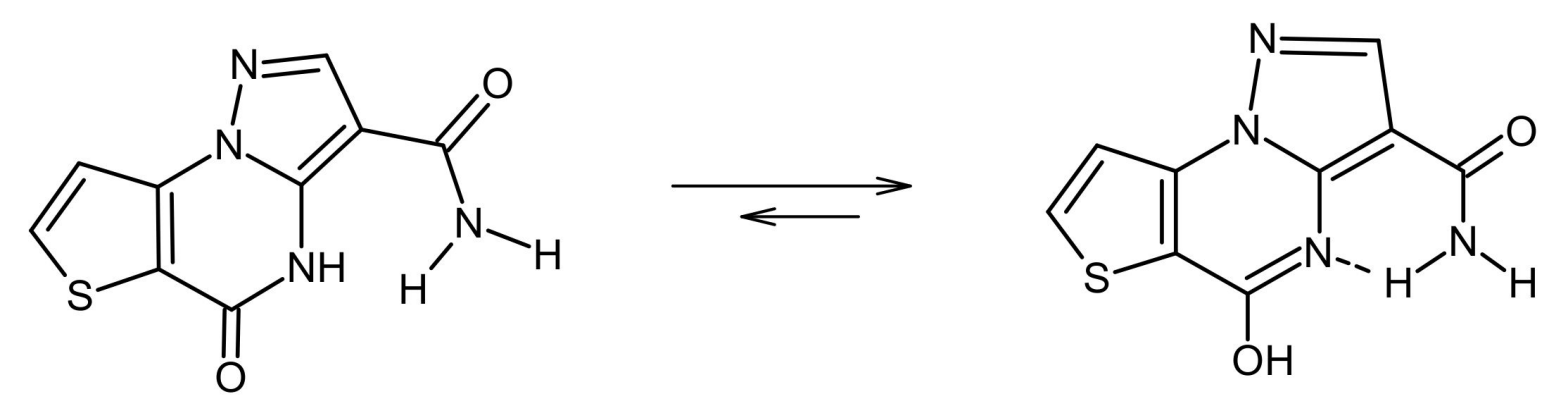
A possible explanation for O-alkylation.

The regioselective O-alkylation observed for compound **5** could be due to a prevalence of the tautomeric form −N = C-OH with respect −NH-C = O; the predominance of the first one could indeed be associated with the amide function at position 3, since the CONH_2_ group could form H-bonds with N-4, thus favoring the tricyclic heteroaromatic structure (see Figure 4). On the other hand, the rising temperature (reflux) can promote a free rotation of the C3-CO bond of the amide group, no longer involved in an H-bond, and thus, allowing alkylation at N-4 (compound **12b**).

Scheme 3 describes the different reactivity of the 3-ethyl carboxylate derivatives **4** and **12c** towards the alkaline or acid hydrolysis. The starting ethyl 5-oxo-4,5-dihydropyrazolo[1,5-a]thieno[2,3-e]pyrimidine-3-carboxylate **4** behaves in the usual manner to alkaline or acid hydrolysis, giving the corresponding 3-carboxylic acid **14,** which in turn undergoes decarboxylation in HCl 12M, at reflux temperature, yielding compound **15**. Instead, starting from the ester **12c**, also using different reaction conditions (NaOH 10% or LiOH in THF/water or AcOH/HCl or conc. HCl), it has never been possible to obtain the 3-carboxylic acid, but only the 3-decarboxylate derivative **16** is recovered.

**Figure 5:**
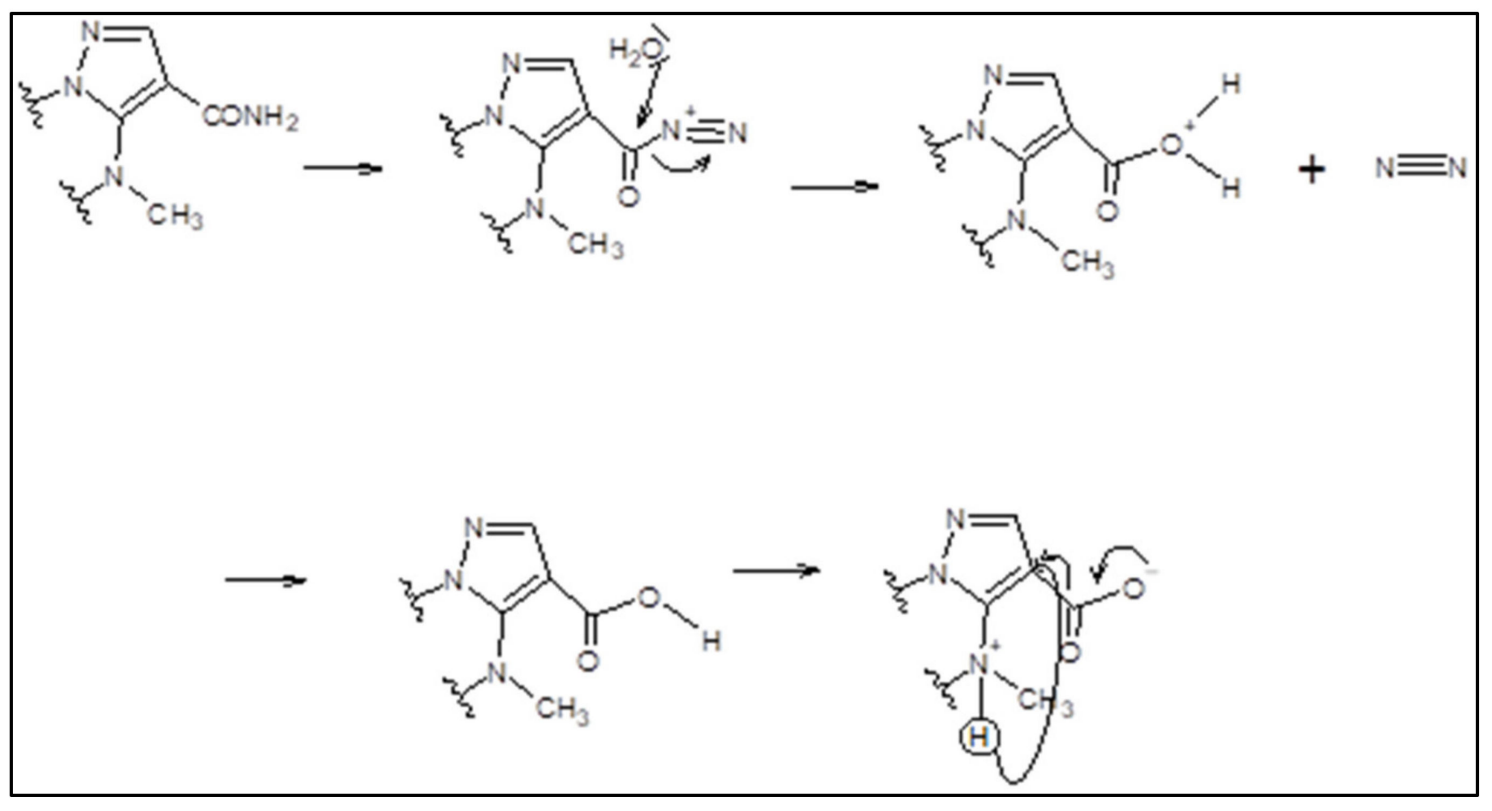
A possible mechanism of decarboxylation.

Thus, to get the 4-methyl-5-oxo-4,5-dihydropyrazolo[1,5-a]thieno[2,3-e] pyrimidine- -3-carboxylic acid, as a key intermediate for obtaining the final designed esters, we followed a reported procedure [18], which involves diazotization of the 3-carboxamide **12b** (Scheme 4); the reaction did not afford the desired 3-carboxylic acid, but a mixture of two compounds, one identified as the 3-decarboxylate **16** and a possible mechanism of decarboxylation process is reported. The second compound, colored green, was assumed to be a 3-nitroso derivative **17**; this hypothesis could be supported by the fact that, after decarboxylation, a high concentration of nitrosonium ion (NO^+^) in the chemical environment could be able to make an electrophilic attach at position 3 of the pyrazolothienoquinazoline scaffold. The ^1^H-NMR spectrum of the supposed compound **17**, in addition to the absence of the proton at position 3, shows a shift of the methyl bound to N-4 (4.23 ppm) that is not consistent with the chemical shift values of the 4-methyl-5-oxo-4,5-dihydropyrazolo[1,5-a]thieno[2,3-e]pyrimidine derivatives, whose N-methyl group constantly falls in the 3.3–3.7 ppm range. Moreover, the chemical shift of the proton in position 2 is lower than in other products with different substituents (CN, COOEt, CONH_2_, COOH), suggesting a different electronic/steric environment resulting from the substituent in position 3. The mass analysis confirmed the structure of compound **17**. The same reaction performed on the 5-methoxypyrazolo [1,5-a]thieno[2,3-e]pyrimidine-3-carboxyamide **13b** also gave a mixture of two products, but in this case, the green 3-nitroso derivative **19** was obtained together with the desired 5-methoxypyrazolo[1,5-a]thieno[2,3-e]pyrimidine-3-carboxylic acid (**18**). The mass analysis again confirmed the structure. A possible mechanism of decarboxylation is reported in Figure 5.

In order to obtain the desired 4-methyl-5-oxo-4,5-dihydropyrazolo [1,5-a]thieno[2,3-e]pyrimidine-3-carboxylic acid **21**, we explored a further synthetic strategy reported in Scheme 5. The 3-unsubstituted compound **16** was treated with HMTA obtaining the 4-methyl-5-oxo-4,5-dihydropyrazolo[1,5-a]thieno[2,3-e]pyrimidin- 3-carboxaldehyde derivative **20** and its further oxidation (KMnO_4_/water/acetone/sodium hydroxide) finally gave the desired 3-carboxylic acid **21**. From acid **21**, the final desired esters **22a,b** were obtained by treatment with thionyl chloride and further addition of the suitable alcohol (t-BuOH and 2-thiophenemethanol respectively) in CH_2_Cl_2_.

The hydrolysis of the ester function to carboxylic acid also created problems on the ethyl pyrazolo[1,5-a]thieno[2,3-e]pyrimidine-3-carboxylate **11c** (Scheme 6). In fact, the desired product **23** was recovered in a meagre yield together with a big amount of the 3-decarboxylate **24** only if the hydrolysis of **11c** was performed in an alkaline medium; the reaction then evolved spontaneously towards the total formation of compound **24**. On the contrary, in acid medium, compound **11c** underwent a decarboxylation, directly giving the 3-unsubstituted derivative **24**.

Therefore, we explored a different synthetic way, starting from the 5-oxo-4,5-dihydropyrazolo[1,5-a]thieno[2,3-e]pyrimidin-3-carboxylic acid **14**, by using LiAlH_4_ as reducing agent for the lactam function. Thus, after quenching the reaction and performing the standard workup, the residue was refluxed in toluene and Pd/C. The presence in ^1^H-NMR spectrum of H5 at 9.06 ppm confirmed that the pyrazolothienopyrimidine core was indeed dehydrogenated but, at the same time, the carboxylic function was transformed in a methyl group, as evidenced by the peak at 2.33 ppm, compound **25**, again preventing the desired **23**.

### 2.2. Molecular Dynamic Studies

On the six final designed compounds (**12c**–**f** and **22a**,**b**), a molecular docking study and an evaluation of the ‘Proximity Frequencies’ [8,13] were performed to predict their profile on the α1β2γ2-GABA_A_R subtype.

The value of Proximity Frequencies (PFs), used in a linear discriminant function (LDA), was able to correctly collocate 70.6% of agonists and 72.7% of antagonists by combining a double PF (αVal203-γThr142) with a triple PF (αHis102-αTyr160-γTyr58). The predictive capacity was evaluated on an appropriate training set of molecules with a cross-validation ‘leave one out’ (LOO) procedure. During a molecular dynamic simulation (60 ns), the agonist compounds were simultaneously close to the αVal203 and γThr142 amino acids, with a frequency of 37% compared to the frequency of 16% found by the antagonist compounds, while the antagonist compounds were simultaneously close to the αHis102, αTyr160, and γTyr58 amino acids, with a frequency of 35% against a frequency of 13% for agonist compounds. All the 3D structures of the molecules, as a training set and new final compounds, were designed [19] (DS ViewerPro 6.0 Accelrys Software Inc., San Diego, CA, USA) and placed in the binding site of the BDZs with the AUTODOCK 4.2 [20] docking program. The structure of the BDZ binding site was obtained from the recently solved GABA_A_R structure (PDB ID 6D6T) [21].

The docking program performed on the selected compounds (**12c**–**f** and **22a**,**b**) gave a number of clusters of conformation(s) for each compound (rmsd 2.0). The evaluation of trajectories in the dynamic simulation was performed on the conformations that covered at least 90% of poses; the dynamic simulations were performed on an isolated portion of the protein between the α and γ chains comprising all amino acids within a radius of 2 nm from the center of the benzodiazepine binding site. Applying the PF model to the new selected compounds, it emerges that all six ones are collocated in the agonist class, **12d**–**f** with low probability, while **12c** and **22b**, the 3-ethyl and the 3-(2-thienylmethyl)carboxylate, respectively, with a percentage of prediction of 74% and 78%, are more probable. The 3-t-buthylcarboxylate **22a** shows a percentage of prediction slightly lower (69%), but always in the agonist class; see Table 1.

**Figure 6:**
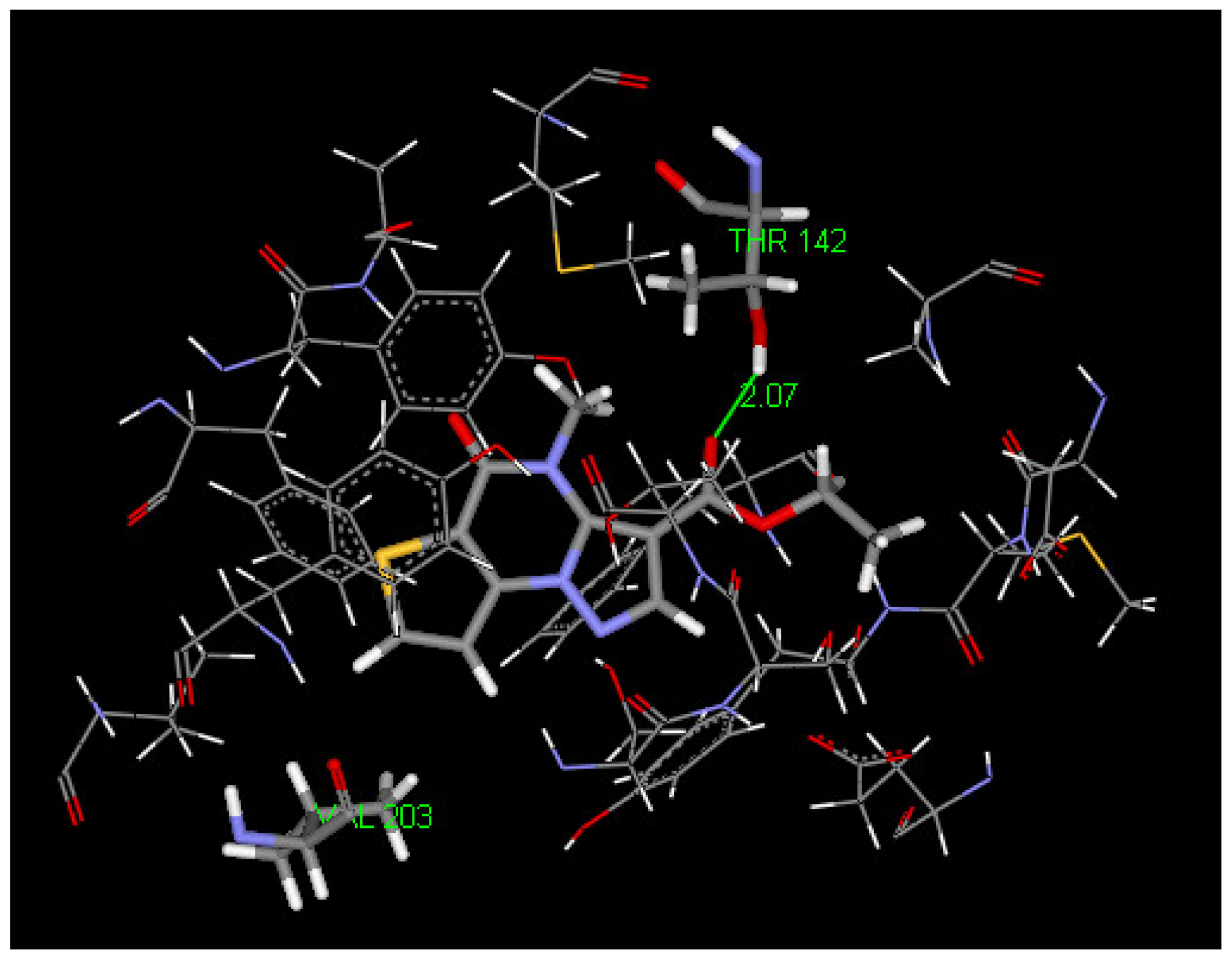
Hydrogen bond interaction of compound **12c** in the last frame of dynamic simulation.

**Figure 7:**
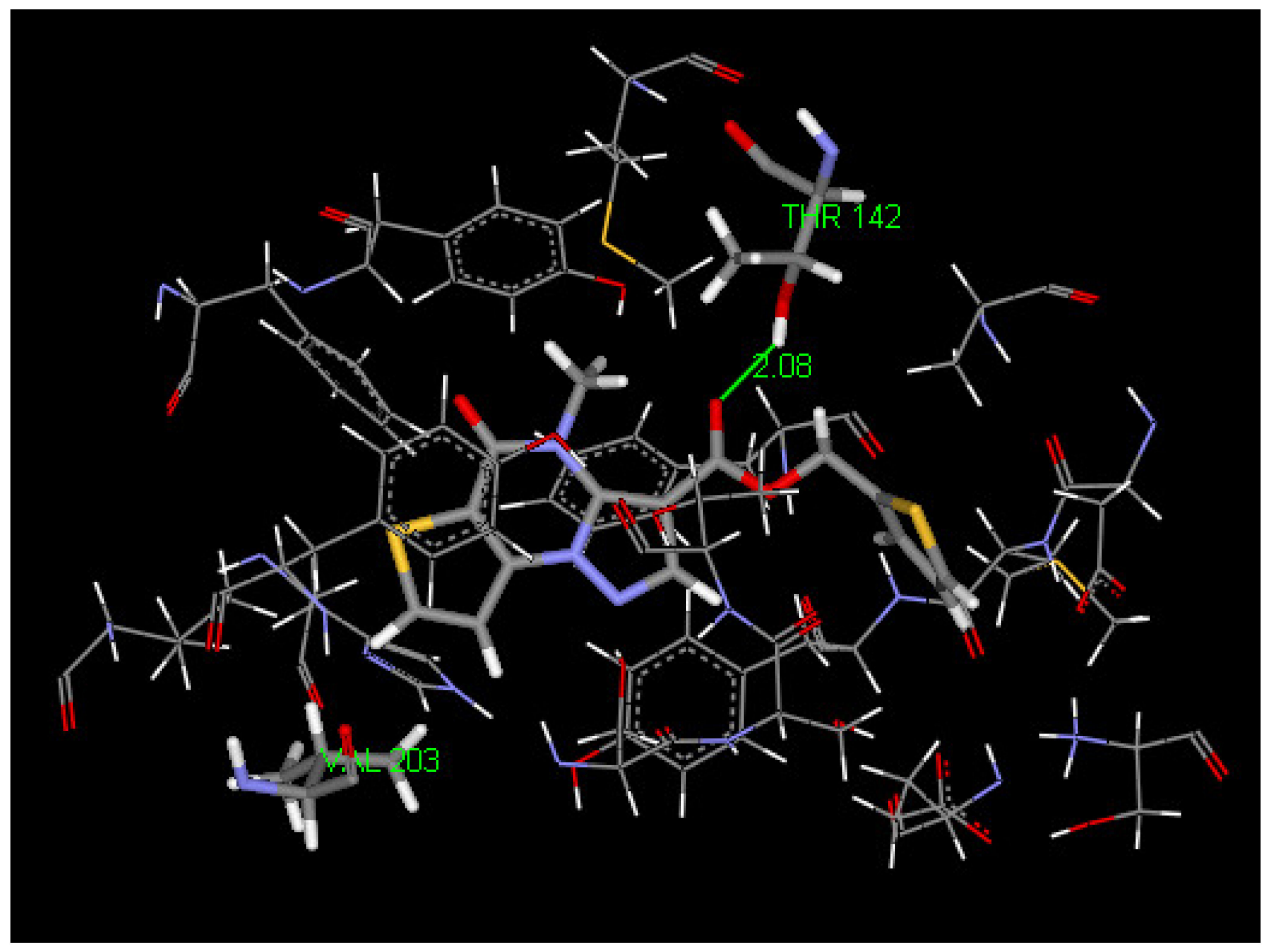
Hydrogen bond interaction of compound 22b in the last frame of dynamic simulation.

**Figure 8:**
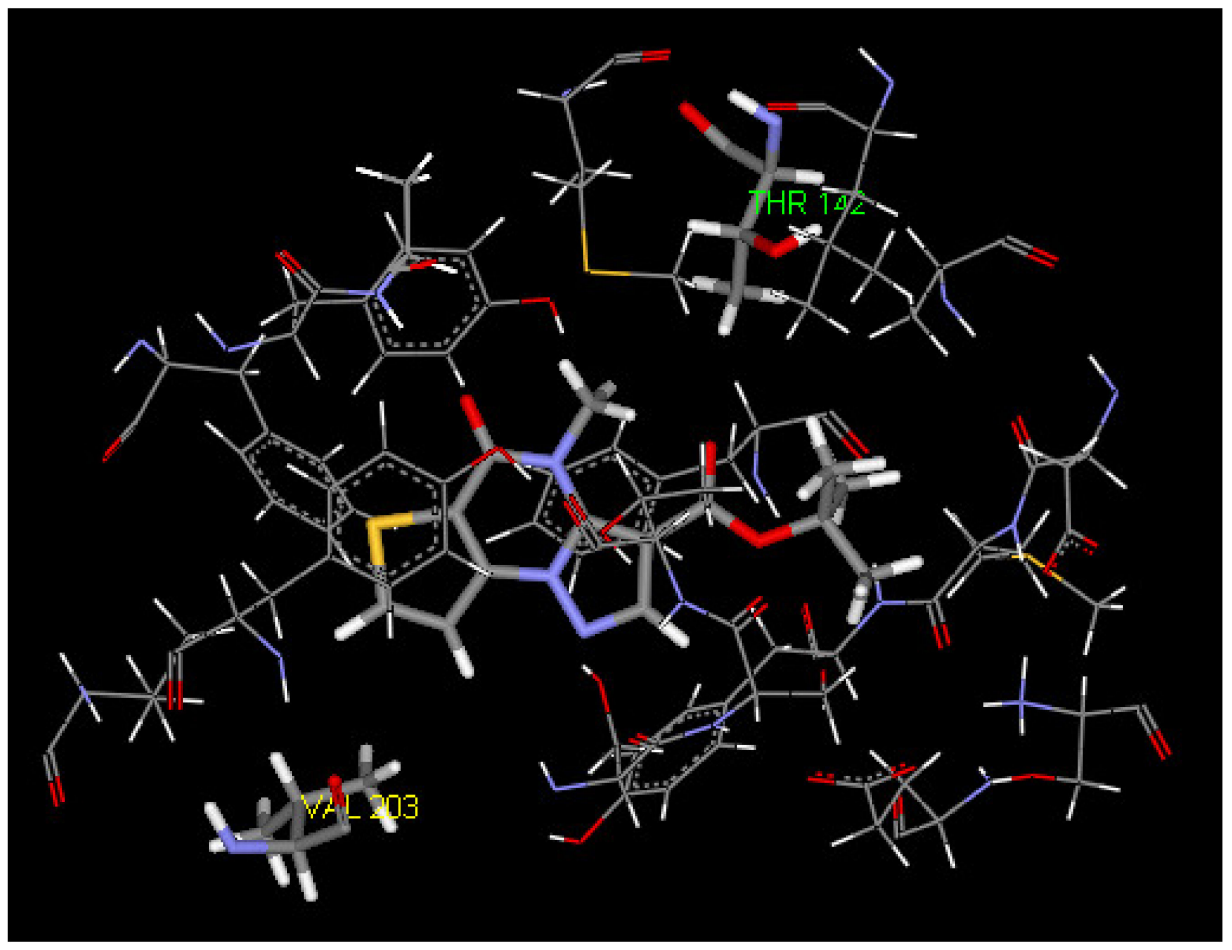
Hydrogen bond interaction of compound **22a** in the last frame of dynamic simulation.

Compounds **12c** and **22b** have in the position 3 an ester group which is able to engage a strong hydrogen bond interaction with γThr142 through the carbonyl moiety. Additionally, **22a** has an ester group in the 3 position but the steric hindrance of the *t*-butyloxycarbonyl fragment makes less probable the hydrogen bond interaction of the carbonyl group with the γThr142 residue; see Figure 6, Figure 7 and Figure 8.

These results are in accordance with our previously reported data [6,12], which evidenced the importance of the carbonyl group of the ester moiety to engage a strong hydrogen bond interaction with receptor protein. Compounds missing the ester group (**12d**–**f**) show a weak interaction with γThr142 in agreement with the low prediction percentage.

## 3. Experimental Section

### 3.1. Chemistry

**General procedure for the synthesis of compounds 3,4,6**-**8**. To a solution of 2 (1 mmol, 0.170 g) in DMF abs. and sodium acetate (1.3 mmol) was added 2-ethoxymethylenmalononitrile or ethyl-2-cyano-3-ethoxyacrylate, 3-(dimethylamino)- 2-(thien-2-carbonyl)acrylonitrile [15] (1.12 mmol) to obtain **3**, **4** and **6**. The solvent was AcOH when 3-oxo-2-(thien-3-yl)propionitrile and 3-oxo-2-(thien-2-yl)propionitrile [16] were used to obtain **7** and **8**. The reaction was refluxed for three hours, and after cooling, the addition of water and ice gave a precipitate that was filtered and purified by a suitable solvent

**5-Oxo-4,5-dihydropyrazolo[1,5-a]thieno[2,3-e]pyrimidine-3-carbonitrile (3)**. From **2** and 2-ethoxymethylenmalononitrile. Recrystallized by *i*-propanol, yield 60%, cream crystals, mp > 300 °C. TLC: toluene/ethyl acetate/methanol 8:2:1.5 *v*/*v*/*v* (Rf: 0.4); ^1^H-NMR (400 MHz, DMSO-d_6_) δ 13.47 (bs, 1H, NH, exch.); 8.28 (m, 2H, H-2 and H-7); 7.67 (d, 1H, H-8, *J =* 5.2 Hz). ^13^C-NMR (100 MHz, DMSO-d_6_) δ 163.70, 156.56, 148.45, 144.35, 138.87, 135.86, 129.09, 117.24, 115.45. Anal. C_9_H_4_N_4_OS (C, H, N).

**Ethyl 5-oxo-4,5-dihydropyrazolo[1,5-a]thieno[2,3-e]pyrimidine-3-carboxylate (4).** From **2** and ethyl 2-cyano-3-ethoxyacrylate. Recrystallized by ethanol, yield 58%, cream crystals, mp 215–216 °C. TLC: toluene/ethyl acetate/methanol 8:2:1.5 *v*/*v*/*v* (Rf: 0.5); ^1^H-NMR (400 MHz, DMSO-d_6_) δ 11.74 (bs, 1H, NH, exch.); 8.35 (d, 1H, H-7, *J =* 5.2 Hz); 8.17 (s, 1H, H-2); 7.68 (d, 1H, H-8, *J =* 5.2 Hz); 4.29 (q, 2H, CH_2_, *J =* 7.2 Hz); 1.29 (t, 3H, CH_3_, *J =* 7.2 Hz). ^1^H-NMR (400 MHz, CDCl_3_) δ 9.63 (bs, 1H, NH, exch.); 8.08 (s, 1H, H-2); 7.93 (d, 1H, H-7, *J =* 5.2 Hz); 7.67 (d, 1H, H-8, *J =* 5.2 Hz); 4.39 (q, 2H, CH_2_, *J =* 6.8 Hz); 1.41 (t, 3H, CH_3_, *J =* 6.8 Hz). ^13^C-NMR (100 MHz, DMSO-d_6_) δ 165.29, 161.50, 156.56, 144.35, 143.95, 135.87, 133.26, 129.05, 117.25, 60.94, 14.15. Anal. C_11_H_9_N_3_O_3_S (C, H, N).

**3-(Thiophene-2-carbonyl)pyrazolo[1,5-a]thieno[2,3-e]pyrimidin-5(4H)-one (6).** From **2** and 3-(dimethylamino)-2-(thien-2-carbonyl)acrylonitrile [15]. Recrystallized by ethanol, yield 95%, cream crystals, mp > 300 °C. TLC: toluene/ethyl acetate/methanol 8:2:1.5 *v*/*v*/*v* (Rf: 0.7); ^1^H-NMR (400 MHz, DMSO-d_6_) δ 11.59 (bs, 1H, NH, exch.); 8.63 (s, 1H, H-2); 8.37 (d, 1H, H-7, *J =* 5.2 Hz); 8.17 (s, 1H, H-5′); 8.05 (d, 1H, H-3′, *J =* 4.4 Hz); 7.74 (d, 1H, H-8, *J =* 5.2 Hz); 7.29 (m, 1H, H-4′). ^13^C-NMR (100 MHz, DMSO-d_6_) δ 157.90, 142.76, 142.25, 138.37, 134.86, 133.09, 129.39, 117.34. Anal. C_13_H_7_N_3_O_2_S_2_ (C, H, N).

**3-(Thiophene-3-yl)pyrazolo[1,5-a]thieno[2,3-e]pyrimidin-5(4H)-one (7).** From **2** and 3-oxo-2-(thien-3-yl)propionitrile [16]. Recrystallized by ethanol, yield 94%, cream crystals, mp 274–276 °C. TLC: toluene/ethyl acetate/methanol 8:2:1.5 *v*/*v*/*v* (Rf: 0.7); ^1^H-NMR (400 MHz, DMSO-d_6_) δ 12.06 (bs, 1H, NH, exch.); 8.29 (d, 1H, H-7, *J =* 5.2 Hz); 8.18 (s, 1H, H-2); 7.86 (s, 1H, H-2′); 7.67 (d, 1H, H-8, *J =* 5.2 Hz); 7.06 (m, 1H, H-4′); 7.52 (m, 1H, H-5′). ^13^C-NMR (100 MHz, DMSO-d_6_) δ 165.10, 144.35, 141.71, 139.60, 129.95, 128.30, 127.90, 124.75, 117.20. Anal. C_12_H_7_N_3_OS_2_ (C, H, N).

**3-(Thiophene-2-yl)pyrazolo[1,5-a]thieno[2,3-e]pyrimidin-5(4H)-one (8).** From **2** and 3-oxo-2-(thien-2-yl)propionitrile [16]. Recrystallized by ethanol, yield 89%, green light crystals, mp 248–250 °C. TLC: toluene/ethyl acetate/methanol 8:2:1.5 *v*/*v*/*v* (Rf: 0.7); ^1^H-NMR (400 MHz, DMSO-d_6_) δ 12.18 (bs, 1H, NH, exch.); 8.30 (d, 1H, H-7, *J =* 5.2 Hz); 8.00 (s, 1H, H-2); 7.67 (d, 1H, H-8, *J =* 5.2 Hz); 7.46 (d, 1H, H-5′, *J =* 4.8 Hz); 7.40 (d, 1H, H-3′, *J =* 2.8 Hz); 7.11 (dd, 1H, H-4′, *J_1_ =* 4.8 Hz, *J_2_ =* 4.0 Hz). ^13^C-NMR (100 MHz, DMSO-d_6_) δ 157.90, 141.71, 137.66, 128.40, 124.61, 117.19Anal. C_12_H_7_N_3_OS_2_ (C, H, N).

**5-Oxo-4,5-dihydropyrazolo[1,5-a]thieno[2,3-e]pyrimidine-3-carboxamide (5).** Compound **3** (0.23 mmol, 0.05 g) was suspended in H_2_SO_4_ conc., 1 mL and heated at 80 °C under stirring. After the starting material disappeared in TLC (toluene/ethyl acetate/methanol 8:2:1.5 *v*/*v*/*v*, as eluent, Rf: 0.2), the reaction was stopped; the addition of ice/water gave a precipitate which was filtered and purified by recrystallization with ethanol. Yield 92%, white crystals, mp > 300 °C. TLC: ^1^H-NMR (400 MHz, DMSO-d_6_) δ 10.75 (bs, 1H, NH, exch.); 8.33 (d, 1H, H-7, *J =* 4.8 Hz); 8.28 (s, 1H, H-2); 7.83 (bs, 1H, CONH, exch.); 7.68 (d, 1H, H-8, *J =* 4.8 Hz); 7.32 (bs, 1H, CONH, exch.).^13^C-NMR (100 MHz, DMSO-d_6_) δ 165.22, 163.70, 156.56, 150.55, 144.35, 143.91, 135.85, 132.06, 129.00. Anal. C_9_H_6_N_4_O_2_S (C, H, N).

**General procedure for the synthesis of compounds 9c,d.** Compounds **4**, **6** and **7** (0.6 mmol) were suspended in a mixture of POCl_3_ (5.5 mL) and PCl_5_ (0.91 mmol, 0.190 g) and refluxed for three hours. The evaporation to dryness to eliminate the excess of POCl_3_ gave a residue recuperated with ice/water filtered and purified with a suitable solvent, obtaining **9c**,**d**, starting from **4** and **6**, respectively. From compound **7**, the 5-chloro intermediate **9e** was not isolated but used as such, see below.

**Ethyl 5-chloropyrazolo[1,5-a]thieno[2,3-e]pyrimidine-3-carboxylate (9c).** From **5**. Recrystallized by ethanol, yield 90%, cream crystals, mp > 300 °C. TLC: toluene/ethyl acetate/methanol 8:2:1.5 *v*/*v*/*v* (Rf: 0.7); ^1^H-NMR (400 MHz, DMSO-d_6_) δ 8.61 (m, 2H, H-2 and H-7); 8.02 (d, 1H, H-8, *J =* 5.2 Hz); 4.29 (q, 2H, CH_2_, *J =* 7.2 Hz); 1.30 (t, 3H, CH_3_, *J =* 7.2 Hz). ^1^H-NMR (400 MHz, CDCl_3_) δ 8.55 (s, 1H, H-2); 8.08 (d, 1H, H-7, *J =* 5.6 Hz); 7.95 (d, 1H, H-8, *J =* 5.6 Hz); 4.45 (q, 2H, CH_2_, *J =* 7.2 Hz); 1.44 (t, 3H, CH_3_, *J =* 7.2 Hz). ^13^C-NMR (100 MHz, DMSO-d_6_) δ 161.80, 155.06, 145.85, 145.55, 130.55, 125.66, 126.70, 118.30, 60.95, 14.13. Anal. C_11_H_8_N_3_O_2_SCl (C, H, N).

**(5-Chloropyrazolo[1,5-a]thieno[2,3-e]pyrimidin-3-yl)(thiophen-2-yl)methanone (9d).** From **6**. Recrystallized by *i*-propanol, yield 95%, cream crystals, mp 186–188 °C. TLC: toluene/ethyl acetate/methanol 8:2:1.5 *v*/*v*/*v* (Rf: 0.5); ^1^H-NMR (400 MHz, DMSO-d_6_) δ 8.80 (s, 1H, H-2); 8.65 (d, 1H, H-7, *J =* 5.2 Hz); 8.14 (s, 1H, H-5′); 8.07 (m, 2H, H-8 and H-3′); 7.30 (m, 1H, H-4′). ^13^C-NMR (100 MHz, DMSO-d_6_) δ 173.70, 155.06, 145.88, 145.53, 144.35, 135.81, 133.72, 130.50, 127.06, 126.74, 125.66, 126.70, 118.30. Anal. C_13_H_6_N_3_OS_2_Cl (C, H, N).

**General procedure for the synthesis of compounds 10c,d.** Compounds **9c**,**d** (0.4 mmol) was dissolved in a mixture of CH_2_Cl_2_/EtOH (7.5 mL/15 mL) and NaBH_4_ (3.6 mmol, 0.136 g) was added in small portions. The reaction was maintained at room temperature for 40 min, and then the evaporation to dryness of the solvent gave a residue which was recovered with water, filtered and purified with a suitable solvent, obtaining **10c**,**d**, respectively.

**Ethyl 4,5-dihydropyrazolo[1,5-a]thieno[2,3-e]pyrimidine-3-carboxylate (10c).** From **9c**. Recrystallized by water, yield 45%, cream crystals, mp 126–128 °C. TLC: toluene/ethyl acetate/methanol 8:2:1.5 *v*/*v*/*v* (Rf: 0.5); ^1^H-NMR (400 MHz, DMSO-d_6_) δ 7.57 (m, 2H, H-2 and H-7); 7.18 (d, 1H, H-8, *J =* 4.4 Hz); 7.05 (s, 1H, NH, exch.); 4.7 (s, 2H, NCH_2_); 4.17 (q, 2H, CH_2_, *J =* 7.2 Hz); 1.25 (t, 3H, CH_3_, *J =* 7.2 Hz). ^1^H-NMR (400 MHz, CDCl_3_) δ 8.55 (s, 1H, H-2); 8.08 (d, 1H, H-7, *J =* 5.6 Hz); 7.95 (d, 1H, H-8, *J =* 5.6 Hz); 4.70 (s, 2H, NCH_2_); 4.18 (q, 2H, CH_2_, *J =* 6.8 Hz); 1.25 (t, 3H, CH_3_, *J =* 6.8 Hz). ^13^C-NMR (100 MHz, DMSO-d_6_) δ 163.29, 140.97, 125.82, 116.47, 59.18, 41.96, 15.02. Anal. C_11_H_11_N_3_O_2_S (C, H, N).

**4,5-Dihydropyrazolo[1,5-a]thieno[2,3-e]pyrimidin-3-yl(thiophen-2-yl)methanone (10d).** From **9d**. Recrystallized by ethanol, yield 40%, cream crystals, mp 186–188 °C. TLC: toluene/ethyl acetate/methanol 8:2:1.5 *v*/*v*/*v* (Rf: 0.3); ^1^H-NMR (400 MHz, DMSO-d_6_) δ 8.11 (s, 1H, H-2); 7.97 (s, 1H, H-5′); 7.91 (d, 1H, H-7, *J =* 4.8 Hz); 7.78 (s, 1H, NH, exch.); 7.61 (d, 1H, H-8 *J =* 4.8 Hz); 7.22 (m, 2H, H-3′ and H-4′); 4.79 (s, 2H, NCH_2_). ^13^C-NMR (100 MHz, DMSO-d_6_) δ 172.98, 158.01, 144.82, 144.35, 137.85, 135.71, 133.57, 129.06, 127.74, 123.66, 119.30, 51.55. Anal. C_13_H_9_N_3_OS_2_ (C, H, N).

**General procedure for the synthesis of compounds 11c,d.** Compounds **10c,d** (0.4 mmol) was dissolved in toluene (15 mL), and Pd/C as catalyst was added. The reaction was refluxed for 3–5 h and filtered off the catalyst. The evaporation to dryness of the solution yielded a residue recovered with water, filtered and purified with a suitable solvent, obtaining **11c** and **11d**, respectively.

**Ethyl pyrazolo[1,5-a]thieno[2,3-e]pyrimidine-3-carboxylate (11c).** From **10c**. Recrystallized by ethanol yield 50%, white crystals, mp 159–160 °C. TLC: toluene/ethyl acetate 8:2 *v*/*v* (Rf: 0.1); ^1^H-NMR (400 MHz, DMSO-d_6_) δ 9.42 (s, 1H, H-5); 8.60 (s, 1H, H-2); 8.57 (d, 1H, H-7, *J =* 5.2 Hz); 7.98 (d, 1H, H-8, *J =* 5.2 Hz); 4.31 (q, 2H, CH_2_, *J =* 6.8 Hz); 1.32 (t, 3H, CH_3_, *J =* 6.8 Hz). ^13^C-NMR (100 MHz, DMSO-d_6_) δ 162.34, 148.66, 145.99, 145.25, 141.82, 140.02, 123.02, 115.95, 103.38, 60.08, 14.92. Anal. C_11_H_9_N_3_O_2_S (C, H, N).

**Pyrazolo[1,5-a]thieno[2,3-e]pyrimidin-3-yl(thiophen-2-yl)methanone (11d).** From **10d**. Recrystallized by *i*-propanol, yield 68%, white crystals, mp 73–75 °C. TLC: toluene/ethyl acetate/methanol 8:2:1.5 *v*/*v*/*v* (Rf: 0.2); ^1^H-NMR (400 MHz, DMSO-d_6_) δ 9.42 (s, 1H, H-5); 8.74 (s, 1H, H-2); 8.59 (d, 1H, H-7, *J =* 4.8 Hz); 8.23 (d, 1H, H-5′ *J =* 2.4 Hz); 8.03 (m, 2H, H-3′ and H-8); 7.28 (m, 1H, H-4′). ^13^C-NMR (100 MHz, DMSO-d_6_) δ 173.08, 149.01, 145.82, 144.85, 144.35, 135.81, 133.43, 130.57, 129.06, 126.74, 125.60, 119.10. Anal. C_13_H_7_N_3_OS_2_ (C, H, N).

**3-(Thiophen-3-yl)pyrazolo[1,5-a]thieno[2,3-e]pyrimidine (11e).** Compound **7**, 3-(thiophene-3-yl)pyrazolo[1,5-a]thieno[2,3-e]pyrimidin-5(4H)-one (0.6 mmol), was suspended in a mixture of POCl_3_ (5.5 mL) and PCl_5_ (0.91 mmol, 0.190 g) and refluxed for three hours. The evaporation to dryness to eliminate the excess of POCl_3_ gave the corresponding 5-chloro derivative (**9e**), not isolated but used as such for the next reduction step through a CTH (catalytic transfer hydrogenation). Thus, this intermediate suspended in EtOH (20 mL) was added of ammonium formate (4.08 mmol, 0.275 g) and 10% Pd/C as catalyst. The reaction was maintained at reflux temperature for several hours, during which it was possible to evidence, by TLC, the formation of the 4,5-dihydro derivative in a mixture with the final 4,5-dehydro compound **11e**. When the reaction finished, the catalyst was filtered off, the solution evaporated to dryness, and the residue recovered with water. Recrystallized by ethanol 80%, yield 90%, cream crystals, mp 157–160 °C. TLC: toluene/ethyl acetate 8:2 *v*/*v* (Rf: 0.5); ^1^H-NMR (400 MHz, DMSO-d_6_) δ 9.24 (s, 1H, H-5); 8.65 (s, 1H, H-2); 8.45 (d, 1H, H-7, *J =* 5.2 Hz); 8.05 (s, 1H, H-2′); 7.95 (d, 1H, H-8, *J =* 5.2 Hz); 7.86 (d, 1H, H-5′, *J =* 4.8 Hz); 7.65 (m, 1H, H-4′). ^13^C-NMR (100 MHz, DMSO-d_6_) δ 145.56, 141.21, 138.47, 126.77, 119.61, 115.88. Anal. C_12_H_7_N_3_S_2_ (C, H, N).

**General procedure for the synthesis of compounds 12a,c–f and 13b,e,f.** A solution of DMF abs. (5 mL), compounds **3**–**8** (0.40 mmol) and K_2_CO_3_ anhydrous (0.80 mmol) was maintained for 15 min at room temperature. After this time, methyl iodide (0.80 mmol) was added and enhanced temperature to 80 °C. After one hour and monitoring the reaction by TLC, adding water gave a precipitate, filtered and purified by a suitable solvent. In the case of compounds **3**, **4** and **6**, only 4-N-CH_3_ derivatives were formed (**12a**,**c**,**d**). From compound **4**, only the 5-methoxyderivative **13b** was recovered, while if the reaction is performed at reflux temperature, only the 4-methyl derivative **12b** was obtained. From **7** and **8**, a mixture of two products was recovered at the end of alkylation. The chromatographic separation permits isolating the 4-NCH_3_ (**12e,f**) and the 5-OCH_3_ derivatives (**13e,f**).

**4-Methyl-5-oxo-4,5-dihydropyrazolo[1,5-a]thieno[2,3-e]pyrimidine-3-carbonitrile (12a).** From **3**. Recrystallized by ethanol, yield 89%, cream crystals, mp 224–226 °C. TLC: toluene/ethyl acetate/methanol 8:2:1 *v*/*v*/*v* (Rf: 0.6); ^1^H-NMR (400 MHz, DMSO-d_6_) δ 8.44 (s, 1H, H-2); 8.36 (d, 1H, H-7, *J =* 4.0 Hz); 7.71 29/03/2023 (d, 1H, H-8, *J =* 4.0 Hz); 3.73 (s, 3H, NCH_3_). ^13^C-NMR (100 MHz, DMSO-d_6_) δ 163.76, 156.51, 148.40, 144.37, 144.30, 135.86, 129.07, 115.41, 106.58, 36.50. Anal. C_10_H_6_N_4_OS (C, H, N).

**4-Methyl-5-oxo-4,5-dihydropyrazolo[1,5-a]thieno[2,3-e]pyrimidine-3-carboxamide (12b).** From **5** at reflux temperature. Recrystallized by ethanol, yield 93%, cream crystals, mp > 300 °C. TLC: dichlorometane/methanol 9:1 *v*/*v* (Rf: 0.3); ^1^H-NMR (400 MHz, DMSO-d_6_) δ 8.28 (d, 1H, H-7, *J =* 4.8 Hz); 8.11 (s, 1H, H-2); 7.86 (bs, 1H, CONH, exch.); 7.65 (d, 1H, H-8, *J =* 4.8 Hz); 7.32 (bs, 1H, CONH, exch.); 3.73 (s, 3H, NCH_3_). ^13^C-NMR (100 MHz, DMSO-d_6_) δ 165.30, 163.76, 156.51, 150.40, 145.67, 144.31, 135.86, 132.05, 129.08, 36.69. Anal. C_10_H_8_N_4_O_2_S (C, H, N). The treatment of compound **12a** with sulfuric acid at 60 °C and the subsequent addition of ice/water gave the precipitate, **12b** recovered by filtration.

**Ethyl 4-methyl-5-oxo-4,5-dihydropyrazolo[1,5-a]thieno[2,3-e]pyrimidine-3-carboxylate (12c).** From **4**. Recrystallized by ethanol, yield 75%, cream crystals, mp 173–174 °C. TLC: toluene/ethyl acetate/methanol 8:2:1.5 *v*/*v*/*v* (Rf: 0.5); ^1^H-NMR (400 MHz, CDCl_3_) δ 8.19 (s, 1H, H-2); 7.86 (d, 1H, H-7, *J =* 5.2 Hz); 7.64 (d, 1H, H-8, *J =* 5.2 Hz); 4.34 (q, 2H, CH_2_, *J =* 7.2 Hz); 4.04 (s, 3H, NCH_3_); 1.40 (t, 3H, CH_3_, *J =* 7.2 Hz). ^13^C-NMR (100 MHz, DMSO-d_6_) δ 161.78, 156.10, 145.24, 142.00, 141.69, 138.12, 117.25, 116.95, 98.60, 60.77, 33.57, 14.59. Anal. C_12_H_11_N_3_O_3_S (C, H, N).

**3-(Thiophene-2-carbonyl)-4-methylpyrazolo[1,5-a]thieno[2,3-e]pyrimidin-5(4H)-one (12d).** From **6**. Recrystallized by ethanol, yield 95%, cream crystals, mp 183–186 °C. TLC: toluene/ethyl acetate/methanol 8:2:1.5 *v*/*v*/*v* (Rf: 0.5); ^1^H-NMR (400 MHz, DMSO-d_6_) δ 8.37 (d, 1H, H-7, *J =* 5.2 Hz); 8.33 (s, 1H, H-2); 8.10 (d, 1H, H-5′ *J =* 4.8 Hz); 7.82 (d, 1H, H-3′, *J =* 3.6 Hz); 7.74 (d, 1H, H-8, *J =* 5.2 Hz); 7.30 (m, 1H, H-4′); 3.56 (s, 3H NCH_3_). ^13^C-NMR (100 MHz, DMSO-d_6_) δ 179.37, 156.18, 145.24, 144.91, 141.71, 138.40, 135.92, 135.62, 129.12, 117.39, 106.50, 33.07. Anal. C_14_H_9_N_3_O_2_S_2_ (C, H, N).

**3-(Thiophene-3-yl)-4-methylpyrazolo[1,5-a]thieno[2,3-e]pyrimidin-5(4H)-one (12e).** From **7**, after chromatographic separation, second eluting band (toluene/ethyl acetate/methanol 8:2:1.5 *v*/*v*/*v* as eluent, Rf: 0.5), yield 35%, cream crystals, mp 109–111 °C. ^1^H-NMR (400 MHz, DMSO-d_6_) δ 8.29 (d, 1H, H-7, *J =* 4.8 Hz); 7.85 (s, 1H, H-2); 7.68 (d, 1H, H-8, *J =* 4.8 Hz); 7.63–7.59 (m, 2H, H-2′ and H-4′); 7.25 (m, 1H, H-5′); 3.40 (s, 3H, NCH_3_). ^1^H-NMR (400 MHz, CDCl_3_) δ 7.83 (d, 1H, H-7, *J =* 5.2 Hz); 7.71 (s, 1H, H-2); 7.65 (d, 1H, H-8, *J =* 5.2 Hz); 7.40 (m, 1H, H-2′); 7.26 (m, 1H, H-4′); 7.11 (d, 1H, H-5′, *J =* 4.4 Hz); 3.41 (s, 3H, NCH_3_). ^13^C-NMR (100 MHz, DMSO-d_6_) δ 157.94, 144.97, 140.00, 138.10, 137.90, 130.86, 125.99, 114.34, 114.23, 106.12, 20.54. Anal. C_13_H_9_N_3_OS_2_ (C, H, N).

**3-(Thiophene-2-yl)-4-methylpyrazolo[1,5-a]thieno[2,3-e]pyrimidin-5(4H)-one (12f).** From **8** after chromatographic separation, second eluting band (toluene/ethyl acetate/methanol 8:2:1.5 *v*/*v*/*v* as eluent, Rf: 0.5), yield 36%, yellow light crystals, mp 140–141 °C. ^1^H-NMR (400 MHz, DMSO-d_6_) δ 8.30 (d, 1H, H-7, *J =* 5.2 Hz); 7.90 (s, 1H, H-2); 7.68 (d, 1H, H-8, *J =* 5.2 Hz); 7.62 (d, 1H, H-5′, *J =* 5.2 Hz); 7.18 (m, 1H, H-3′); 7.13 (dd, 1H, H-4′, *J_1_ =* 4.8 Hz, *J_2_ =* 3.6 Hz); 3.31 (s, 3H, NCH_3_). ^13^C-NMR (100 MHz, DMSO-d_6_) δ 165.46, 154.88, 145.71, 137.76, 135.14, 131.79, 129.06, 117.48, 114.01, 56.89. Anal. C_13_H_9_N_3_OS_2_ (C, H, N).

**5-Methoxypyrazolo[1,5-a]thieno[2,3-e]pyrimidine-3-carboxamide (13b).** From **5** at room temperature. Recrystallized by ethanol, yield 75%, cream crystals, mp 269–270 °C. TLC: dichlorometane/methanol 8:2 *v*/*v* (Rf: 0.6); ^1^H-NMR (400 MHz, DMSO-d_6_) δ 8.42 (d, 1H, H-7, *J =* 4.8 Hz); 8.36 (s, 1H, H-2); 7.90 (d, 1H, H-8, *J =* 4.8 Hz); 7.49 (bs, 1H, CONH, exch.); 7.34 (bs, 1H, CONH, exch.); 4.22 (s, 3H, OCH_3_). ^13^C-NMR (100 MHz, DMSO-d_6_) δ 164.40, 162.26, 145.87, 145.50, 130.56, 125.68, 114.45, 107.32, 53.79. Anal. C_10_H_8_N_4_O_2_S (C, H, N).

**5-Methoxy-3-(thiophene-3-yl)pyrazolo[1,5-a]thieno[2,3-e]pyrimidine (13e).** From **7**, after chromatographic separation, first eluting band (toluene/ethyl acetate/methanol 8:2:1.5 *v*/*v*/*v* as eluent, Rf: 0.8), yield 35%, cream crystals, mp 127–130 °C. ^1^H-NMR (400 MHz, DMSO-d_6_) δ 8.49 (s, 1H, H-2); 8.38 (d, 1H, H-7, *J =* 5.2 Hz); 7.98 (m, 1H, H-2′); 7.85 (d, 1H, H-8, *J =* 5.2 Hz); 7.81 (d, 1H, H-5′, *J =* 4.8 Hz); 7.62 (m, 1H, H-4′); 4.20 (s, 3H, OCH_3_). ^13^C-NMR (100 MHz, DMSO-d_6_) δ 162.20, 144.86, 139.60, 133.47, 128.37, 128.20, 126.71, 125.60, 124.75, 114.56, 107.90, 53.75. Anal. C_13_H_9_N_3_OS_2_ (C, H, N).

**5-Methoxy-3-(thiophene-2-yl)pyrazolo[1,5-a]thieno[2,3-e]pyrimidine (13f).** From **8** after chromatographic separation, first eluting band (toluene/ethyl acetate/methanol 8:2:1.5 *v*/*v*/*v* as eluent, Rf: 0.8), yield 24%, yellow light crystals, mp 118–120 °C. ^1^H-NMR (400 MHz, DMSO-d_6_) δ 8.43 (s, 1H, H-2); 8.39 (d, 1H, H-7, *J =* 5.2 Hz); 7.85 (d, 1H, H-8, *J =* 5.2 Hz); 7.57 (m, 1H, H-5′); 7.42 (d, 1H, H-3′, *J =* 4.8 Hz); 7.11 (m, 1H, H-4′); 4.20 (s, 3H, OCH_3_). ^13^C-NMR (100 MHz, DMSO-d_6_) δ 162.20, 144.86, 138.20, 133.65, 130.57, 128.67, 128.00, 126.74, 125.60, 114.56, 107.90, 53.50. Anal. C_13_H_9_N_3_OS_2_ (C, H, N).

**5-Oxo-4,5-dihydropyrazolo[1,5-a]thieno[2,3-e]pyrimidine-3-carboxylic acid (14).** To a suspension of **4** (0.40 mmol) in NaOH 10% solution (10 mL), was added 0.5 mL of methoxyethanol to favour the solubilization. The reaction was refluxed for 1.30 h, then ice/water and HCl 6N until pH 1. The precipitate formed was filtered, washed with water and purified by a suitable solvent. Recrystallized by ethanol, yield 78%, cream crystals, mp 282–284 °C. TLC: toluene/ethyl acetate/acetic acid 8:2:2 *v*/*v*/*v* (Rf: 0.5); ^1^H-NMR (400 MHz, DMSO-d_6_) δ 12.70 (bs, 1H, OH, exch.); 11.37 (bs, 1H, NH, exch.); 8.34 (d, 1H, H-7, *J =* 4.4 Hz); 8.13 (s, 1H, H-2); 7.68 (d, 1H, H-8, *J =* 4.4 Hz). ^13^C-NMR (100 MHz, DMSO-d_6_) δ 165.25, 164.96, 156.50, 144.35, 143.97, 135.87, 133.20, 129.04, 117.65. Anal. C_9_H_5_N_3_O_3_S (C, H, N).

**5-Oxo-4,5-dihydropyrazolo[1,5-a]thieno[2,3-e]pyrimidine (15).** The acid **14** (0.50 mmol) was suspended in 10 mL of HCl conc. and maintained at reflux temperature for 5 h. Adding ice/water gave a residue filtered and purified by recrystallization with ethanol. Recrystallized by ethanol, yield 65%, cream crystals, mp 290–291 °C. TLC: toluene/ethyl acetate/methanol 8:2:1.5 *v*/*v*/*v* (Rf: 0.7); ^1^H-NMR (400 MHz, DMSO-d_6_) δ 12.30 (bs, 1H, NH, exch.); 8.26 (s, 1H, H-7); 7.74 (s, 1H, H-2); 7.64 (s, 1H, H-8); 5.93 (s, 1H, H-3). ^13^C-NMR (100 MHz, DMSO-d_6_) δ 164.76, 156.26, 148.45, 144.37, 135.85, 133.21, 129.09, 114.15. Anal. C_8_H_5_N_3_OS (C, H, N).

**4-Methyl-5-oxo-4,5-dihydropyrazolo[1,5-a]thieno[2,3-e]pyrimidine (16).** The ester **12c** (0.50 mmol) was suspended in 10 mL of NaOH 10% solution and maintained at 80 °C until the starting material disappeared. The extraction with ethyl acetate and the next usual work up gave a residue which was filtered and purified by recrystallization with ethanol. Yield 71%, cream crystals, mp 157–159 °C. TLC: toluene/ethyl acetate/acetic acid 8:2:1 *v*/*v*/*v* (Rf: 0.8); ^1^H-NMR (400 MHz, DMSO-d_6_) δ 8.18 (d, 1H, H-7, *J =* 5.6 Hz); 7.83 (d, 1H, H-2 *J =* 2.0 Hz); 7.62 (d, 1H, H-8, *J =* 5.6 Hz); 6.22 (d, 1H, H-3 *J =* 2.0 Hz); 3.86 (s, 3H, N-CH_3_). ^1^H-NMR (400 MHz, CDCl_3_) δ 7.82 (d, 1H, H-7, *J =* 5.6 Hz); 7.87 (d, 1H, H-2 *J =* 2.0 Hz); 7.64 (d, 1H, H-8, *J =* 5.6 Hz); 5.96 (d, 1H, H-3 *J =* 2.0 Hz); 3.63 (s, 3H, N-CH_3_). ^13^C-NMR (100 MHz, DMSO-d_6_) δ 163.71, 156.56, 148.49, 144.30, 135.80, 133.27, 129.09, 114.20, 37.05. Anal. C_9_H_7_N_3_OS (C, H, N).

**General procedure for the synthesis of compounds 17 and 19.** A suspension of **12b** or **13b** (0.32 mmol) in H_2_SO_4_ conc. (8 mL) was stirred until a solution was obtained and then cooled at 0 °C; to this solution, sodium nitrite (0.22g, 3.2 mmol/5 mL of water) was slowly added and the green suspension was maintained for 3 h at 0 °C. The suspension was made alkaline and extracted with ethyl acetate. After the standard work-up, the evaporation of the organic layer gave a green residue that was purified and characterized.

**4-Methyl-3-nitrosopyrazolo[1,5-a]thieno[2,3-e]pyrimidin-5(4H)-one (17).** From **12b**. Recrystallized by ethanol, yield 50%, green crystals, mp 240–242 °C. TLC: toluene/ethyl acetate/acetic acid 8:2:1 *v*/*v*/*v* (Rf: 0.9); ^1^H-NMR (400 MHz, DMSO-d_6_) δ 8.44 (d, 1H, H-7, *J =* 5.6 Hz); 7.74 (d, 1H, H-8, *J =* 5.6 Hz); 7.72 (s, 1H, H-2); 4.21 (s, 3H, NCH_3_). ^13^C-NMR (100 MHz, DMSO-d_6_) δ 163.70, 156.59, 148.49, 144.30, 134.99, 133.25, 129.01, 104.05, 36.99. ESI-HRMS (m/z) calculated for [M+H]^+^ ion species C_9_H_6_N_4_O_2_S = 235,0295; found: 235,0284. Anal. C_9_H_6_N_4_O_2_S (C, H, N).

**5-Methoxy-3-nitrosopyrazolo[1,5-a]thieno[2,3-e]pyrimidine (19).** From **13b**. Recrystallized by ethanol, yield 48%, green crystals, mp 218–220 °C. TLC: toluene/ethyl acetate/acetic acid 8:2:1 *v*/*v*/*v* (Rf: 0.9); ^1^H-NMR (400 MHz, DMSO-d_6_) δ 8.86 (s, 1H, H-2); 8.54 (d, 1H, H-7, *J =* 5.6 Hz); 7.94 (d, 1H, H-8, *J =* 5.6 Hz); 4.23 (s, 3H, OCH_3_). ^13^C-NMR (100 MHz, DMSO-d_6_) δ 163.65, 156.60, 148.49, 144.30, 134.99, 133.20, 128.99, 104.10, 55.60. ESI-HRMS (m/z) calculated for [M+H]^+^ ion species C_9_H_6_N_4_O_2_S = 235,0294; found: 235,0284. Anal. C_9_H_6_N_4_O_2_S (C, H, N).

**5-Methoxy-3-nitrosopyrazolo[1,5-a]thieno[2,3-e]pyrimidin-3-carboxylic acid (18).** From **13b**, after acidification of the alkaline solution. The carboxylic acid was recrystallized by ethanol, yield 30%, white crystals, mp 218–220 °C. TLC: toluene/ethyl acetate/acetic acid 8:2:1 *v*/*v*/*v* (Rf: 0.5); ^1^H-NMR (400 MHz, DMSO-d_6_) δ 12.24 (bs, 1H, OH, exch.); 8.44 (d, 1H, H-7, *J =* 4.8 Hz); 8.38 (s, 1H, H-2); 7.88 (d, 1H, H-8, *J =* 4.8 Hz); 4.17 (s, 3H, OCH_3_). ^13^C-NMR (100 MHz, DMSO-d_6_) δ 169.35, 162.20, 145.80, 145.59, 130.50, 126.70, 125.69, 114.50, 107.10, 53.70. Anal. C_10_H_7_N_3_O_3_S (C, H, N).

**4-Methyl-5-oxo-4,5-dihydropyrazolo[1,5-a]thieno[2,3-e]pyrimidin-3-carbaldehyde (20).** A suspension of **16** (150 mg, 0.73 mmol) in glacial acetic acid (6 mL) was added of hexamethylenetetramine (HTMA, 0.36 g) and maintained at reflux temperature for 10 h. After disappearing the starting material, evaluated by TLC (CHX/EtOAc 1:5, *v*/*v* as eluent, Rf: 0.8), the addition of ice gave a precipitate that was recovered by filtration. Yield 85%, white crystals, mp 222–224 °C. ^1^H-NMR (400 MHz, CDCl_3_) δ 10.02 (s, 1H, CHO); 8.27 (s, 1H, H-2); 7.91 (d, 1H, H-7, *J =* 5.2 Hz); 7.88 (d, 1H, H-8, *J =* 5.2 Hz); 4.03 (s, 3H, OCH_3_). ^13^C-NMR (100 MHz, DMSO-d_6_) δ 170.35, 163.20, 156.25, 145.80, 144.59, 135.84, 133.50, 129.50, 107.15, 37.50. Anal. C_10_H_7_N_3_O_2_S (C, H, N).

**4-Methyl-5-oxo-4,5-dihydropyrazolo[1,5-a]thieno[2,3-e]pyrimidin-3-carboxylic acid (21).** The aldehyde **20** (150 mg, 0.73 mmol) was suspended in acetone and water (5 mL/5 mL) and a solution of potassium permanganate (1.1 mmol) in water was added after the suspension was made alkaline with sodium hydroxide 10%. The reaction was heated for 8 h, and after cooling and elimination of the manganese dioxide by filtration, the alkaline aqueous phase was extracted to eliminate the starting material not reacting. The next acidification of the aqueous phase gave the corresponding carboxylic acid that was recovered by extraction. Yield 60%, white crystals, mp 220–223 °C. TLC: CHX/EtOAc 1:5, *v*/*v* (Rf: 0.2); ^1^H-NMR (400 MHz, DMSO-d_6_) δ 12.68 (bs, 1H, OH, exch.); 8.32 (d, 1H, H-7, *J =* 4.0 Hz); 8.21 (s, 1H, H-2); 7.67 (d, 1H, H-8, *J =* 4.0 Hz); 3.87 (s, 3H, NCH_3_). ^13^C-NMR (100 MHz, DMSO-d_6_) δ 169.10, 162.40, 145.85, 145.59, 130.50, 126.55, 125.80, 115.50, 107.10, 37.70. Anal. C_10_H_7_N_3_O_3_S (C, H, N).

**General procedure for the synthesis of compounds 22a,b.** The carboxylic acid **21** (0.5 mmol) was transformed into the corresponding 3-carbonyl chloride by reaction with excess SOCl_2_ in anhydrous conditions. After the standard work-up, the residue was suspended in dichloromethane (6 mL), and the suitable alcohol (excess 0.15 mL) was added; TLC monitored the reaction until the disappearance of the starting material. Then, the final solution was evaporated to dryness, and the residue recuperated with isopropyl ether and recrystallized.

***tert*-Butyl 4-methyl-5-oxo-4,5-dihydropyrazolo[1,5-a]thieno[2,3-e]pyrimidine-3-carboxylate (22a).** From **21** and *tert*-butanol, white crystals recrystallized by 80% ethanol, yield 25%; mp > 300 °C. TLC: toluene/ethyl acetate/methanol 8:2:1.5 *v*/*v*/*v* (Rf: 0.5); ^1^H-NMR (400 MHz, CDCl_3_) δ 8.10 (s, 1H, H-2); 7.85 (d, 1H, H-7, *J =* 4.8 Hz); 7.63 (d, 1H, H-8, *J =* 4.8 Hz); 4.01 (s, 3H, NCH_3_); 1.50 (s, 9H, (CH_3_)_3_). ^13^C-NMR (100 MHz, DMSO-d_6_) δ 161.78, 156.10, 145.24, 142.00, 141.69, 138.12, 117.25, 116.95, 98.60, 33.57, 14.59. Anal. C_14_H_15_N_3_O_3_S (C, H, N).

**Thiophen-2-yl-methyl 4-methyl-5-oxo-4,5-dihydropyrazolo[1,5-a]thieno[2,3-e]pyrimidine-3-carboxylate (22b).** From **21** and 2-thiophenmethanol, white crystals recrystallized by 80% ethanol, yield 30%; mp 190–195 °C. TLC: toluene/ethyl acetate/methanol 8:2:1.5 *v*/*v*/*v* (Rf: 0.6); ^1^H-NMR (400 MHz, CDCl_3_) δ 8.19 (s, 1H, H-2); 7.85 (d, 1H, H-7, *J =* 5.2 Hz); 7.63 (d, 1H, H-8, *J =* 5.2 Hz); 7.35 (d, 1H, H-5 Thiophene, *J =* 5.2 Hz); 7.18 (s, 1H, H-3 Thiophene); 7.03 (d, 1H, H-4 Thiophene, *J_1_ =* 4.8 Hz); 5.47 (s, 2H, OCH_2_); 4.03 (s, 3H, NCH_3_). ^13^C-NMR (100 MHz, CDCl_3_) δ 145.60, 135.92, 128.25, 126.89, 117.02, 60.81, 33.87. Anal. C_15_H_11_N_3_O_3_S_2_ (C, H, N).

**Pyrazolo[1,5-a]thieno[2,3-e]pyrimidine-3-carboxylic acid (23).** Compound **11c** (0.40 mmol) was treated with 10% NaOH solution and maintained at 80 °C until the starting material disappeared in TLC (toluene/ethyl acetate/acetic acid 8:2:2 *v*/*v*/*v* as eluent). However, the reaction gave many compounds, and the fast-eluted band’s fluorescent spot was recovered by purification with a chromatography column (toluene/ethyl acetate/acetic acid 8:2:2 *v*/*v*/*v* as eluent, Rf: 0.5). Yield 15%, white crystals, mp 246–247 °C. ^1^H-NMR (400 MHz, DMSO-d_6_) δ 12.05 (bs, 1H, OH, exch.); 9.34 (s, 1H, H-5); 8.55 (s, 1H, H-2); 8.50 (d, 1H, H-7, *J =* 5.2 Hz); 7.95 (d, 1H, H-8, *J =* 5.2 Hz). ^13^C-NMR (100 MHz, CDCl_3_) δ 145.60, 135.93, 128.25, 126.89, 117.02, 60.81, 33.87. Anal. C_9_H_5_N_3_O_2_S (C, H, N).

**Pyrazolo[1,5-a]thieno[2,3-e]pyrimidine (24).** Compound **11c** (0.40 mmol) were treated with HCl/CH_3_COOH solution and maintained at 70 °C until the starting material disappeared in TLC (toluene/ethyl acetate/acetic acid 8:2:2 *v*/*v*/*v* as eluent, Rf: 0.6). The reaction from 11c, monitored by TLC evidenced the formation of 3-carboxylic acid (23) that quickly evolved in the decarboxylated compound 24. The final solution was extracted with ethyl acetate, and after the normal work-up, the residue was purified by recrystallization from ethanol. Yield 25%, white crystals, mp 246–247 °C. ^1^H-NMR (400 MHz, DMSO-d_6_) δ 9.14 (s, 1H, H-5); 8.43 (d, 1H, H-7, *J =* 4.8 Hz); 8.17 (s, 1H, H-2); 7.91 (d, 1H, H-8, *J =* 4.8 Hz); 6.83 (s, 1H, H-3). ^13^C-NMR (100 MHz, DMSO-d_6_) δ 149.90, 146.24, 144.70, 130.52, 126.70, 125.25, 119.15, 101.30. Anal. C_8_H_5_N_3_S (C, H, N).

**3-Methylpyrazolo[1,5-a]thieno[2,3-e]pyrimidine (25).** Compound **14** (0.34 mmol) was suspended in THF anhydrous (5 mL) and 1.50 mmol of LiAlH_4_ was added, using a 1M solution of LiAlH_4_ in THF. After 1 h, the starting material disappeared and adding ice/water quenched the reaction. The extraction with ethyl acetate gave the intermediate 4,5-dihydro derivative not isolated but identified since not fluorescent in TLC, which was treated with toluene and 10% Pd/C at reflux temperature until the dehydrogenation was complete. The final suspension was filtered, toluene was evaporated and the residue was recrystallized by ethanol; yield 35%, white crystals, mp 127–130 °C. TLC: toluene/ethyl acetate/methanol 8:2:1.5 *v*/*v*/*v* (Rf: 0.7); ^1^H-NMR (400 MHz, DMSO-d_6_) δ 9.06 (s, 1H, H-5); 8.39 (d, 1H, H-7, *J =* 5.2 Hz); 8.04 (s, 1H, H-2); 7.87 (d, 1H, H-8, *J =* 5.2 Hz); 2.33 (s, 1H, CH_3_). ^13^C-NMR (100 MHz, DMSO-d_6_) δ 149.90, 132.74, 132.90, 130.56, 126.70, 125.65, 119.10, 115.63, 14.30. Anal. C_9_H_7_N_3_S (C, H, N).

### 3.2. Molecular Docking and Molecular Dynamic Simulation

The structure of the binding site was obtained from the Human α1β2γ2-GABAA receptor subtype in complex with GABA and flumazenil, conformation B (PDB ID 6D6T) [21], considering all the amino acids within a distance of about 2 nm from the structure of the Flumazenil. The ligands were placed at the binding site through AUTODOCK 4.2 [20]. The molecular dynamics simulations of ligand binding-site complexes were performed on a minimum number of conformations (maximum 2) such as to cover at least 90% of the poses found by AUTODOCK. A 60 ns MD simulation were performed for all complexes using GROMACS v5.1 program, and it was conducted in vacuum [22]. The DS ViewerPro 6.0 program [19] was used to build the initial conformations of ligands. The partial atomic charge of the ligand structures was calculated with CHIMERA [23] using AM1-BCC method, and the topology was created with ACPYPE [24] based on the routine Antechamber [25]. The OPLS-AA/L all-atom force field [26] parameters were applied to all the structures. To remove bad contacts, the energy minimization was performed using the steepest descent algorithm until convergence is achieved or for 50,000 maximum steps. The next equilibration of the system was conducted in two phases:

(1) Canonical NVT ensemble, a 100 ps position-restrained of molecules at 300 K was carried out using a temperature coupling thermostat (velocity rescaling with a stochastic term) to ensure the proper stabilization of the temperature [27].
(2) Isothermal isobaric NPT ensemble, a 100 ps position-restrained of molecules at 300 K and 1 bar was carried out without using barostat pressure coupling to stabilize the system. These were then followed by a 60 ns MD run at 300 K with position restraints for all protein atoms. The Lincs algorithm [28] was used for bond constraints to maintain rigid bond lengths.

The initial velocity was randomly assigned taken from Maxwell–Boltzman distribution at 300 K and computed with a time step of 2 fs, and the coordinates were recorded every 0.6 ns for MD simulation of 60 ns. During the simulated trajectory, 100 conformations were collected. The ‘Proximity Frequencies’ (PFs) [1,2] with which the 100 conformations of each binding-site ligand complex intercepts two or more amino acid during the dynamic simulation have calculated. The ‘Proximity Frequency’ (PF) is the frequency with which the ligand was, during the molecular dynamic simulation, at a distance of less than 0.25 nm from an amino acid of the binding-site and also, simultaneously, from 2, 3 and 4 amino acids of the binding site.

## 4. Conclusions

The synthesis and the study on the reactivity of the new scaffold 5-oxo-4,5-dihydropyrazolo [1,5-a]thieno [2,3-e]pyrimidine was performed with the aim of isosteric replacement of the benzene ring in our 5-oxo-4,5-dihydro pyrazolo[1,5-a]quinazoline (PQ), already identified as α1β2γ2-GABA_A_R ligands.

The introduction in this new scaffold of those fragments responsible for the activity in our PQ [6,12] gave six final designed compounds (**12c**–**f** and **22a,b**), which were, in turn, studied in the ‘Proximity Frequency’ model [13] to predict their potential profile on the α1β2γ2-GABA_A_R.

The results indicate for all six products an agonist profile, highlighting the suitability of the nucleus and confirming the importance of the carbonyl group of the ester moiety to engage strong hydrogen bond interaction with the receptor protein. In particular, the esters derivatives (**12c** and **22b**) are able, through the carbonyl group, to interact with the amino acid residue γThr142 with a strong hydrogen bond (2.07 Å). Additionally, **22a** has an ester group in position 3, but the steric hindrance of the *t*-butyloxycarbonyl fragment makes the hydrogen bond interaction of the carbonyl group with the γThr142 residue less probable. Compounds missing the ester group (**12d**–**f**) show a weak interaction with γThr142 in agreement with the low prediction percentage.

In conclusion, the synthesis of the 5-oxo-4,5-dihydropyrazolo [1,5-a]thieno [2,3-e]pyrimidine scaffold allowed us to identify a new chemical class of compounds potentially active on GABA_A_ receptor subtype, and the in silico results should be completed and confirmed with biological assays.

## Figures and Tables

**Table 1:** Profile prediction obtained by PF model (LDA results).

N°	Prediction	% Ago	% Anta
**12d**	ago	66	34
**12e**	ago	64	36
**12f**	ago	64	36
**12c**	**AGO**	**78**	22
**22a**	**AGO**	**69**	31
**22b**	**AGO**	**74**	26

## Data Availability

Not applicable.

## Supplementary Materials

The following supporting information can be downloaded at: https://www.mdpi.com/article/10.3390/molecules28073054/s1, Chemistry. Materials and methods; ^1^H-NMR spectra of all compounds; ^13^C-NMR spectra of some representative compounds (**6**, **8**, **10c**, **11c**, **11e**, **12c**, **12d**, **12e**, **12f**, **22a**, **22b, 23**); Elemental analysis (Table S1); General chemical structure with atoms numeration.
