# Supplementary material for: 4,5-Dihydro-5-Oxo-Pyrazolo[1,5-a]Thieno[2,3-c]Pyrimidine: A Novel Scaffold Containing Thiophene Ring. Chemical Reactivity and In Silico Studies to Predict the Profile to GABAA Receptor Subtype
Source: Molecules. 2023 Mar 29;28(7):3054. doi: 10.3390/molecules28073054 (PMC10095790; doi:10.3390/molecules28073054)
Supplement: Supplementary file 1 [file molecules-28-03054-s001.zip › molecules-2302871-supplementary.pdf]

Supporting Information

For

4,5-Dihydro-5-oxo-pyrazolo[1,5-a]thieno[2,3-c]pyrimidine: a novel scaffold containing thiophene ring. Chemical reactivity and *in silico* studies to predict the profile to GABAA receptor subtype

Letizia Crocetti [1], Gabriella Guerrini [1]*, Fabrizio Melani [1], Claudia Vergelli[1], and Maria Paola Giovannoni[1]

[1]Neurofarba, Pharmaceutical and Nutraceutical Section, University of Florence, Via Ugo Schiff 6, 50019, Sesto Fiorentino, Italy.

*Correspondence: gabriella.guerrini@unifi.it (GG); Tel.: Tel.: +39-055-4573766

Table of contents:

- Chemistry. Materials and methods.
- ^{1}H-NMR spectra of all compounds
- ^{13}C-NMR spectra of some representative compounds (**6**, **8**, **10c**, **11c**, **11e**, **12c**, **12d**, **12e**, **12f**, **22a**, **22b**, **23**)
- Elemental analysis (Table S1)
- General chemical structure with atoms numeration

Chemistry

Materials and Methods

Reagents and starting materials were obtained from commercial sources. Extracts were dried over Na_2SO_4, and the solvents were removed under reduced pressure. All reactions were monitored by thin layer chromatography (TLC) using commercial plates pre-coated with Merck silica gel 60 F-254. Visualization was performed by UV fluorescence (λ_{max} = 254 nm) or by staining with iodine or potassium permanganate. Chromatographic separations were performed on a silica gel column by gravity chromatography (Kieselgel 40, 0.063-0.200 mm; Merck), flash chromatography (Kieselgel 40, 0.040-0.063 mm; Merck), silica gel preparative TLC (Kieselgel 60 F_{254}, 20 x 20 cm, 2 mm). Yields refer to chromatographically and spectroscopically pure compounds, unless otherwise stated. Compounds were named following IUPAC rules, as applied by Beilstein-Institut AutoNom 2000 (4.01.305) or CA Index Name. All melting points were determined on a microscope hot stage Büchi apparatus and are uncorrected. Monodimensional spectra ^{1}H-NMR and ^{13}C-NMR were registered by a 400 MHz field through Avance 400 apparatus (Bruker Biospin Version 002 with SGU). Chemical shifts (d) are in parts per million (ppm) approximated by the nearest 0.01 ppm, using the solvent as internal standard. Coupling constants (J) are in Hz, they were calculated by Top Spin 3.1 and approximated by 0.1 Hz. Data are reported as follows: chemical shift, multiplicity (exch, exchange; br, broad; s, singlet; d, doublet; t, triplet; q, quartet; m, multiplet; or a combination of those e.g: dd), integral, assignments, and coupling constant. High resolution mass spectrometry (HRMS) analysis was performed with a Thermo Finnigan LTQ Orbitrap mass spectrometer equipped with an electrospray ionization source (ESI). The solvents used in HRMS were acetonitrile (Chromasolv grade) purchased from Sigma-Aldrich (Milan, Italy) and mQ water 18 MΩ cm, obtained from Millipore's Simplicity system (Milan, Italy). The accurate mass measure was performed by introducing sample solution (1.0 µg mL^{-1} in mQ water: acetonitrile 50:50) via syringe pump at 10 µL min^{-1}, and the signal of the positive ions was acquired. The experimental conditions allowed monitoring of the protonated molecules ($[M+H]^+$ species) with a proper dwell time to achieve 60.000 units of resolution at full width at half maximum (FWHM). All new compounds possess a purity ≥95%; microanalyses indicated by the symbols of the elements were performed with a Perkine-Elmer 260 elemental analyzer for C, H, and N, and they were within ±0.4% of the theoretical values.

Methyl 3-hydrazinothiophene-2-carboxylate hydrochloride (2). The commercial starting material, methyl 3-aminothiphene-2-carboxylate **1** (8.9 mmol, 1.4 g), was solubilized in 15 mL of HCl 6N and cooled at 0 °C. To this solution was dripped $NaNO_2$ (10 mmol, 0.70 g/4 mL H_2O),

maintaining the temperature under 5 °C and under stirring for 20 minutes. Then, a solution of $SnCl_2$ (28 mmol, 6.4 g/10 mL of HCl 12M) was slowly added, and the suspension was maintained under stirring for 50 minutes. Finally, the pale yellow precipitate was filtered and washed with HCl 12M. Recrystallization solvent: ethanol; yield 97%; mp 174-175 °C. TLC: toluene/ethyl acetate/methanol 8:2:1.5 v/v/v; ^{1}H-NMR (400 MHz, DMSO-d_6) δ 9.63 (bs, 2H, NH_2, exch.); 8.32 (s, 1H, NH, exch.); 7.83 (d, 1H, H-5, *J* = 5.6 Hz); 7.05 (d, 1H, H-4, *J* = 5.6 Hz); 3.76 (s, 3H, CH_3). Anal. $C_6H_8N_2O_2SCl$ (C, H, N). (The hydrazine was obtained by a different synthetic route than the one reported in ref [14] in the main manuscript, but the melting point is comparable to ref [14]).

[14] Huddleston, P.R.; Barker, J.M.; Adamczewska, Y. Preparation and reactions of some 3-hydrazino-2-methoxycarbonylthiophene. *J. Chem. Res.* **1980**, 238-239.

1H NMR Compound 3
DMSO

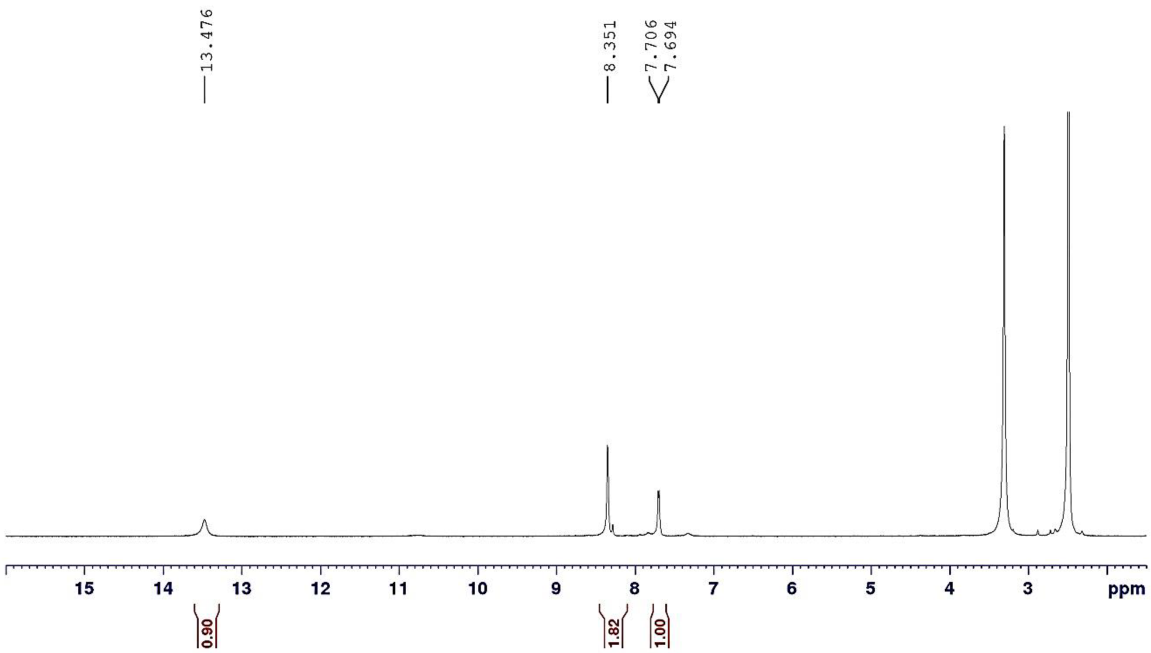

1H NMR Compound 3
DMSO + D2O

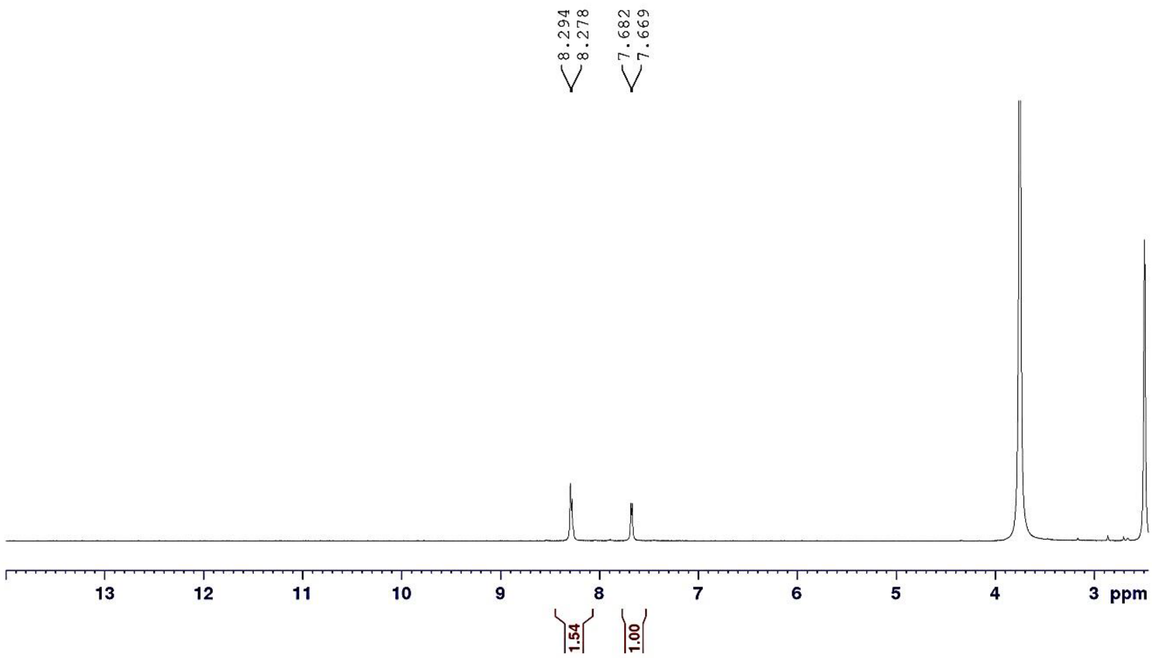

1H NMR Compound 4
DMSO

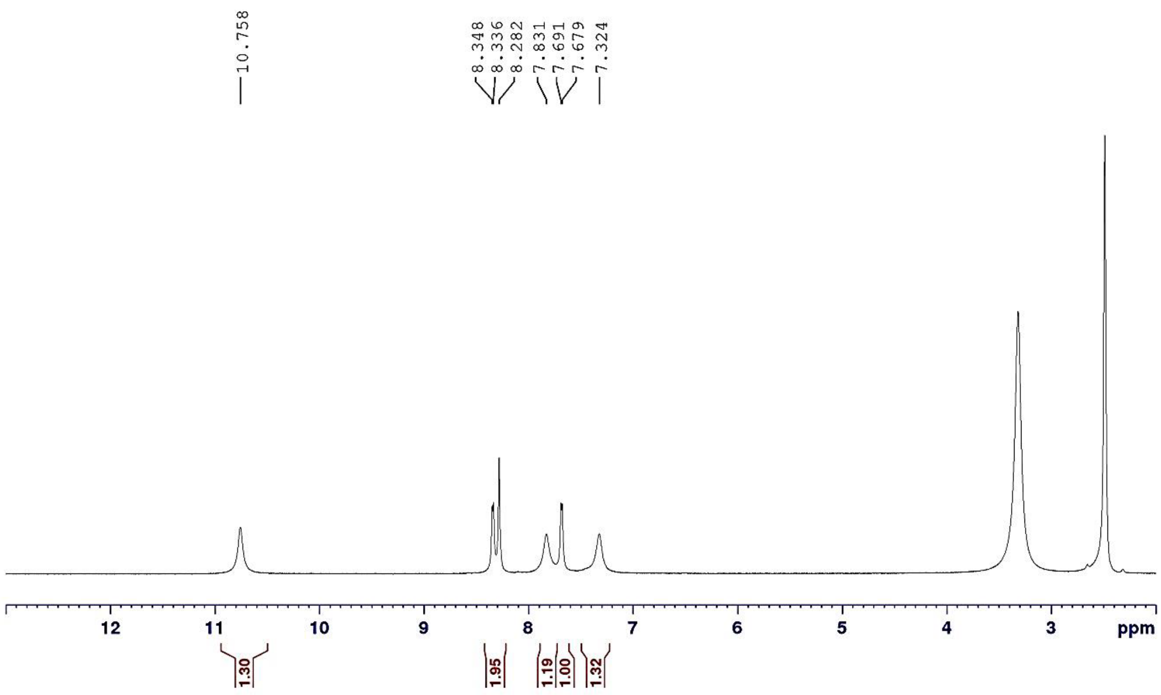

1H NMR Compound 4
DMSO + D2O

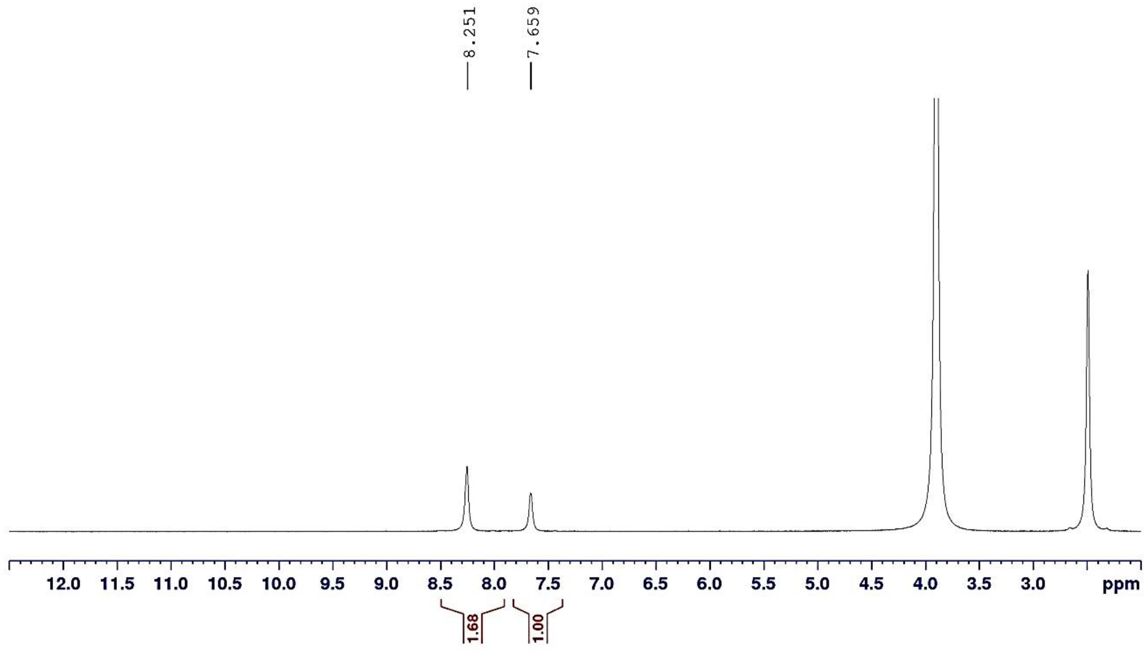

1H NMR Compound 5
CDCl3

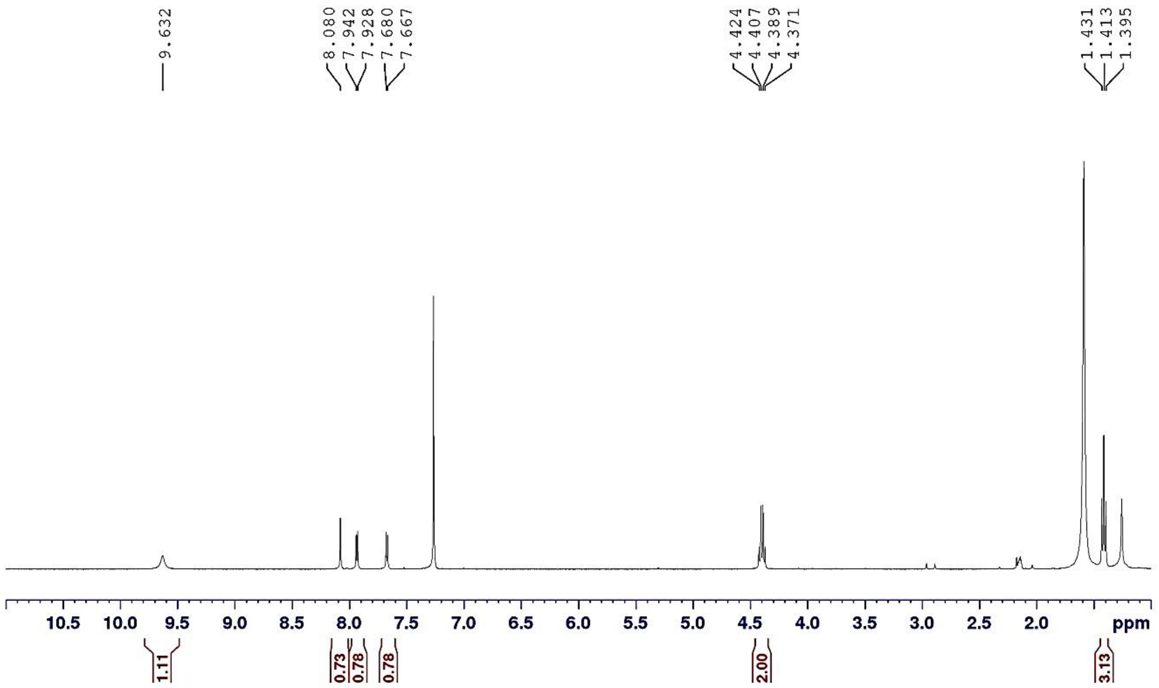

1H NMR Compound 6
DMSO

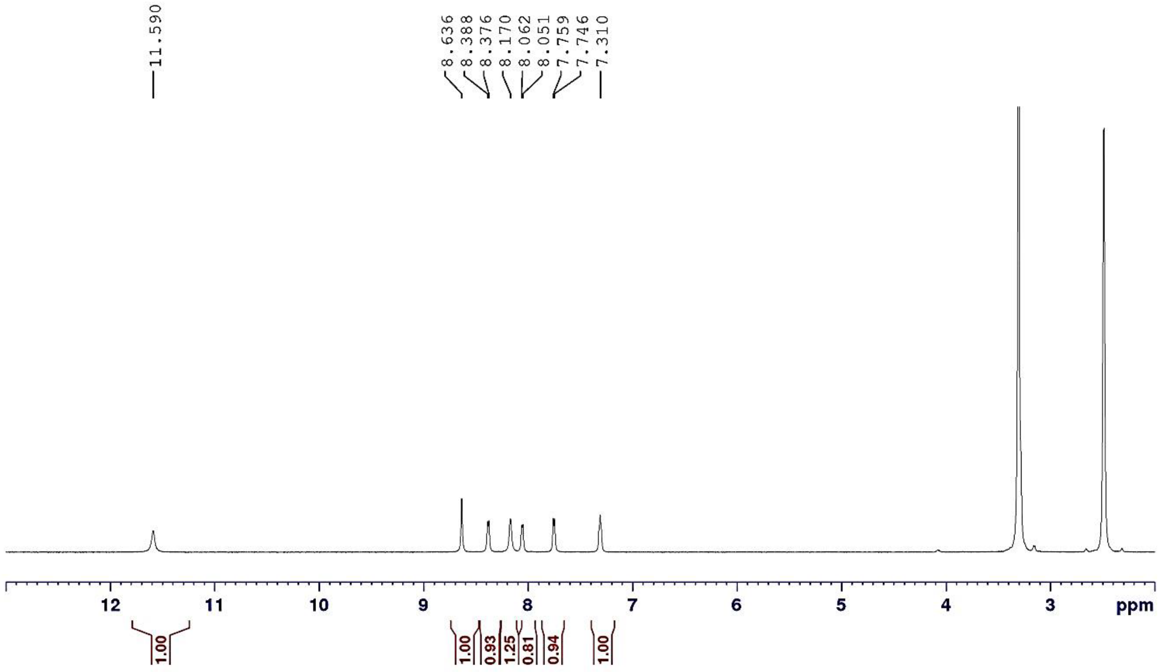

1H NMR Compound 7
DMSO

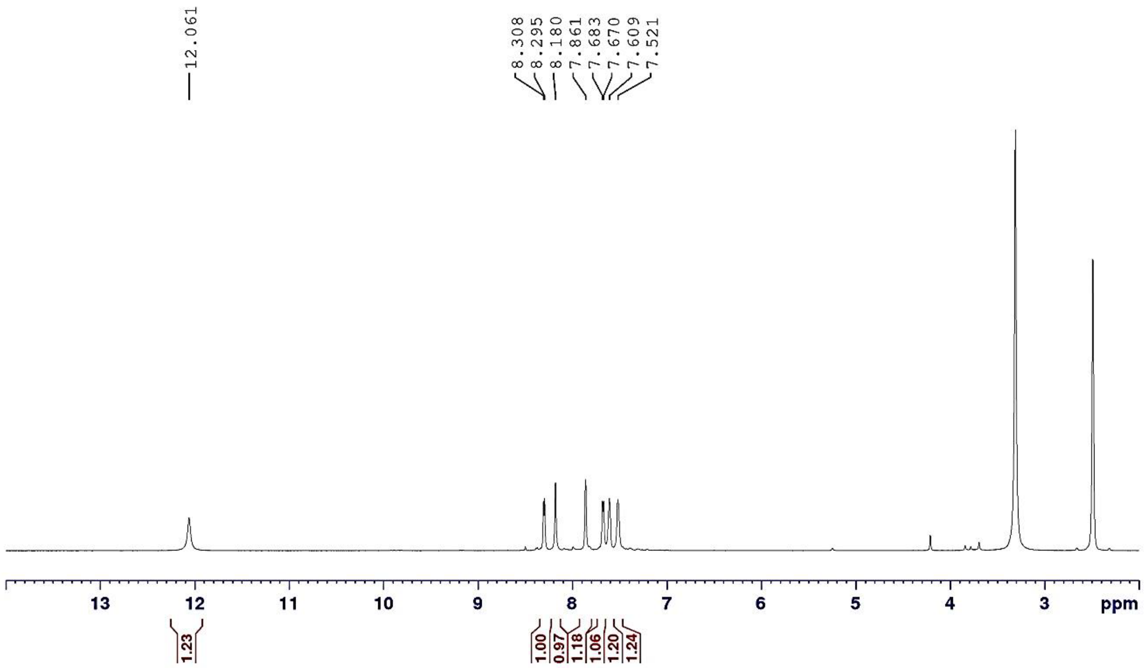

1H NMR Compound 8
DMSO

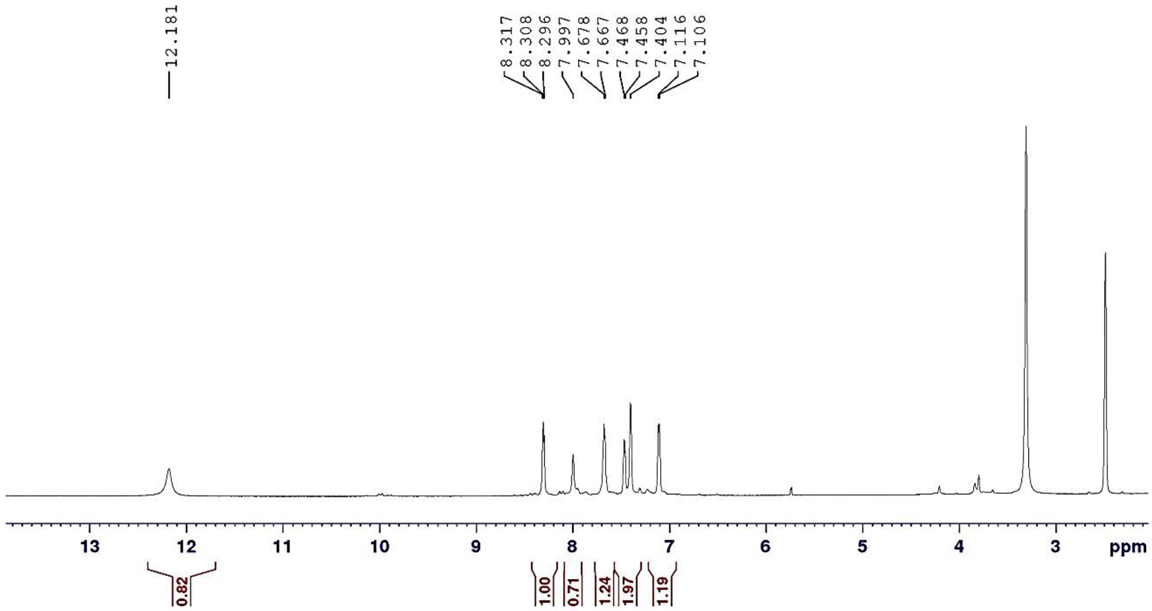

1H Compound 9c
CDCl3

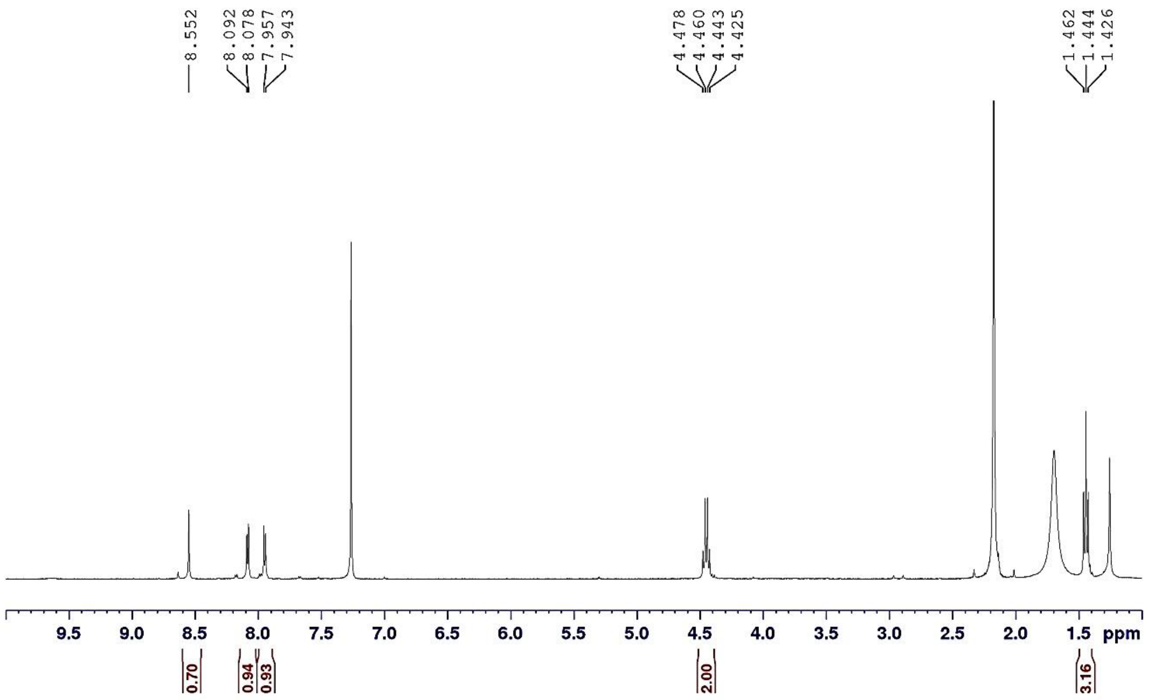

1H NMR Compound 9d
DMSO

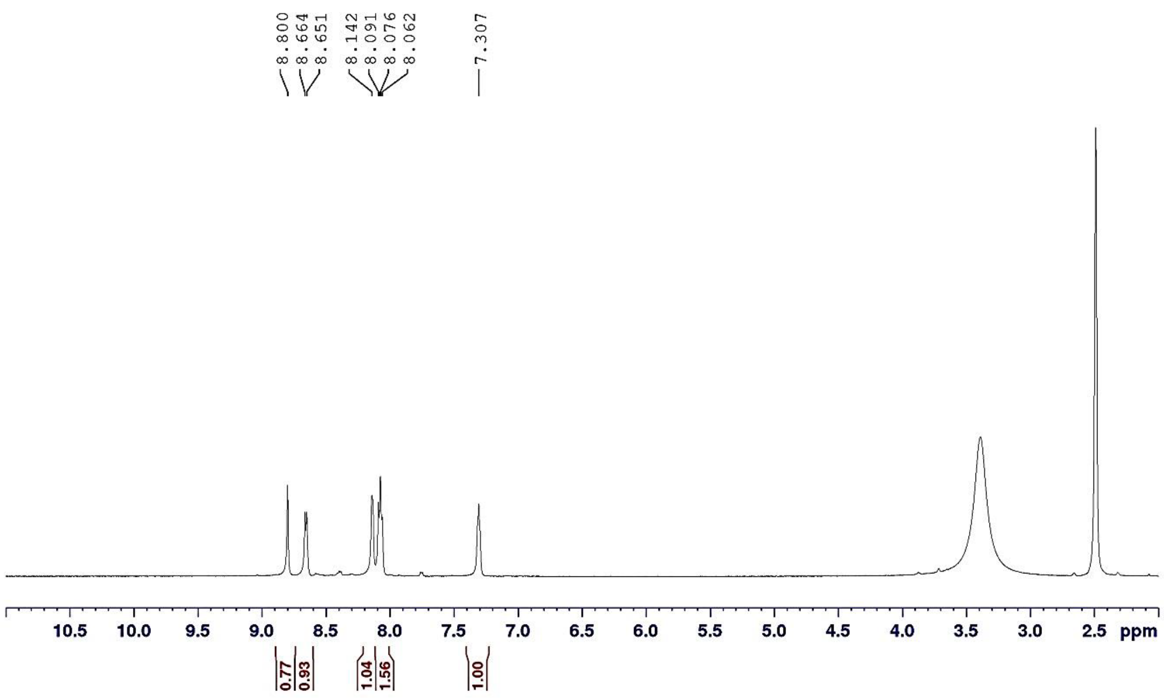

1H NMR Compound 10c
DMSO

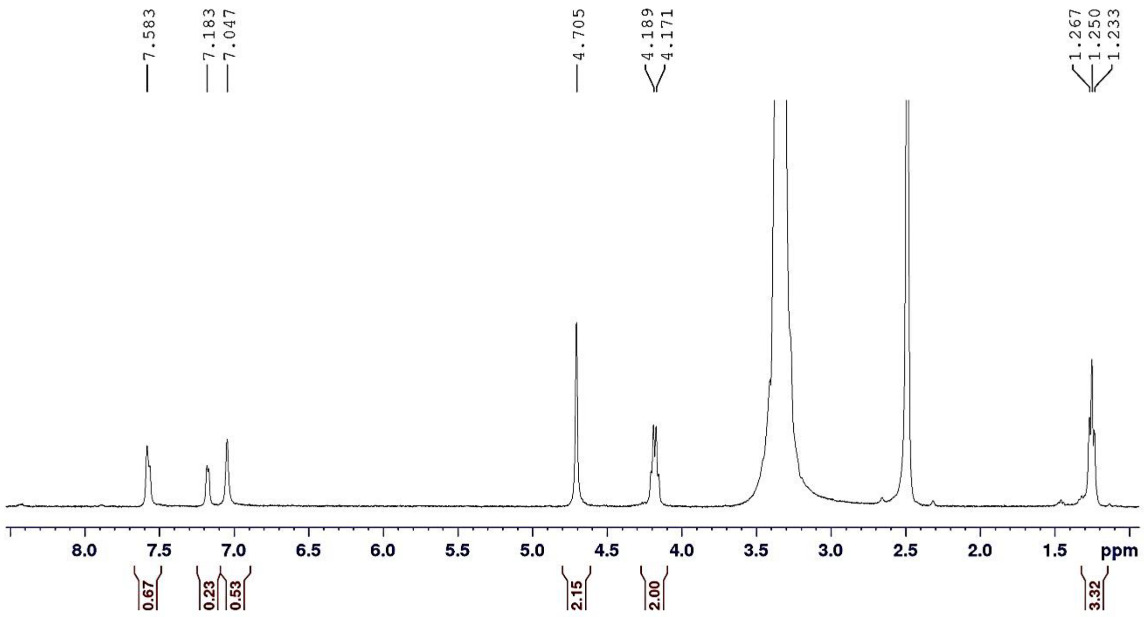

1H NMR Compound 10d
DMSO

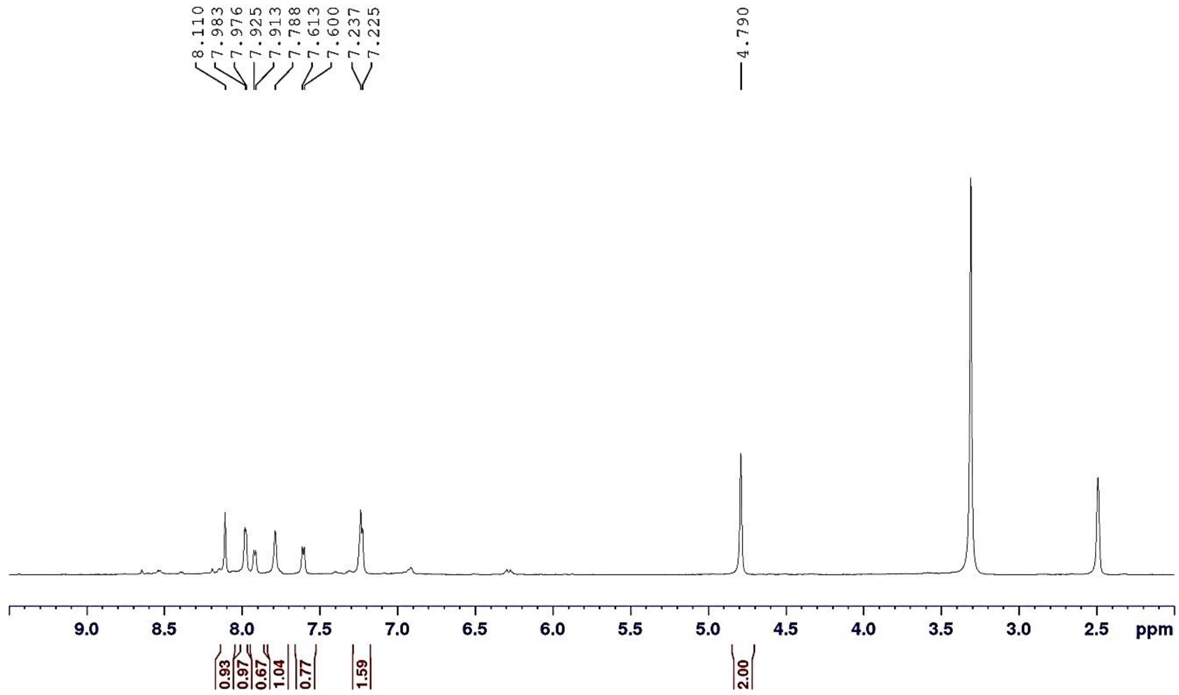

1H NMR Compound 10d
DMSO + D2O

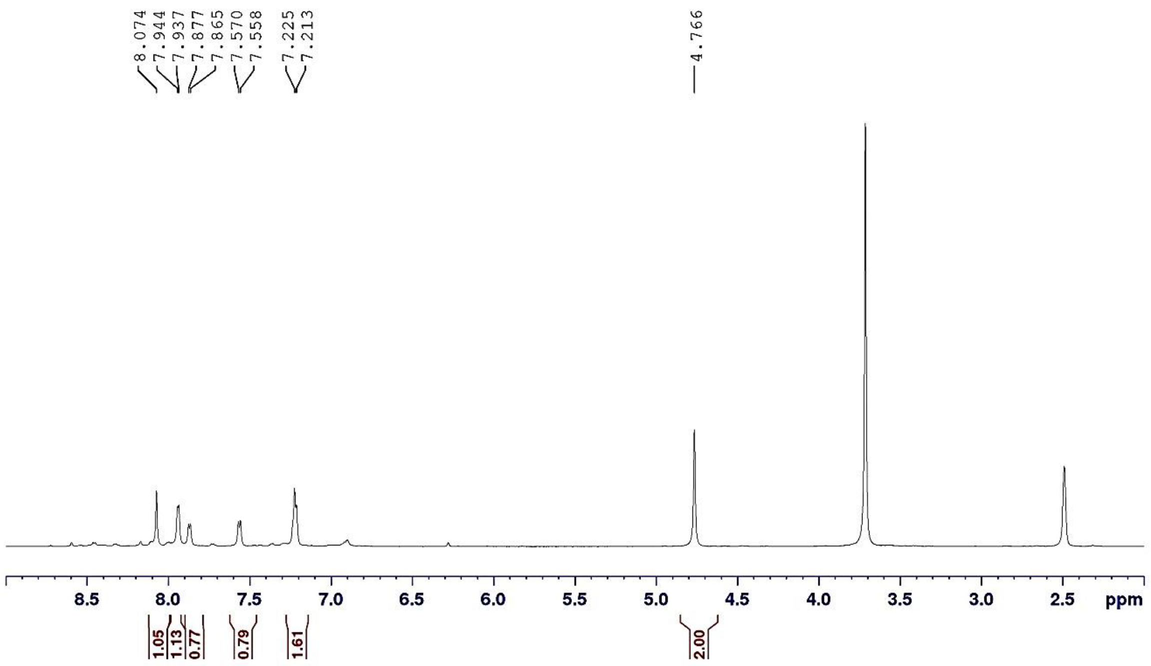

1H NMR Compound 11c
DMSO

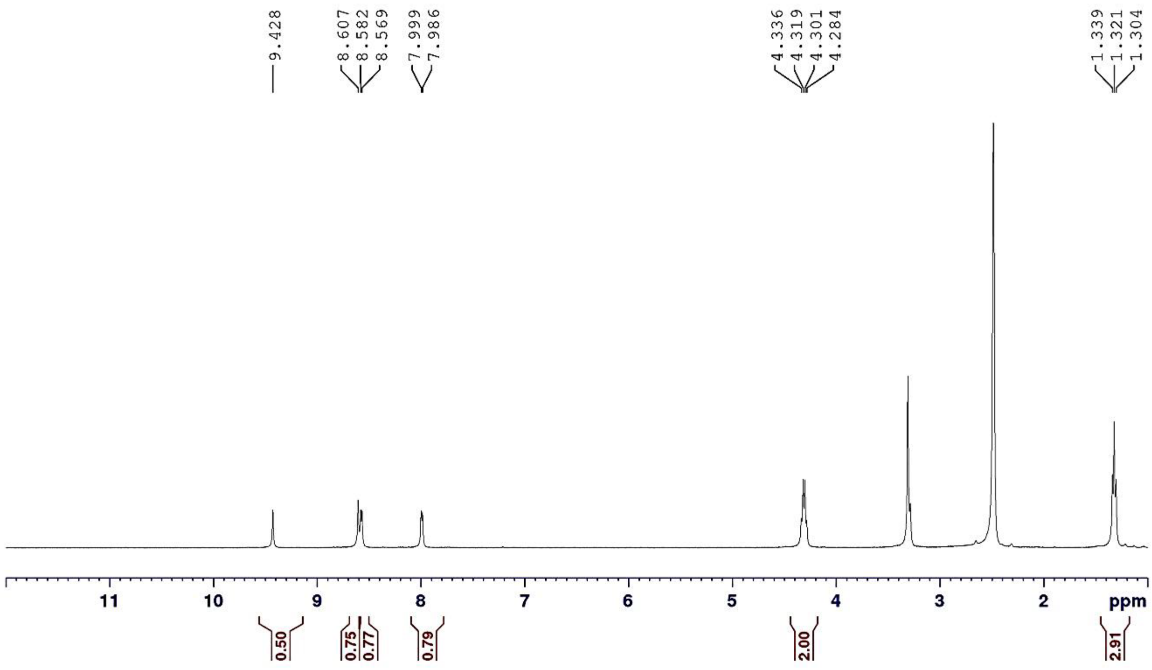

1H NMR Compound 11d
DMSO

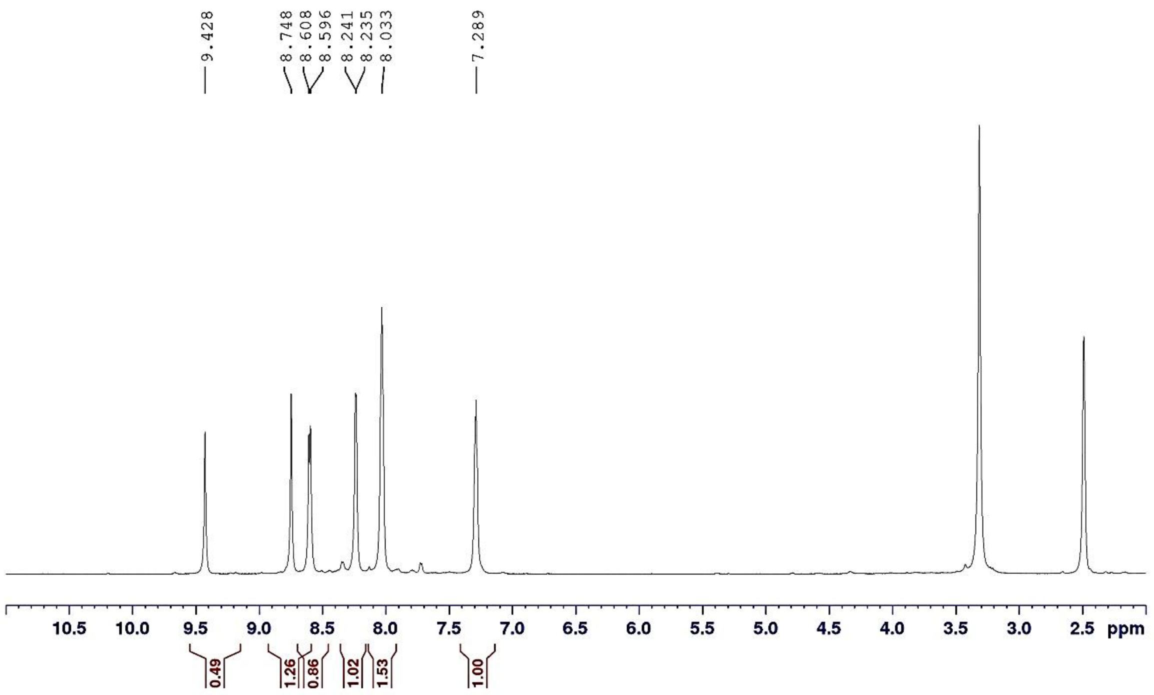

1H NMR Compound 11e
DMSO

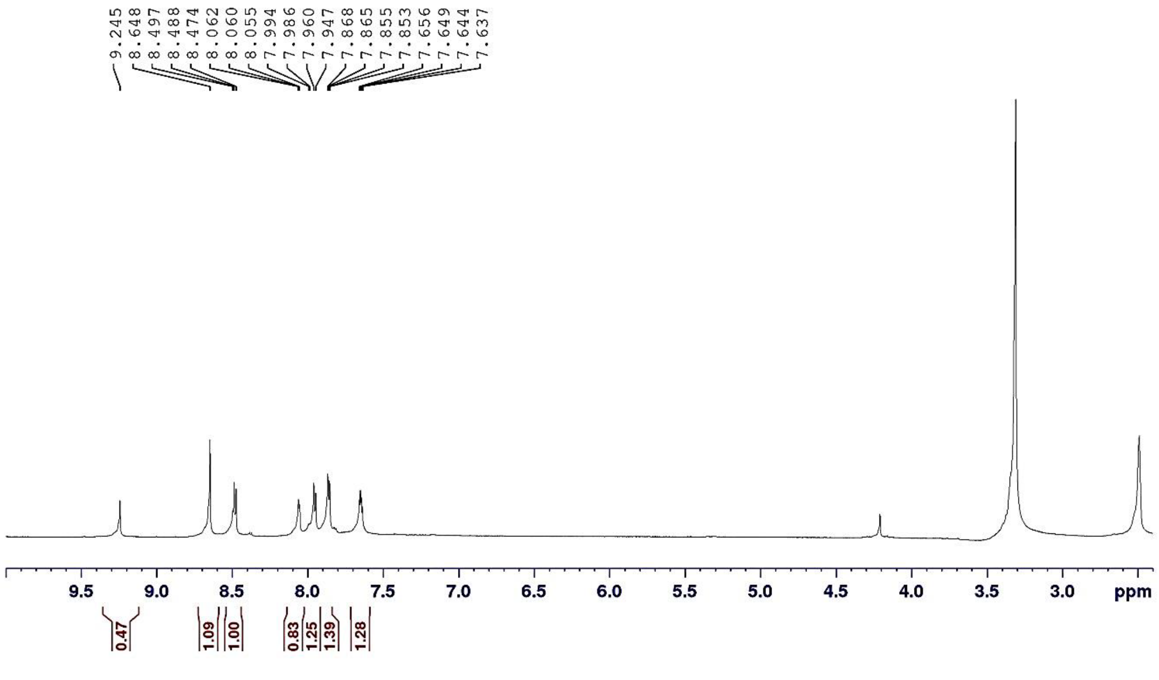

1H NMR Compound 12a
DMSO

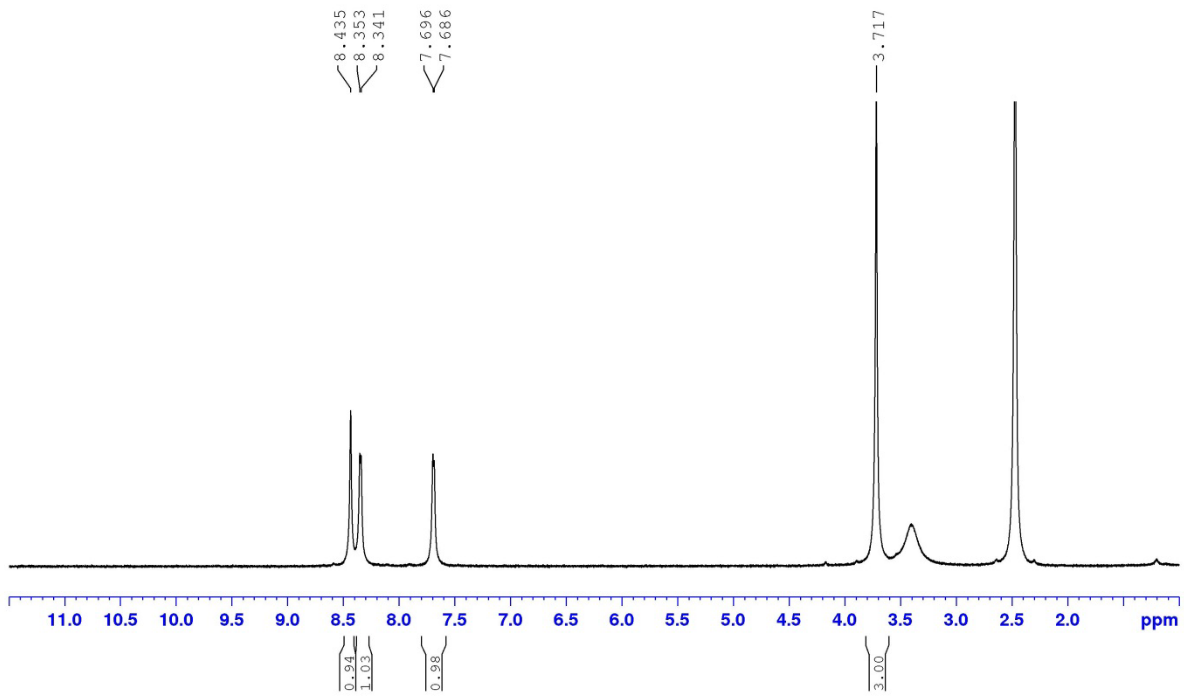

1H NMR Compound 12b
DMSO

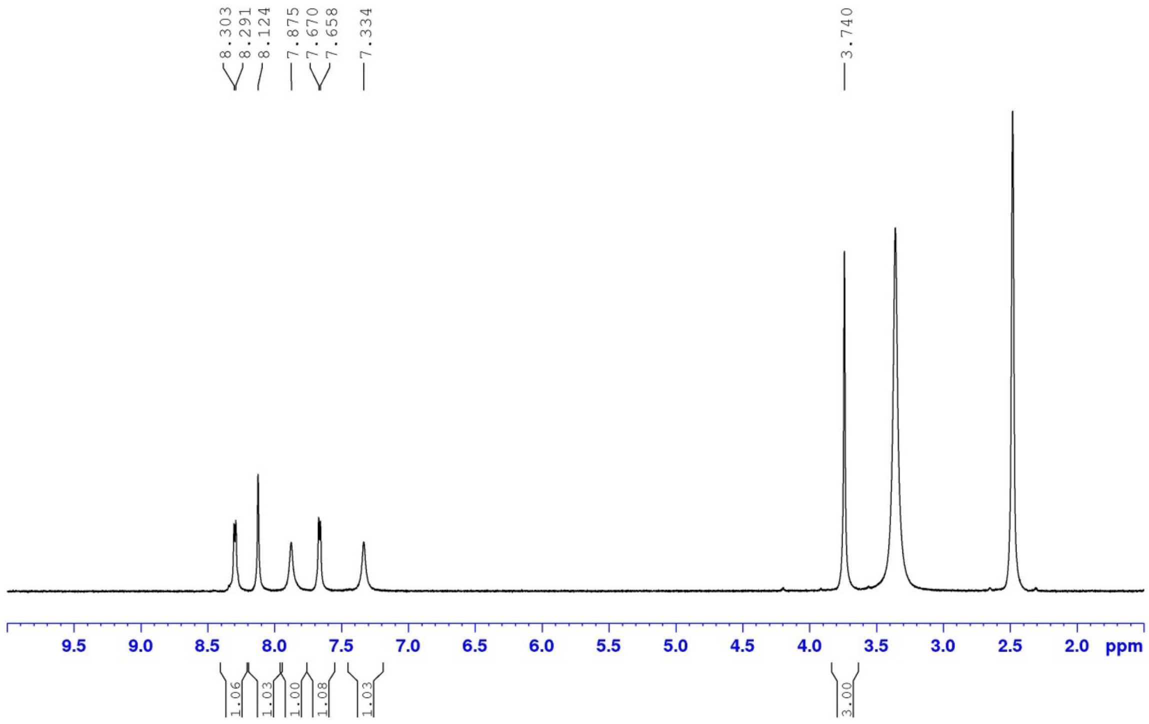

1H NMR Compound 12c
DMSO

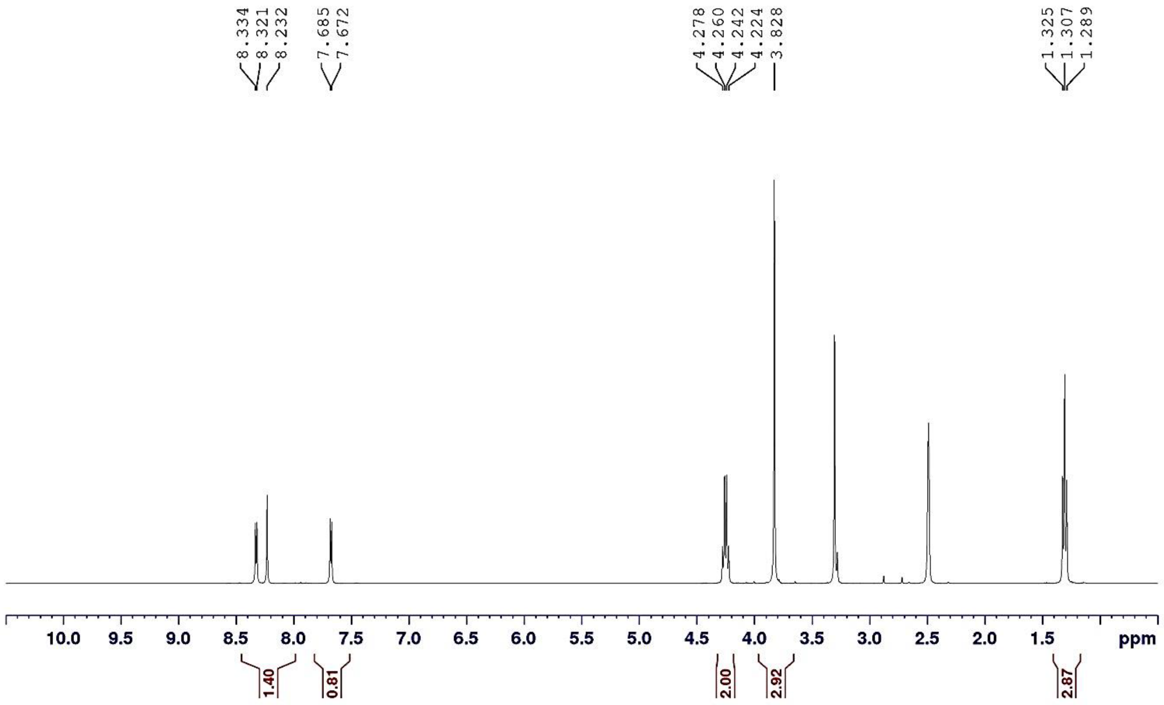

1H NMR Compound 12d
DMSO

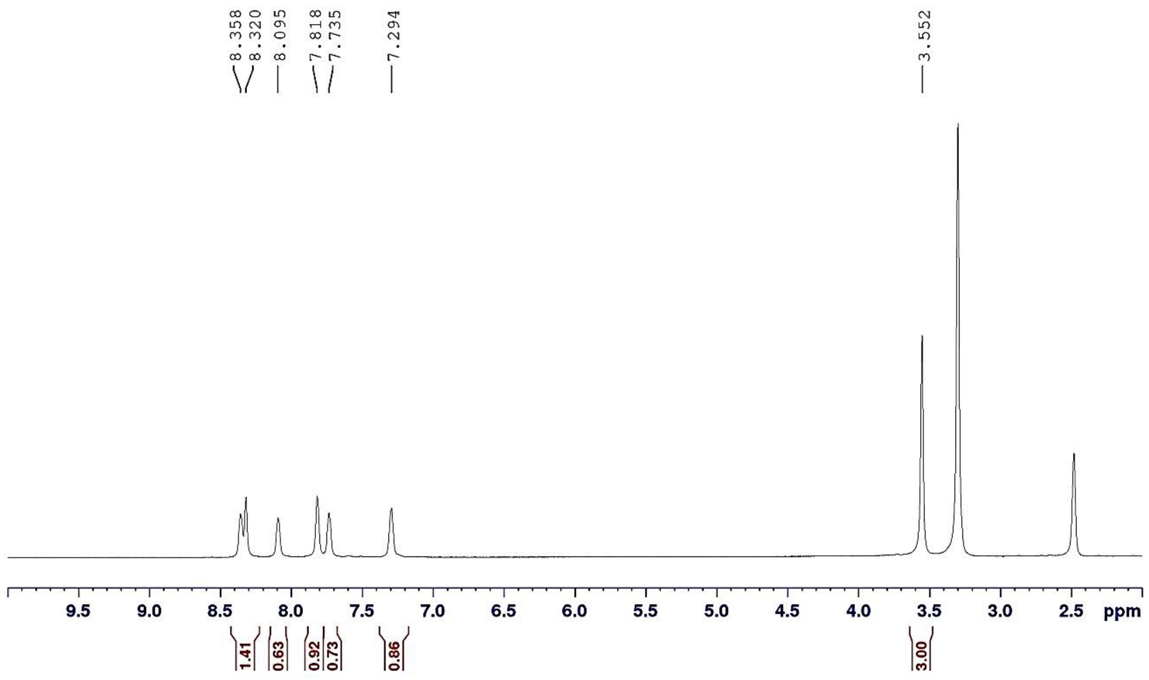

1H NMR Compound 12e
CDCl3

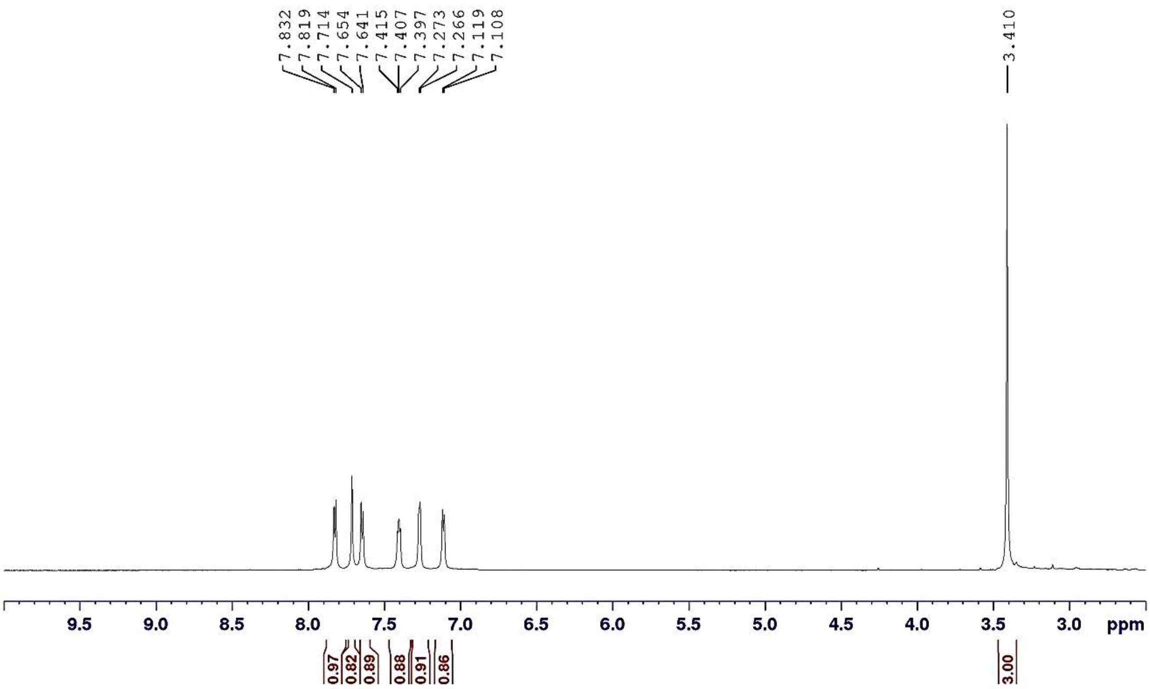

1H NMR Compound 12f
DMSO

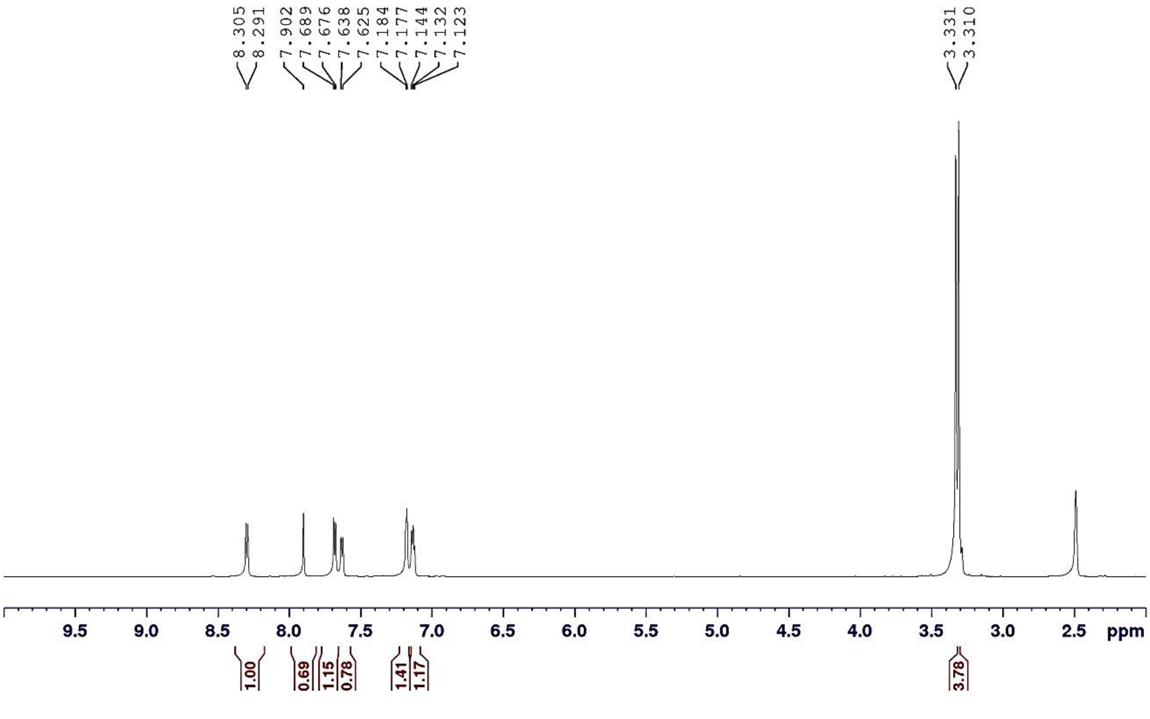

1H NMR Compound 13b
DMSO

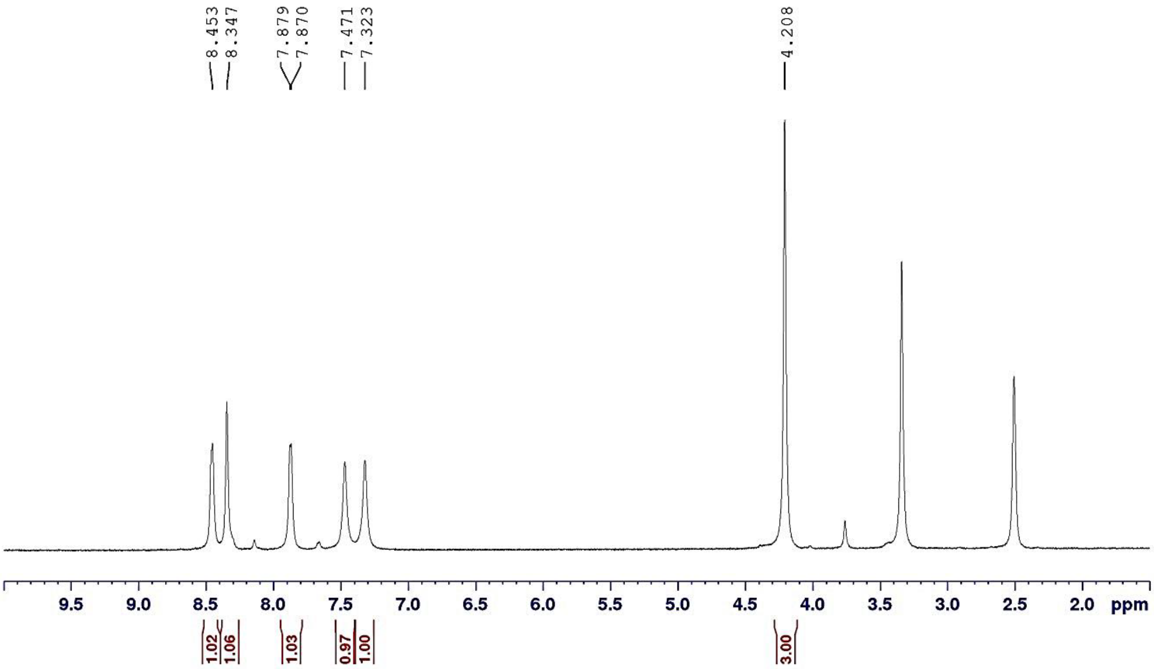

1H NMR Compound 13e
DMSO

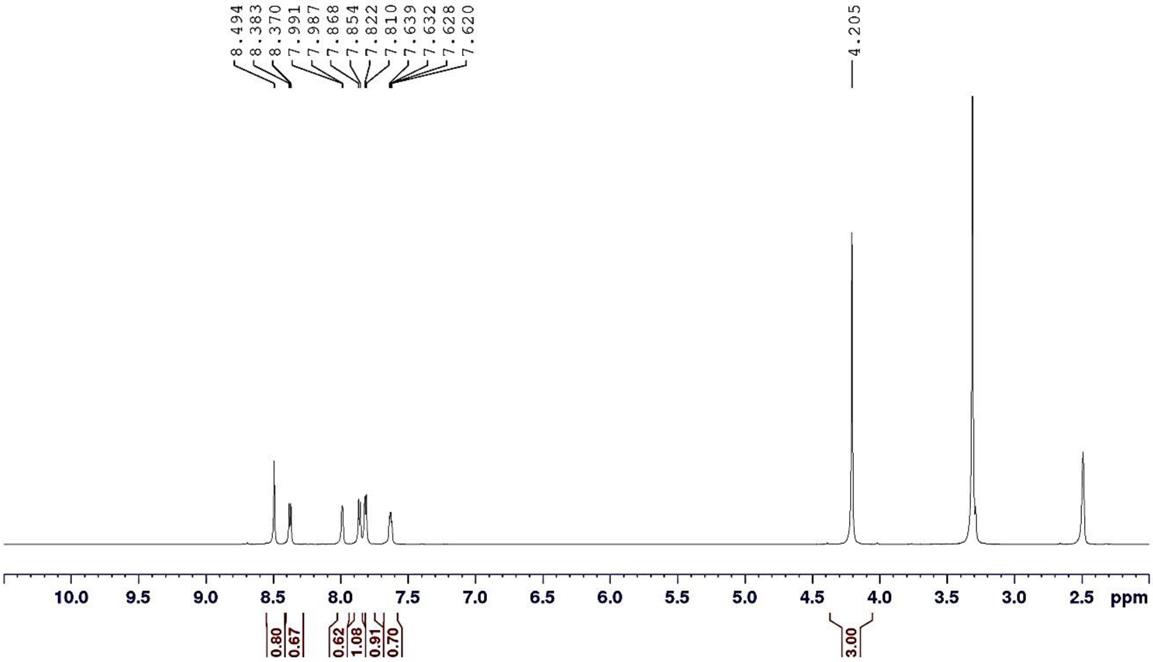

1H NMR Compound 13f
DMSO

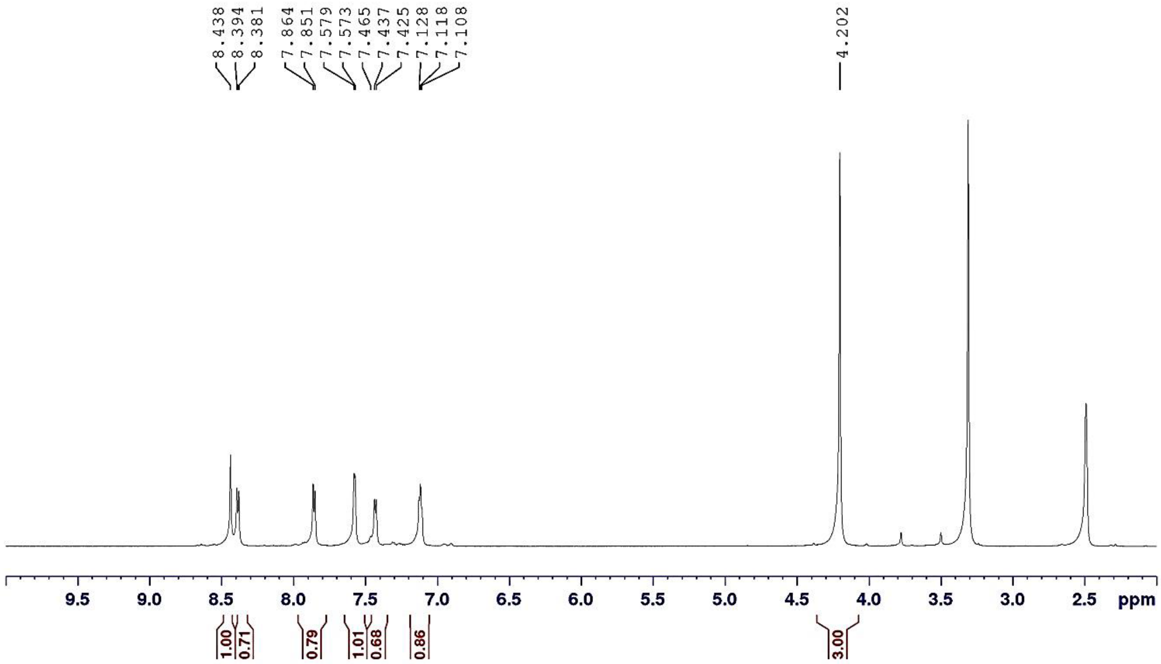

1H NMR Compound 14
DMSO

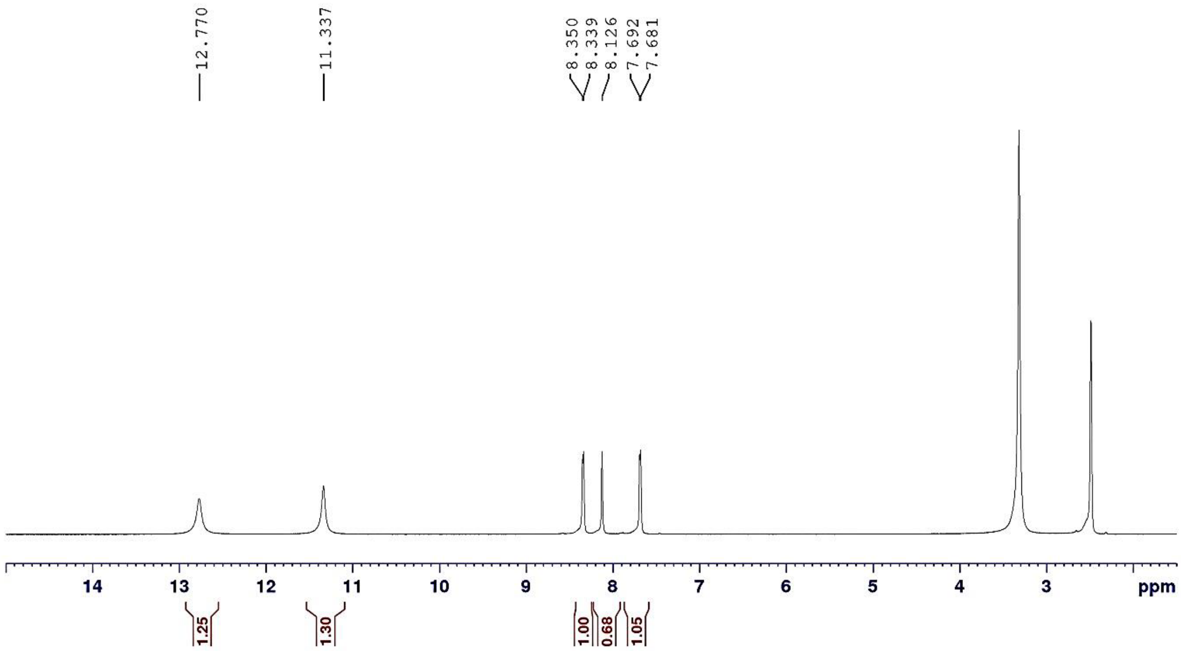

1H NMR Compound 14
DMSO + D2O

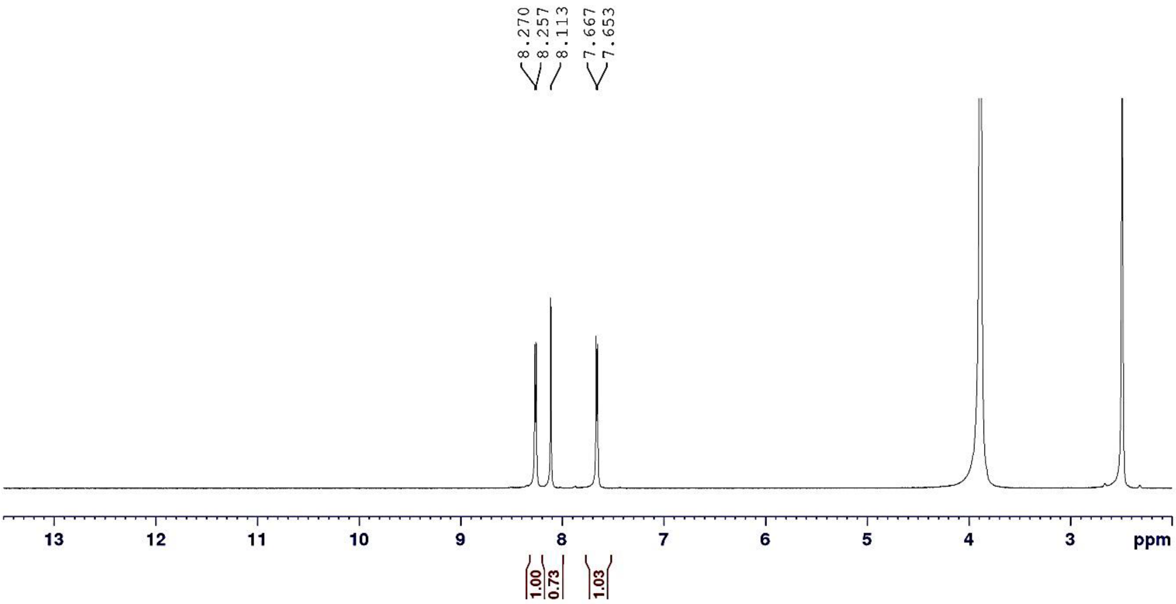

1H NMR Compound 15
DMSO

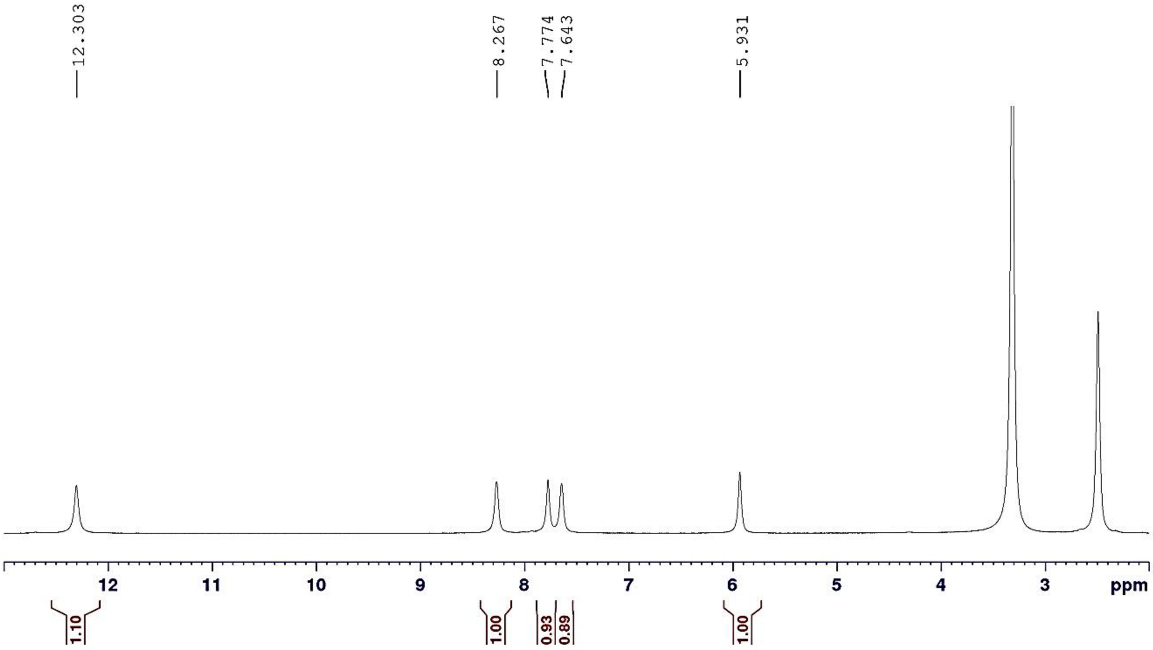

1H NMR Compound 15
DMSO + D2O

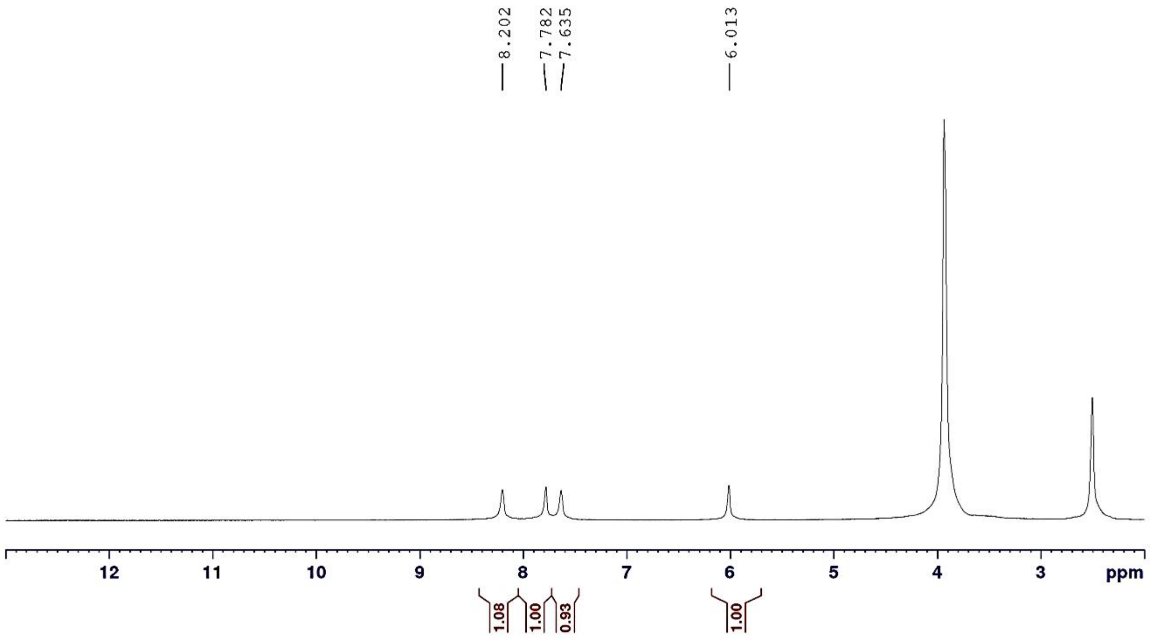

1H NMR Compound 16
CDCl3

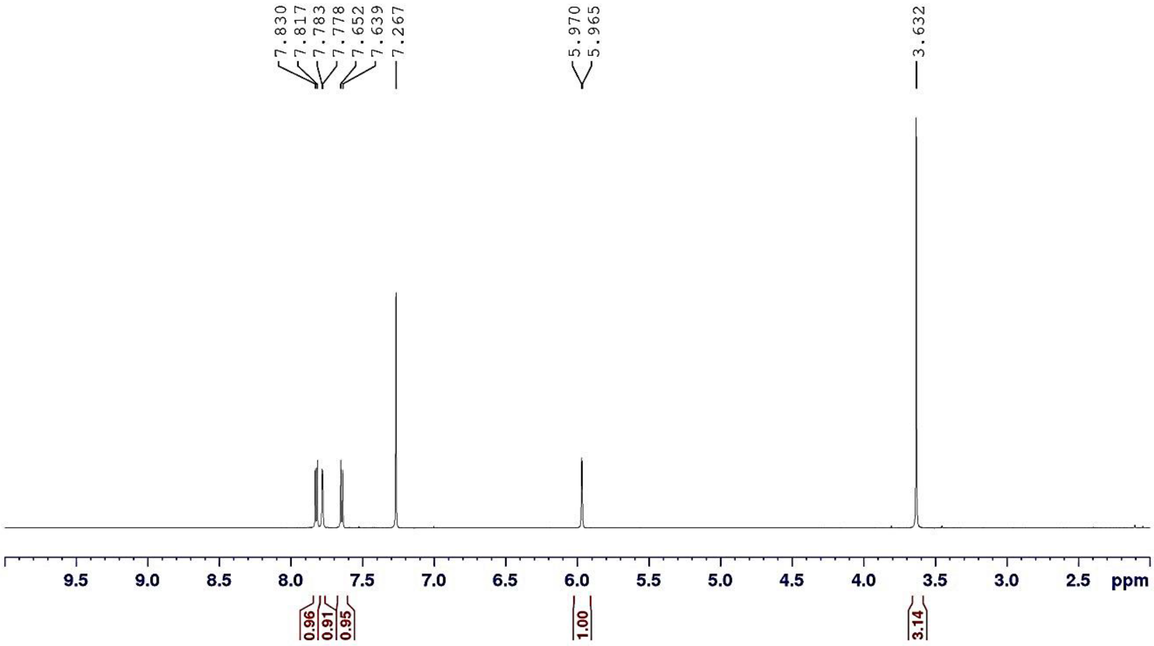

1H NMR Compound 17
DMSO

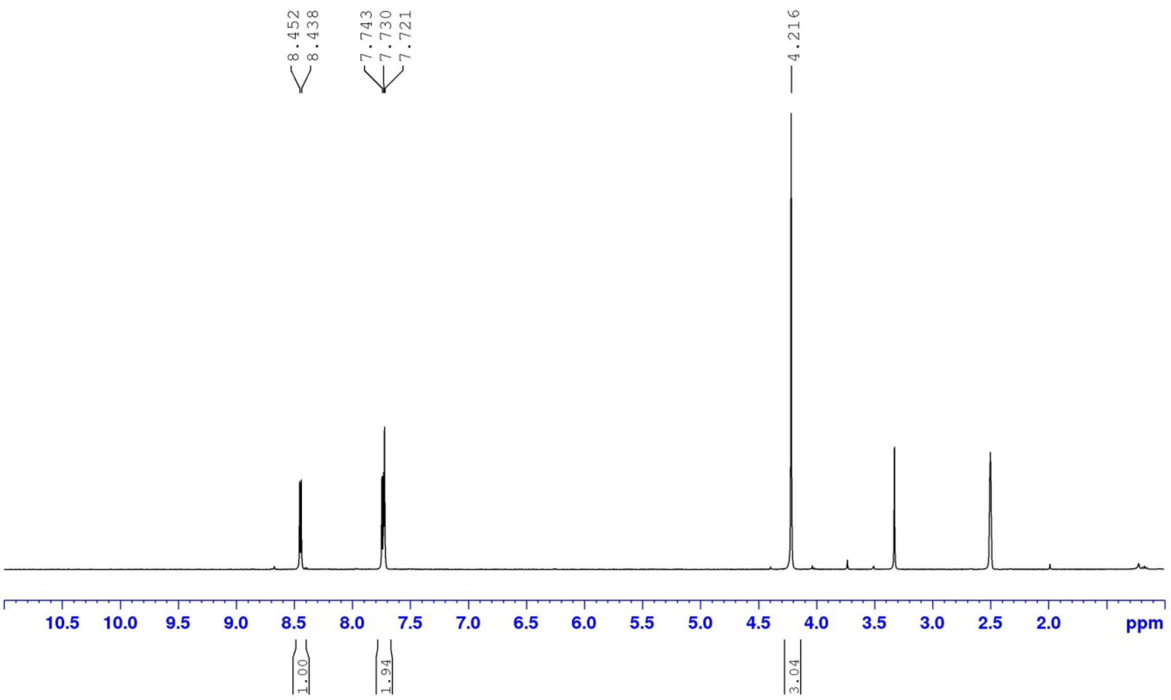

1H NMR Compound 19
DMSO

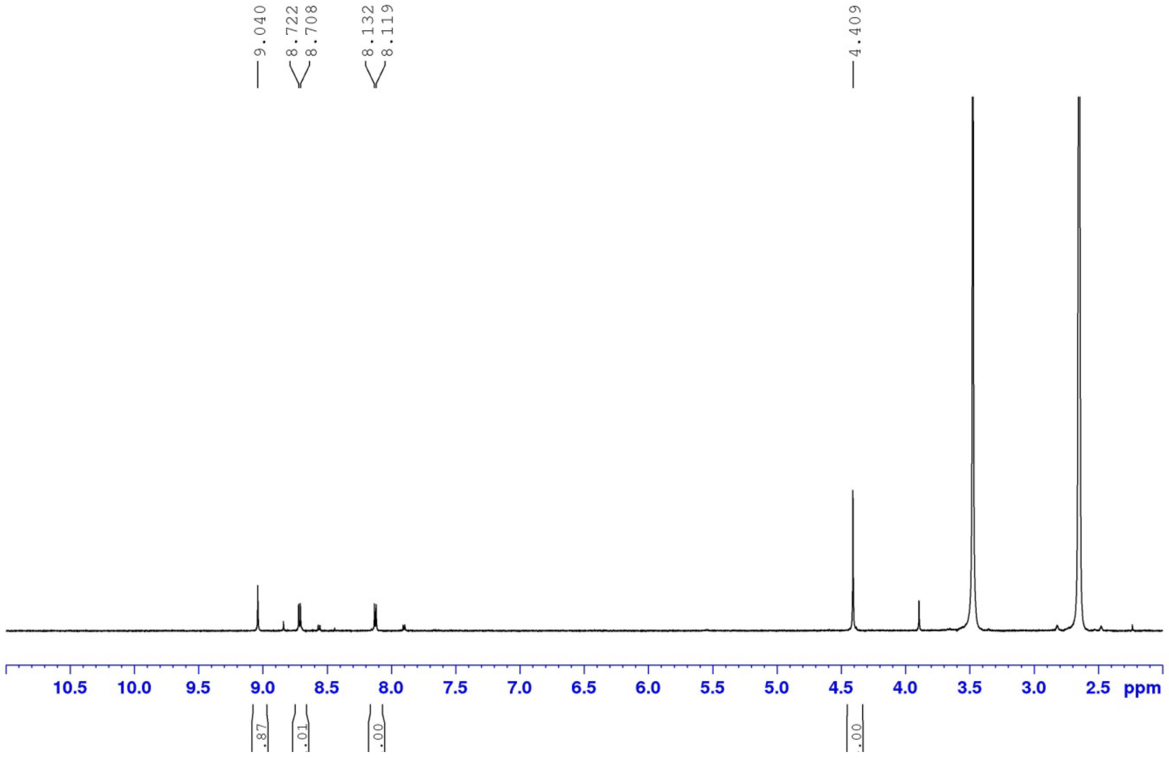

1H NMR Compound 20
CDCl3

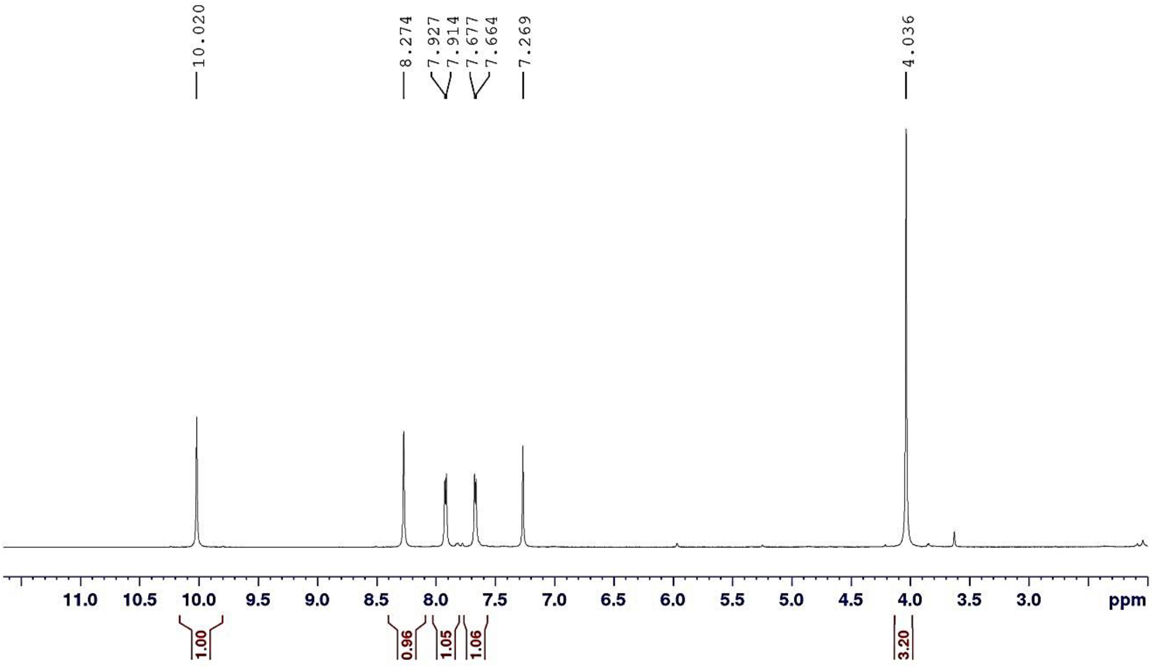

1H NMR Compound 21
DMSO

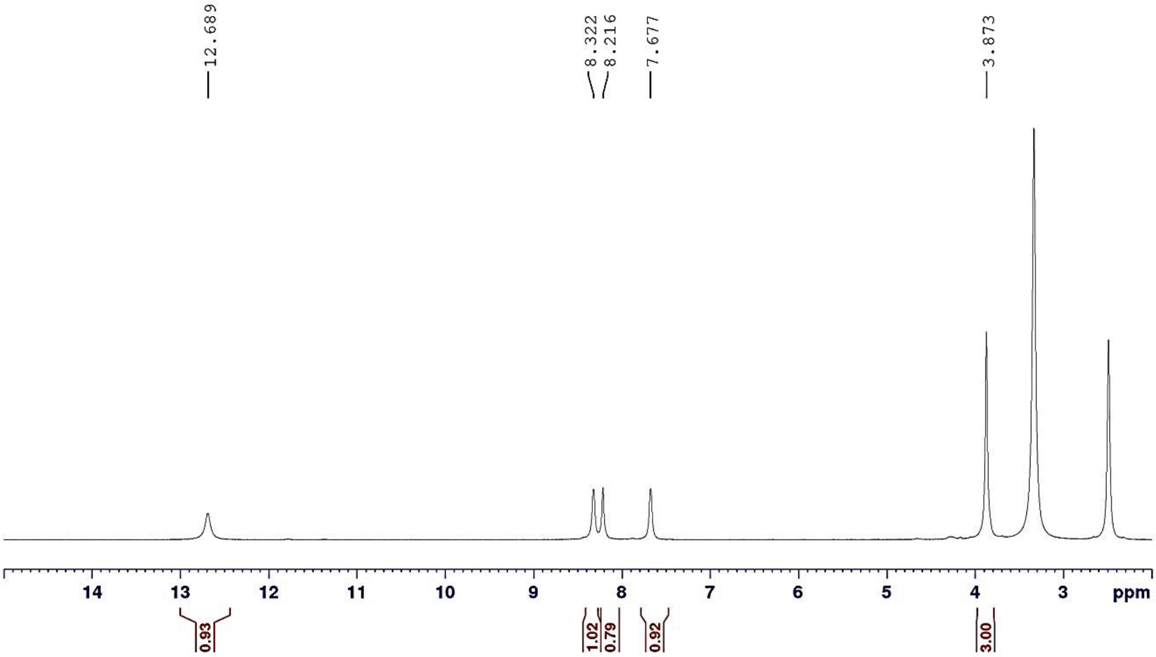

1H NMR Compound 21
DMSO + D2O

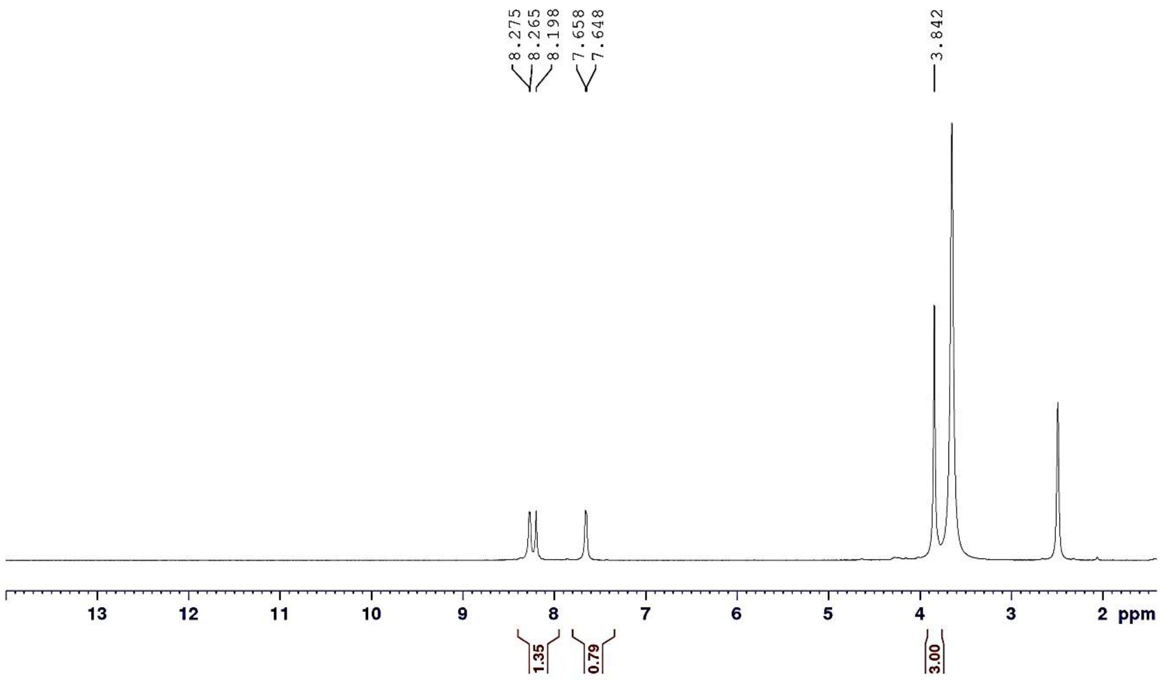

1H NMR Compound 22a
CDCl3

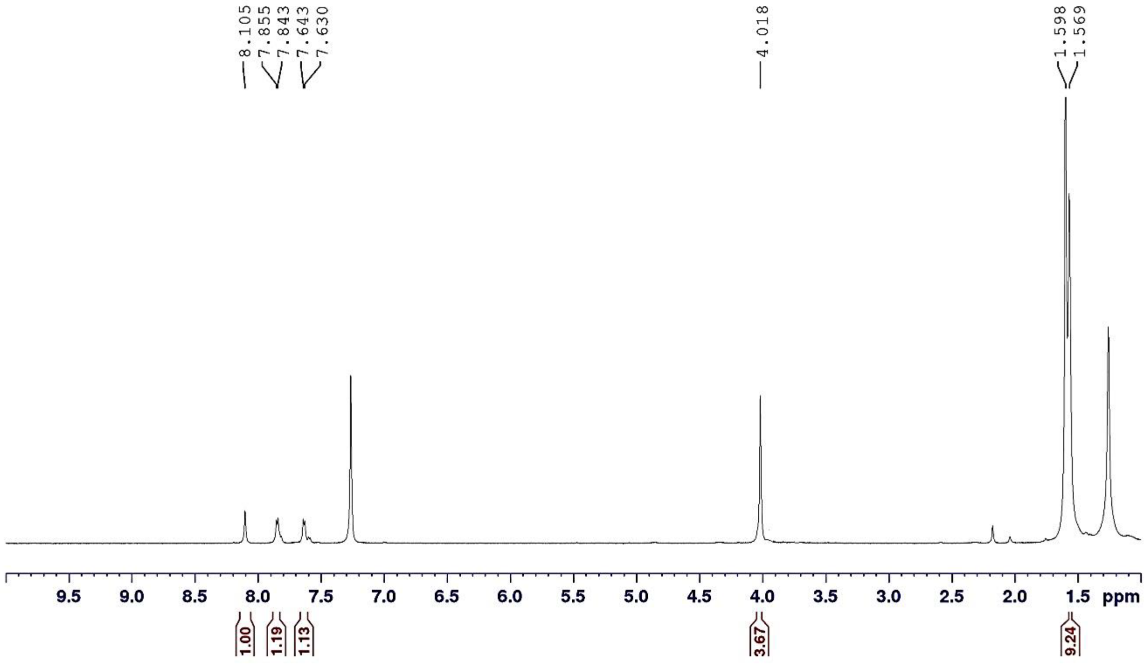

1H NMR Compound 22b
CDCl3

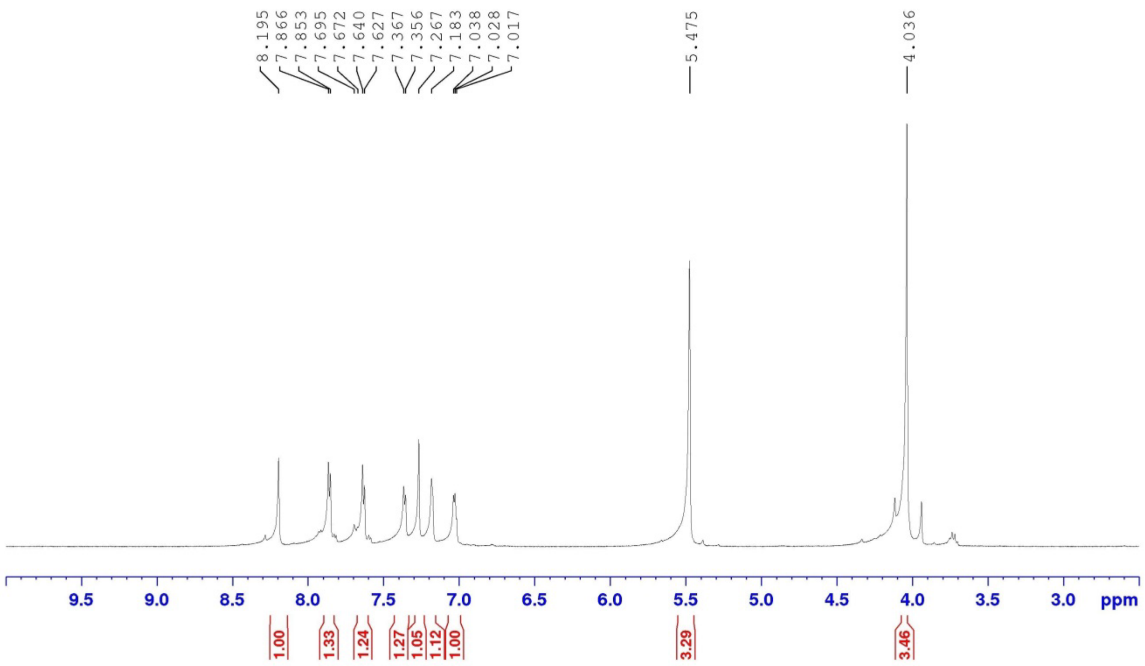

1H NMR Compound 23
DMSO + D2O

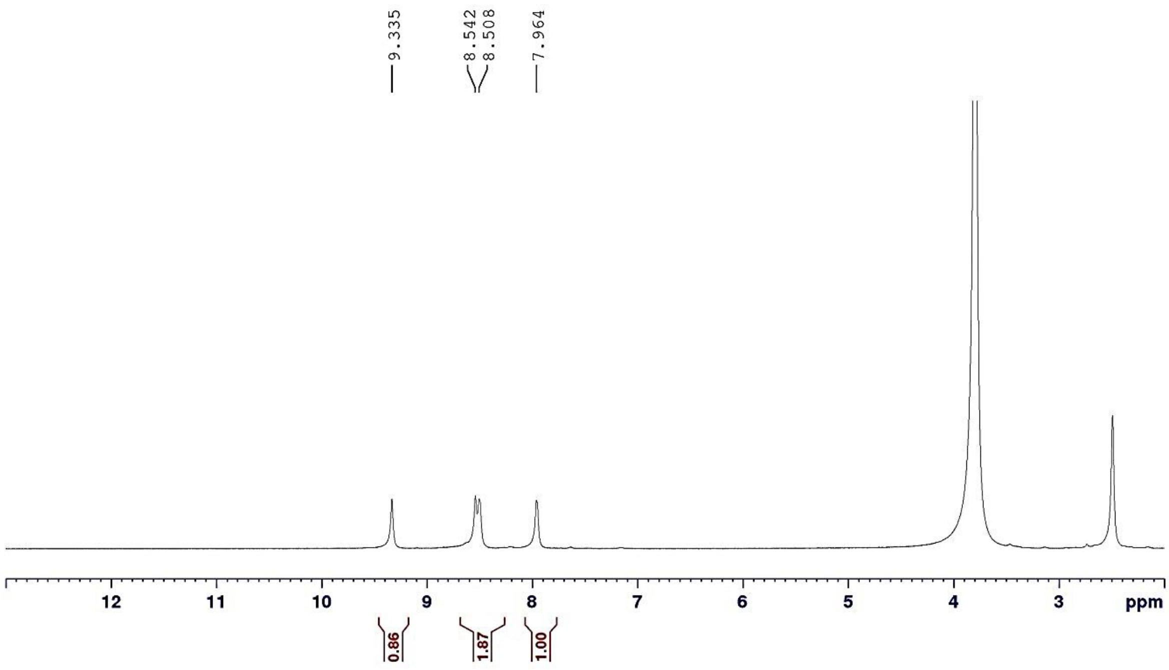

1H NMR Compound 24
DMSO

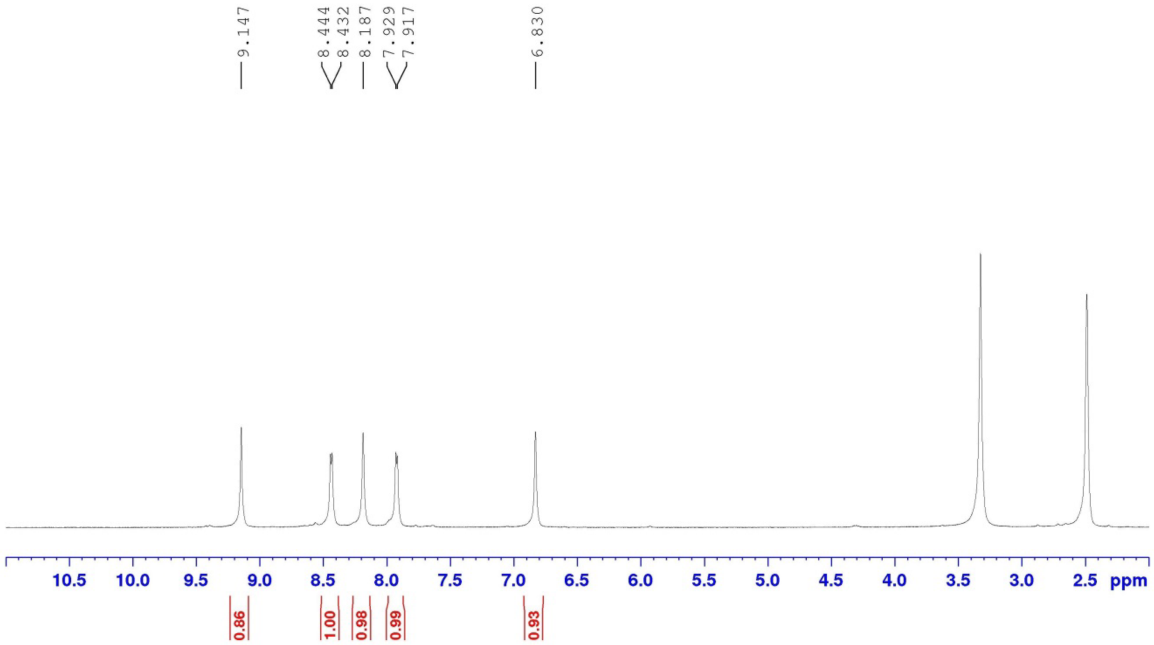

1H NMR Compound 25
DMSO

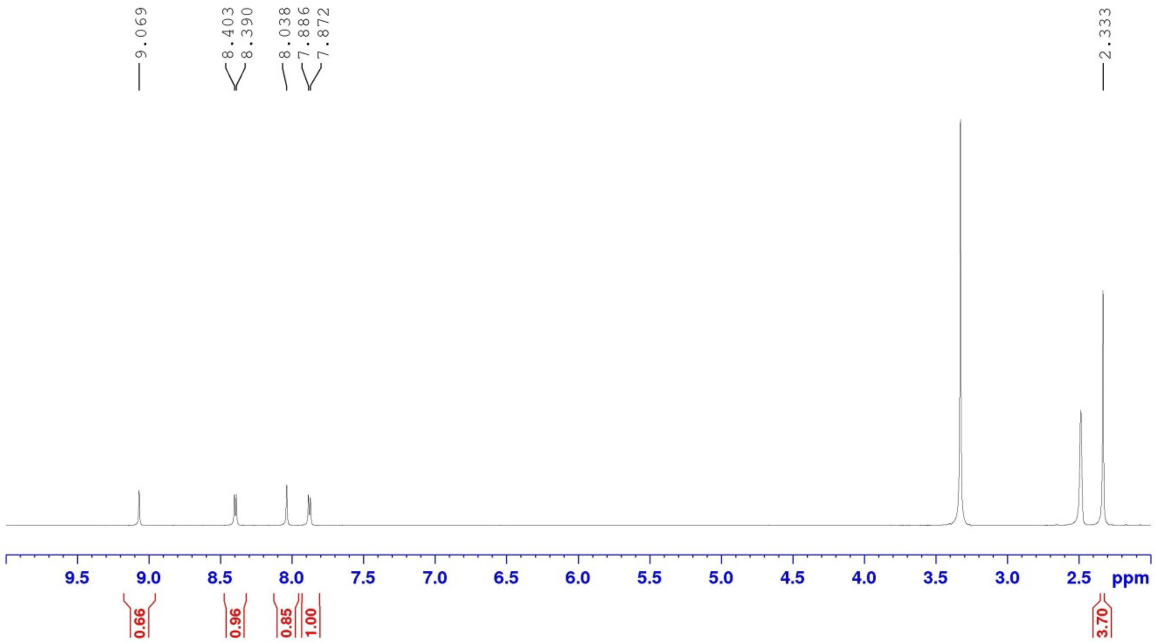

13C NMR Compound 6
DMSO

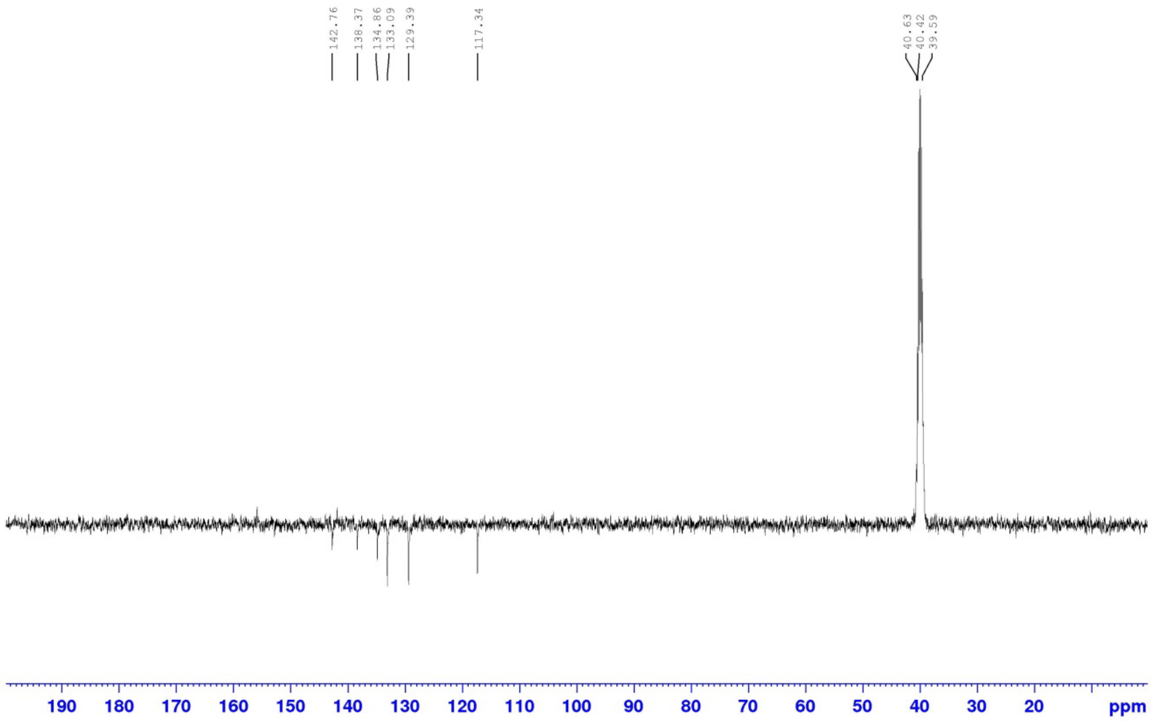

13C NMR Compound 8
DMSO

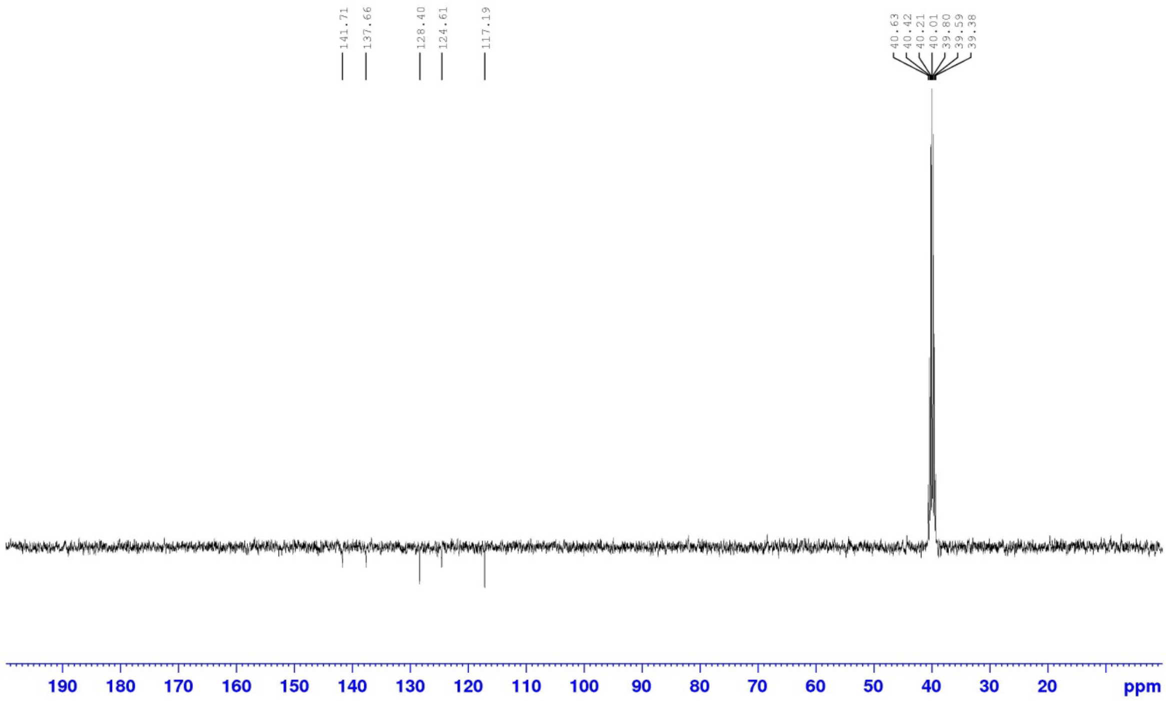

13C NMR Compound 10c
DMSO

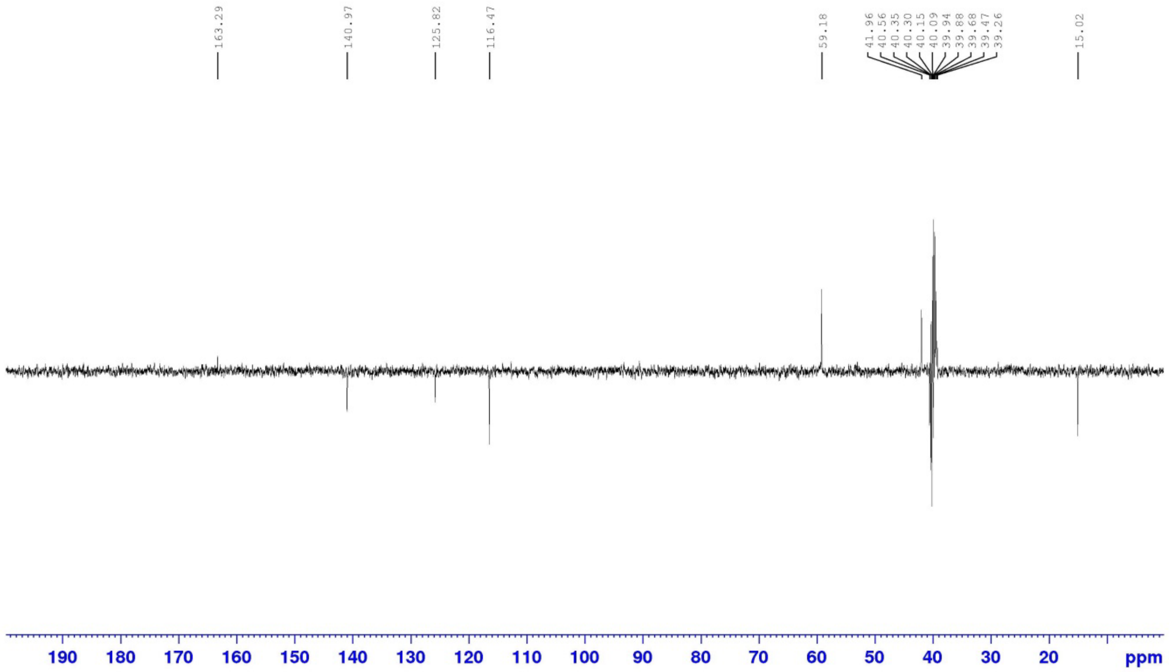

13C NMR Compound 11c
DMSO

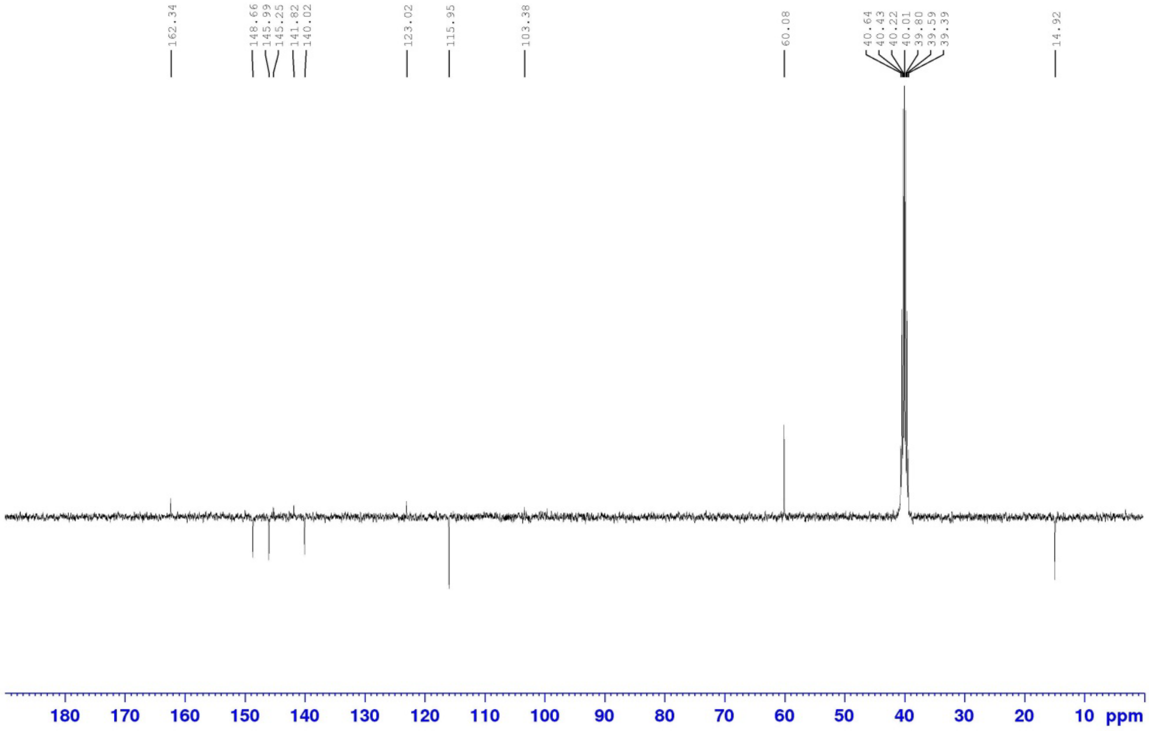

13C NMR Compound 11e
DMSO

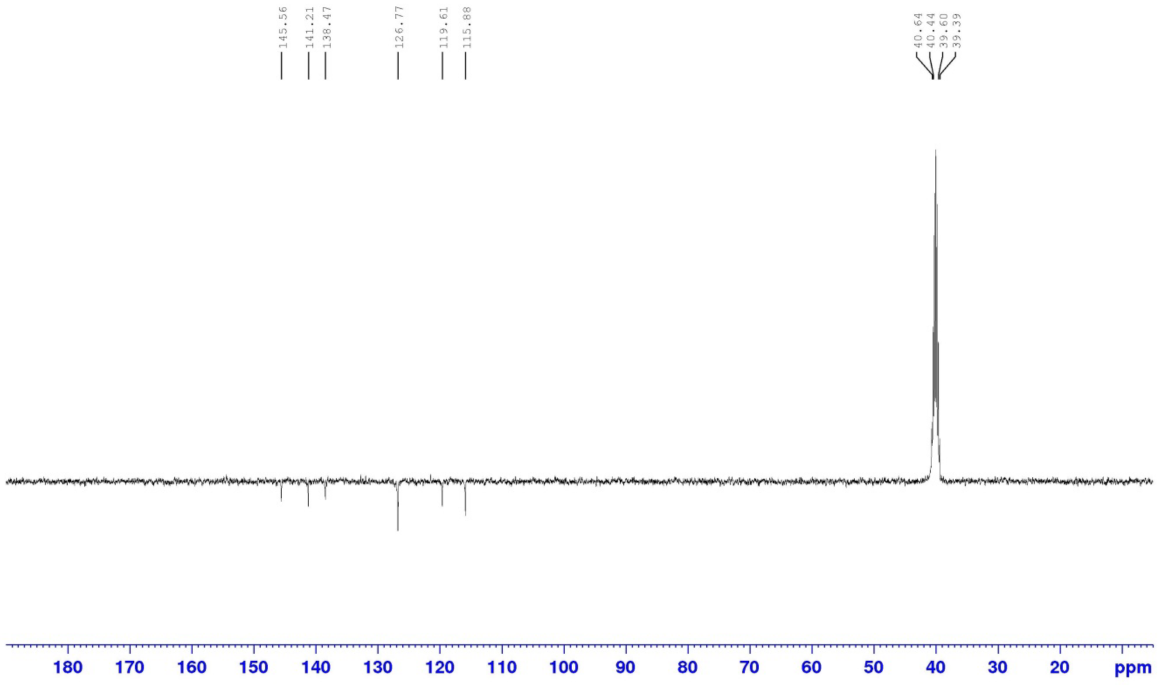

13C NMR Compound 12c
DMSO

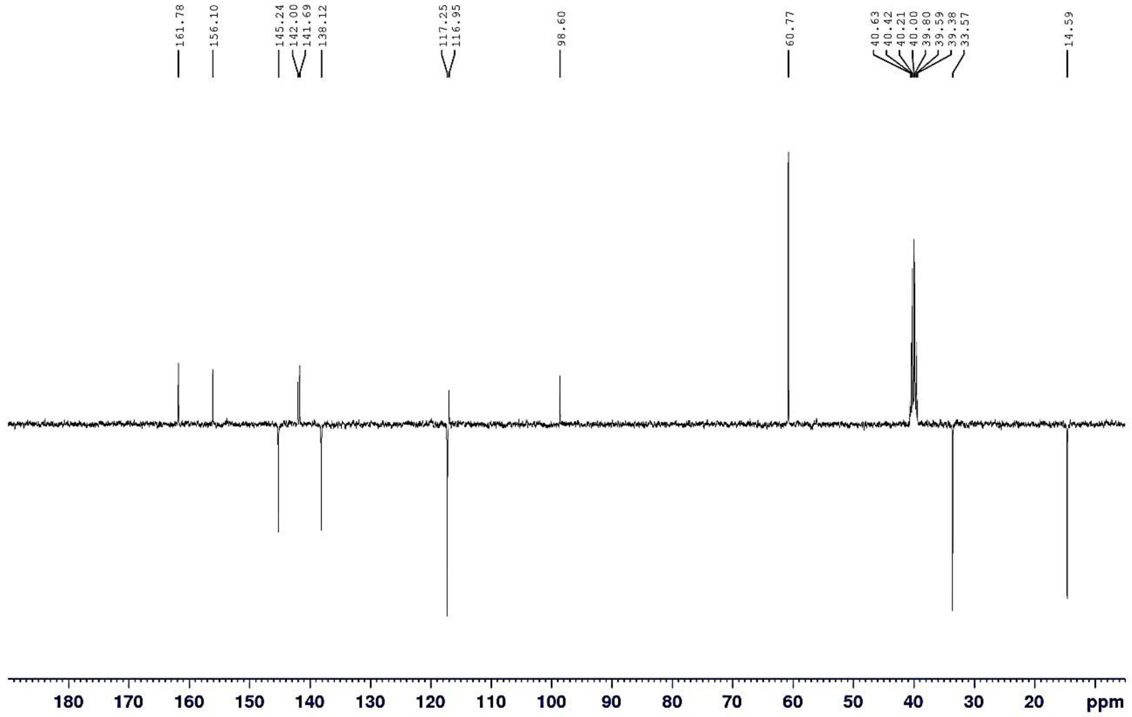

13C NMR Compound 12d
DMSO

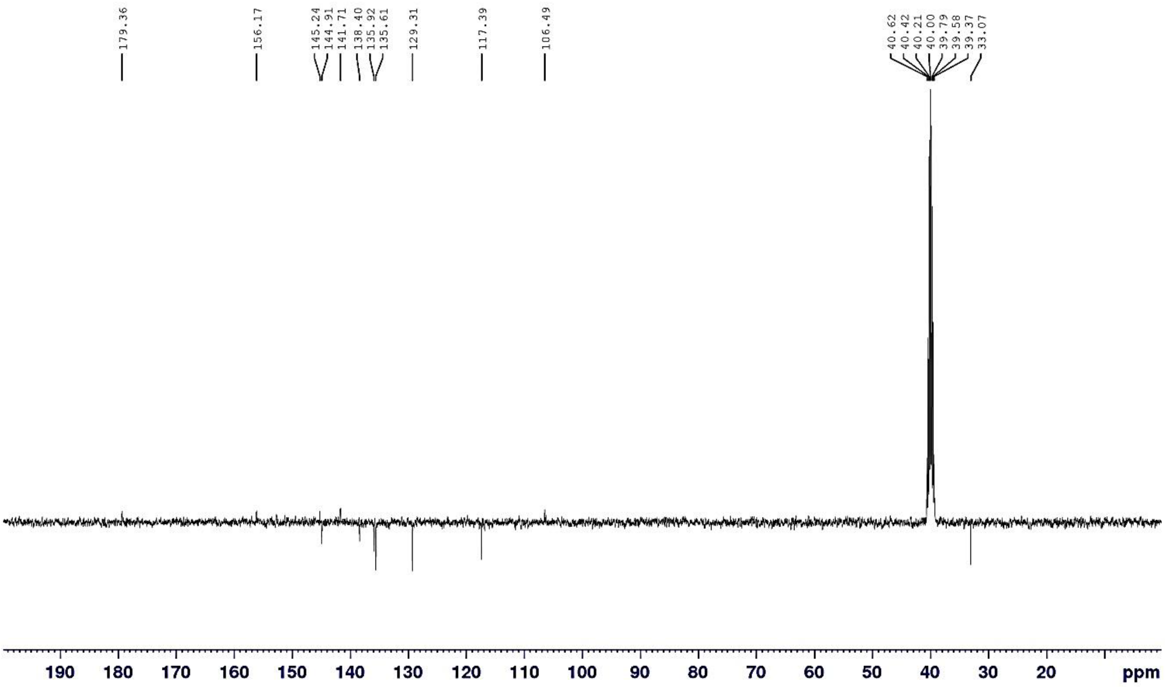

13C NMR Compound 12e
DMSO

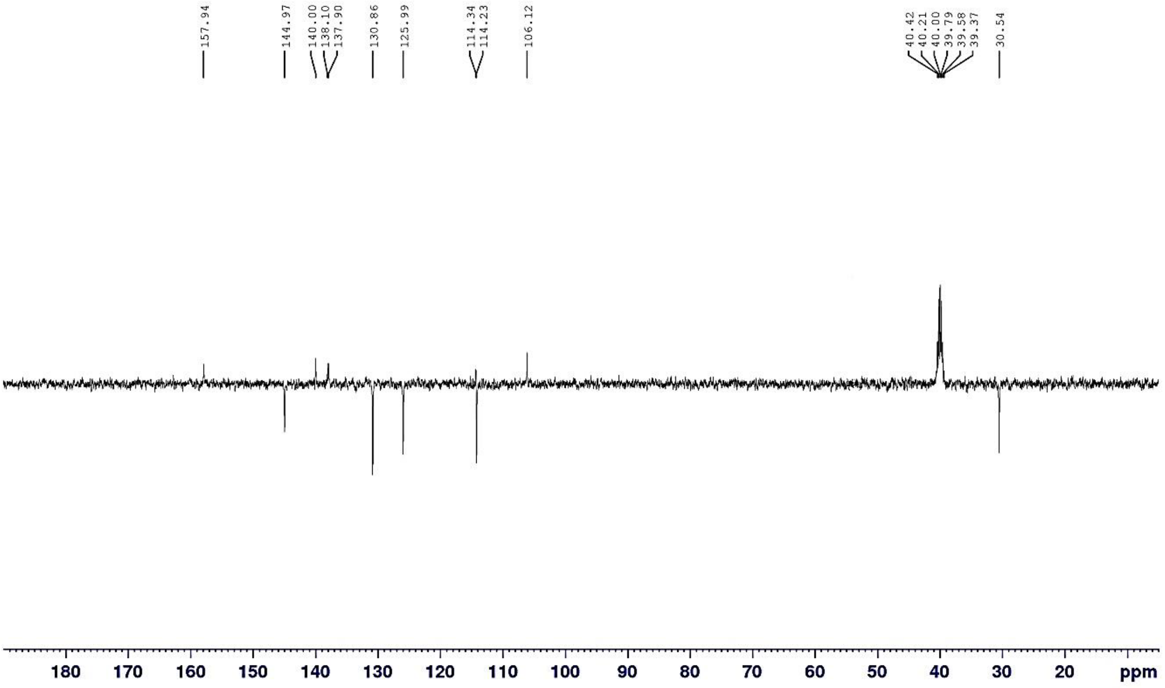

13C NMR
Compound 12f
DMSO

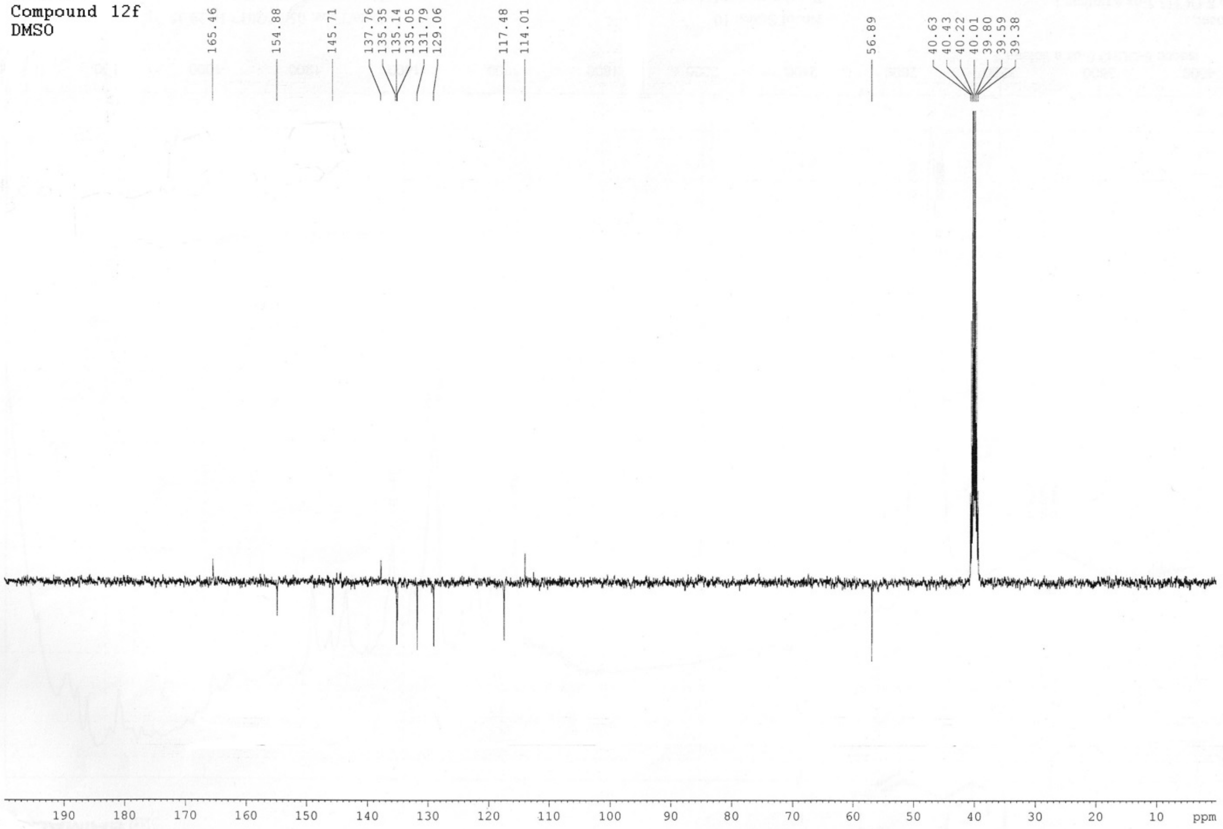

13C NMR Compound 22a
DMSO

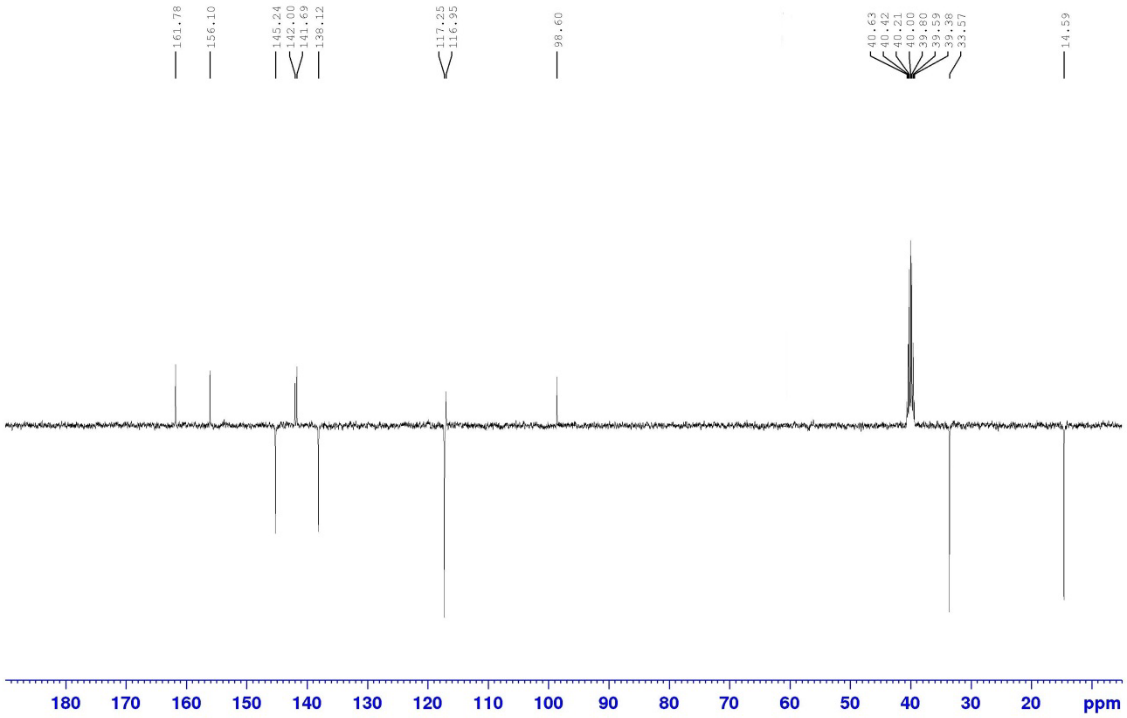

13C NMR Compound 22b
CDCl3

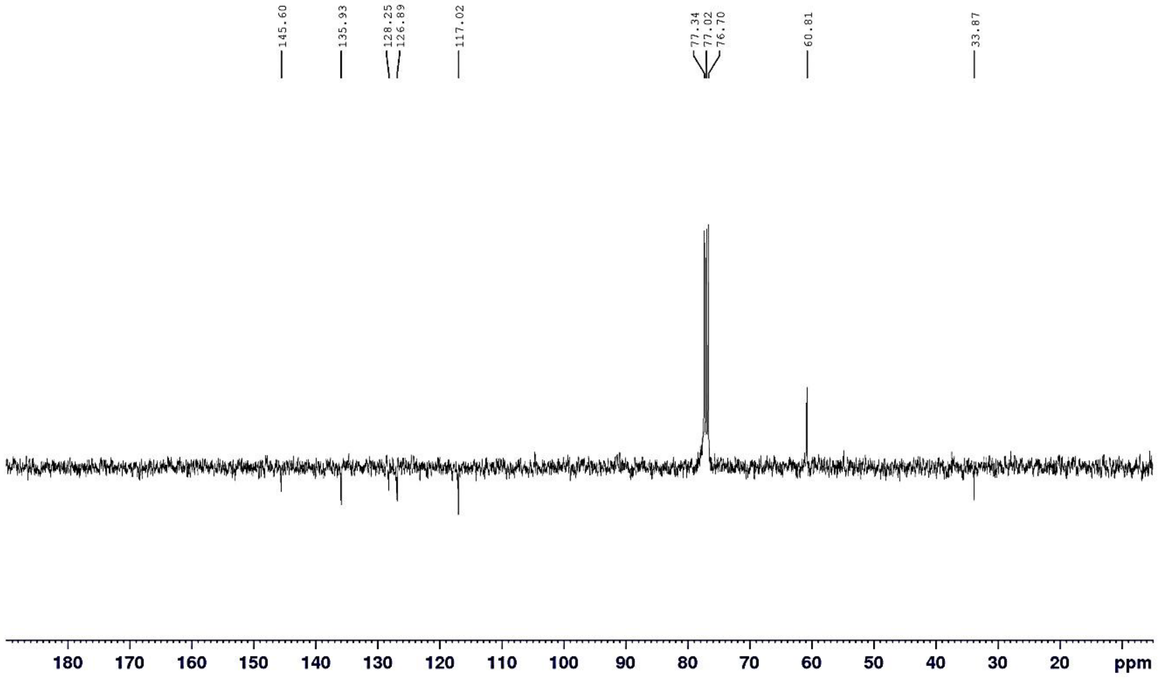

13C NMR Compound 23
CDCl3

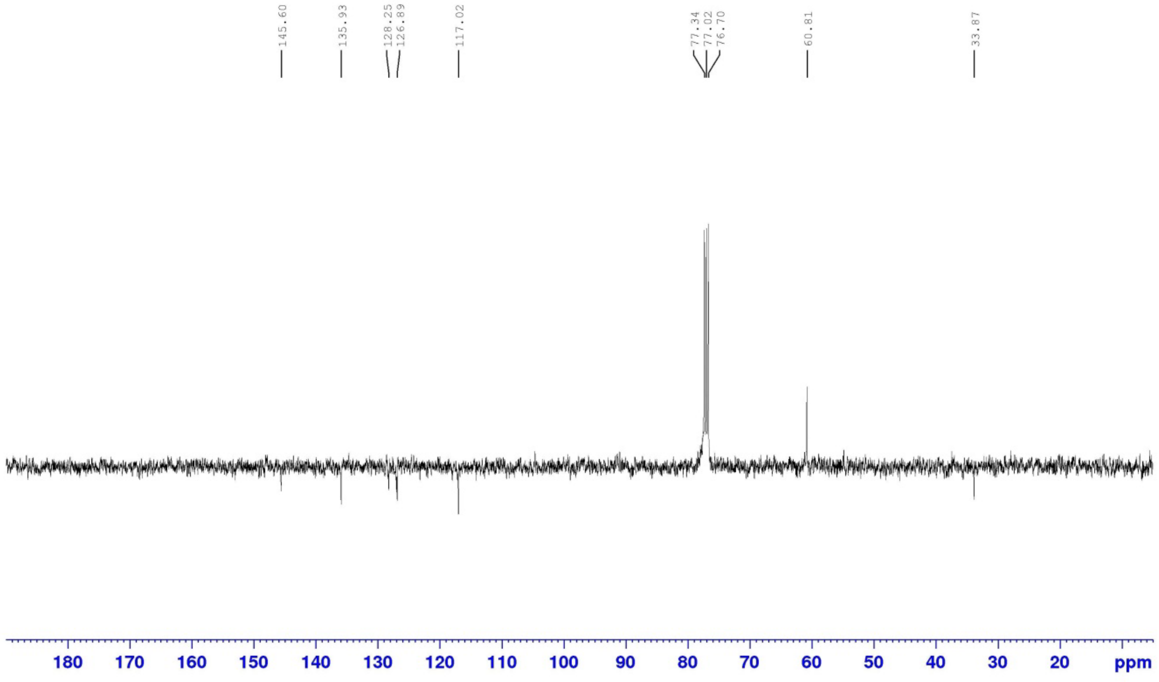

Table S1. Elemental analysis

	Anal. Calcd.			Anal. Found		
**Comp N°**	**C**	**H**	**N**	**C**	**H**	**N**
**3**	49.99	1.86	25.91	49.79	1.85	25.80
**4**	46.15	2.58	23.92	46.33	2.59	24.01
**5**	50.18	3.45	15.96	50.38	3.46	16.02
**6**	51.81	2.34	13.94	51.60	2.33	13.88
**7**	52.73	2.58	15.37	52.52	2.57	15.31
**8**	52.73	2.58	15.37	52.52	2.57	15.31
**9c**	46.90	2.86	14.92	46.71	2.85	14.86
**9d**	48.83	1.89	13.14	49.02	1.90	13.19
**10c**	53.00	4.45	16.86	53.21	4.47	16.93
**10d**	54.34	3.16	14.62	54.55	3.17	14.68
**11c**	53.43	3.67	16.99	53.21	3.65	16.92
**11d**	54.72	2.47	14.73	54.94	2.46	14.79
**11e**	56.01	2.74	16.33	56.23	2.75	16.39
**12a**	52.16	2.63	24.33	52.37	2.64	24.43
**12b**	48.38	3.25	22.57	48.19	3.23	22.48
**12c**	51.98	4.00	15.15	51.77	3.98	15.09
**12d**	53.32	2.88	13.32	53.53	2.89	13.37
**12e**	54.34	3.16	14.62	54.55	3.17	14.68
**12f**	54.34	3.16	14.62	54.12	3.15	14.56
**13b**	48.38	3.25	22.57	48.19	3.23	22.48
**13e**	54.34	3.16	14.62	54.55	3.17	14.68
**13f**	54.34	3.16	14.62	54.12	3.15	14.56
**14**	45.96	2.14	17.86	45.77	2.13	17.79

**15**	50.25	2.64	21.98	50.45	2.65	22.06
**16**	52.67	3.44	20.47	52.46	3.43	20.39
**17**	46.15	2.58	23.92	46.33	2.57	23.82
**18**	46.15	2.58	23.92	46.33	2.57	23.82
**19**	48.19	2.83	16.86	48.38	2.84	16.93
**20**	51.49	3.02	18.02	51.28	3.00	17.95
**21**	48.19	2.83	16.86	48.38	2.84	16.93
**22a**	55.07	4.95	13.76	55.29	4.97	13.81
**22b**	52.16	3.21	12.17	52.37	3.22	12.22
**23**	49.31	2.30	19.17	49.50	2.29	19.25
**24**	54.84	2.88	23.98	54.62	2.87	23.88
**25**	57.12	3.73	22.21	57.35	3.74	22.30

General chemical structure with atoms numeration

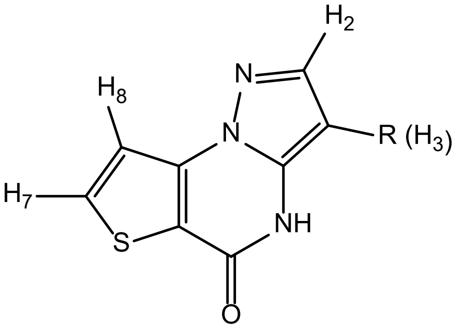

Structure A

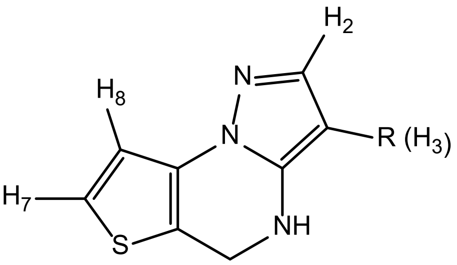

Structure B

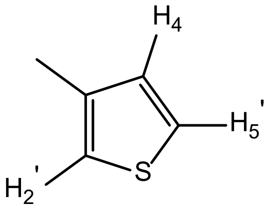

R = 3-thienyl

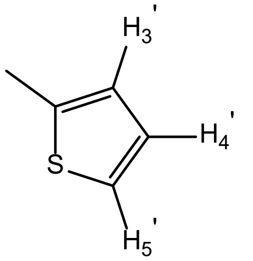

R = 2-thienyl,

thien-2-ylcarbonyl
$COOCH_2$-2-thienyl

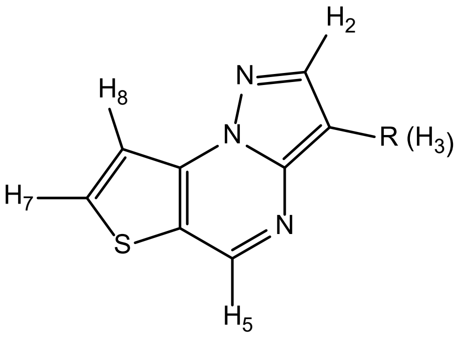

Structure C
